# Ancestral *BG1* Alleles and Structural Conservation Ensure Immune-Related Genetic Resilience in Southeast Asian Chicken Lineages

**DOI:** 10.3390/ani16091398

**Published:** 2026-05-03

**Authors:** Anh Huynh Luu, Trifan Budi, Worapong Singchat, Chien Tran Phuoc Nguyen, Thitipong Panthum, Nivit Tanglertpaibul, Kanithaporn Vangnai, Aingorn Chaiyes, Chotika Yokthongwattana, Chomdao Sinthuvanich, Orathai Sawatdichaikul, Kyudong Han, Narongrit Muangmai, Darren K. Griffin, Prateep Duengkae, Ngu Trong Nguyen, Kornsorn Srikulnath

**Affiliations:** 1 Animal Genomics and Bioresource Research Unit (AGB Research Unit), Faculty of Science, Kasetsart University, 50 Ngamwongwan, Chatuchak, Bangkok 10900, Thailand; anhhuynh.l@ku.th (A.H.L.); trifan.bu@ku.th (T.B.); ntpchien@ctu.edu.vn (C.T.P.N.); thitipong.pant@ku.ac.th (T.P.); nivit.t@ku.th (N.T.); aingorn.ch@ku.ac.th (A.C.); fscicds@ku.ac.th (C.S.); kyudonghan@gmail.com (K.H.); ffisnrm@ku.ac.th (N.M.); d.k.griffin@kent.ac.uk (D.K.G.); prateep.du@ku.ac.th (P.D.); 2 Interdisciplinary Graduate Program in Bioscience, Faculty of Science, Kasetsart University, 50 Ngamwongwan, Chatuchak, Bangkok 10900, Thailand; 3 Faculty of Animal Sciences, College of Agriculture, Can Tho University, 3/2 Street, Ninh Kieu Ward, Can Tho 900000, Vietnam; ntngu@ctu.edu.vn; 4 School of Agricultural Technology, King Mongkut’s Institute of Technology Ladkrabang, Bangkok 10520, Thailand; 5 Special Research Unit for Wildlife Genomics (SRUWG), Department of Forest Biology, Faculty of Forestry, Kasetsart University, 50 Ngamwongwan, Chatuchak, Bangkok 10900, Thailand; 6 Department of Food Science and Technology, Faculty of Agro-Industry, Kasetsart University, 50 Ngamwongwan, Chatuchak, Bangkok 10900, Thailand; kanithaporn.v@ku.th; 7 The International Undergraduate Program in Bioscience and Technology, Faculty of Science, Kasetsart University, 50 Ngamwongwan, Chatuchak, Bangkok 10900, Thailand; 8 Department of Biochemistry, Faculty of Science, Kasetsart University, 50 Ngamwongwan, Chatuchak, Bangkok 10900, Thailand; fscicks@ku.ac.th; 9 Department of Nutrition and Health, Institute of Food Research and Product Development, Kasetsart University, Bangkok 10900, Thailand; orathai.saw@ku.th; 10 Department of Microbiology, College of Bio-Convergence, Dankook University, 119 Dandae-ro, Dongnam-gu, Cheonan-si 31116, Chungnam, Republic of Korea; 11 Bio-Medical Engineering Core Facility Research Center, Dankook University, 119 Dandae-ro, Dongnam-gu, Cheonan-si 31116, Chungnam, Republic of Korea; 12 Smart Animal Bio Institute, Dankook University, 119 Dandae-ro, Dongnam-gu, Cheonan-si 31116, Chungnam, Republic of Korea; 13 Department of Fishery Biology, Faculty of Fisheries, Kasetsart University, 50 Ngamwongwan, Chatuchak, Bangkok 10900, Thailand; 14 School of Biosciences, University of Kent, Canterbury, Kent CT2 7NJ, UK; 15 Biodiversity Center Kasetsart University (BDCKU), Bangkok 10900, Thailand

**Keywords:** avian immune-related genetics, purifying selection, *MHC-B* architecture, ancestral allelic retention, structural conservation

## Abstract

Chicken (*Gallus gallus domesticus*) domestication, likely associated with dry-rice farming in central Thailand, has led to substantial loss of ancestral immune-related genetic diversity in commercial chicken lineages. This study addresses allelic loss by providing the first comprehensive analysis of the highly polymorphic *BG1* gene, an *MHC*-linked marker across the wild–domestic interface in Thailand and Vietnam, using high-depth Illumina amplicon sequencing. Genomic DNA from 47 Thai and Vietnamese chicken populations was extracted using a salting-out protocol following ethical sampling. Allelic variation was examined by targeting the *BG1* intron 15–exon 16 region using triplicate PCR and Salus Pro NGS sequencing. Evolutionary dynamics and selection pressures were analyzed using AmpliSAS, MrBayes, and Datamonkey, while AlphaFold 3 was used to predict and validate 3D protein structures. We identified 98 novel alleles and 172 polymorphic sites within the *BG1* intron 15–exon 16 region encoding an Ig-like domain. Extensive allele sharing between indigenous chickens and red junglefowl indicated strong balancing selection and trans-species polymorphism. Selection analyses showed that purifying selection conserved structural integrity at codons 9, 13, and 18, while variation at other sites enhanced immune recognition. AlphaFold 3 modeling confirmed conservation of the β-sandwich fold across variants, maintaining stability of the Immunoreceptor Tyrosine-based Inhibition Motif (ITIM). Thus, despite the regional gene flow, geographic isolation has shaped distinct signatures, as evidenced by the presence of 38 unique Thai and 9 unique Vietnamese alleles in addition to breed-specific private markers in the Betong (BG1*TH88), Decoy (BG1*TH91), and Tre (BG1*VN54) populations. A notable adaptive outlier under positive selection (*ω* = 1.357) was detected in the Dong Tao population, suggesting a recent selective sweep. These findings support the mission of the Siam Chicken Bioresource Project (SCBP) to utilize indigenous breeds as genetic reservoirs and provide a molecular basis for restoring resilience traits in domestic poultry to enhance global food security.

## Simple Summary

The demographic pressures of domestication have caused a severe erosion of immune-related genetic diversity in commercial chicken lineages, significantly increasing their vulnerability to pathogens. To address this deficit, researchers conducted a comprehensive analysis of the highly polymorphic, *MHC*-linked *BG1* gene across 47 wild red junglefowl and indigenous chicken populations in Thailand and Vietnam. The identification of 98 novel alleles demonstrates that these native Southeast Asian populations function as expansive reservoirs of ancestral immune diversity, sustained by intense balancing selection. Although geographic isolation has driven localized genetic signatures, most notably a recent selective sweep in the Dong Tao breed, pervasive purifying selection rigidly conserves the core structural integrity of the *BG1* protein. Ultimately, this research establishes a vital molecular foundation for marker-assisted selection programs, highlighting how the reintegration of ancestral genetic resources can restore disease resilience in commercial poultry and safeguard global food security.

## 1. Introduction

The chicken (*Gallus gallus domesticus*) is the most ubiquitous domestic animal and a primary source of high-quality protein worldwide [1]. The Red Junglefowl (RJF; Gallus gallus) is the definitive principal maternal ancestor; however, a recent transdisciplinary study by Peters et al. [2] has challenged our understanding of its origins. They have reported that chicken domestication was not initiated in deep forest but amidst dry-rice farming in central Thailand during the Neolithic period (ca. 1500 BC). The expansion of cereal cultivation had acted as a catalyst for the initial wild-to-domestic transition. In fact, this evolutionary legacy is preserved in modern village chickens, which currently account for ≤80% of the poultry stock in developing nations [3]. These populations represent an irreplaceable genetic reservoir because they exhibit traits such as localized adaptations and robust resistance to endemic pathogens that are often absent in highly specialized commercial chicken lines [4]. Furthermore, indigenous and local chickens continue to play pivotal roles in food security and rural household income in agro-ecologically based societies of Thailand and Vietnam, where chickens primarily operate under low-input scavenging systems [5]. In Vietnam, these local breeds account for 84% of the total poultry stock [6] and exhibit notable phenotypic diversity and adaptability to the varied climatic zones of the country. Similarly, indigenous and local Thai breeds possess a long history of adaptation to heat stress and regional farming environments, which has resulted in a genetically diverse population with high value for sustainable agriculture [7]. The resilience of chickens may be primarily attributed to their complex immune-related genetic architecture, the most notable feature of which is the major histocompatibility complex (*MHC*) located on chromosome 16. Particularly, the *MHC-B* locus has been identified as a critical determinant of survivability, and specific haplotypes confer varying degrees of resistance to severe viral challenges such as Marek’s disease [8]. The molecular mechanisms underlying this diversity, however, remain incomplete without accounting for the *BG1* gene, which resides within this complex and acts as a highly polymorphic marker of adaptive avian evolution.

The major histocompatibility complex-B (*MHC-B*) in chicken is fundamentally defined as a “minimal essential” complex that comprises a highly condensed and diverse immune-related genetic region spanning approximately 242 kb on chromosome 16 [9]. This compact architecture includes the *BF/BL*, *TRIM*, and *BG* sub-regions, which collectively determine infectious disease resistance. While the classical *BF* and *BL* loci present peptides to T cells to initiate targeted immune responses [10]. However, the *BG* multigene family is characterized by extreme polymorphisms and broad expression across both immune and epithelial cells, and it plays a specific role in avian health because of which it has thus been intensely studied [11]. The *BG1* gene is particularly significant in this regard. It is situated near the *BF/BL* region and occurs as an isolated and highly variable member of this family. Unlike other *BG* genes, *BG1* exhibits extensive allelic variation that is directly linked to viral resistance and pathogenic outcomes. For example, a specific 225-bp insertion in the *BR4* haplotype has been associated with increased susceptibility to lymphoma, which suggests that *BG1* plays a decisive role in disease progression. Structurally, *BG1* isoforms are unique among *MHC-B* genes because they may contain an Immunoreceptor Tyrosine-based Inhibition Motif (ITIM). This motif has been hypothesized to be a key regulatory element in determining host resistance to oncogenic viruses such as Marek’s disease and Rous sarcoma virus [12]. However, despite its critical role in immune modulation, our understanding of the manner in which these structural motifs vary across unselected indigenous populations remains limited, which necessitates an extensive investigation of *BG1* molecular evolution and *BG1* tertiary protein architecture.

In this study, we primarily address the issue of the significant depletion of allelic diversity in domestic chicken lineages. This erosion may be attributed to centuries of artificial selection and demographic bottlenecks. As a result, modern indigenous chickens potentially possess only a fraction of the immunological repertoire of their wild ancestors. Although the significance of the *BG1* locus in disease resistance is well known, the roles of geographic dispersal barriers and localized pathogen repertoires in shaping the genetic architecture of this gene across the wild–domestic interface remain unknown. Therefore, we primarily posit that RJF and unselected indigenous populations across Thailand and Vietnam retain an expansive diversity of *BG1* genotypes, which has been maintained through intense balancing selection driven by regional environmental pressures. Furthermore, we hypothesize that independent variables such as physical distance and species-specific dispersal patterns influence the genetic structure of these populations by dictating the distribution of dependent variables such as allelic variation and polymorphic density of the *BG1* locus. We have addressed these questions by characterizing the *BG1* gene comprehensively, integrating Salus Pro short-read sequencing with advanced computational modeling. Furthermore, we aimed to identify ancestral alleles that may have been lost in commercial poultry lines by examining a broad geographic transect of RJF and indigenous and local chicken populations. Additionally, we used AlphaFold 3 to investigate the manner in which selective pressures balance sequence divergence with the requirement for structural conservation in the encoded immune proteins. Overall, we aimed to identify essential genetic resources that may be used to enhance poultry immunity through molecular marker-assisted selection. Additionally, we aim to provide information for global conservation strategies and the development of resilient breeding programs.

## 2. Materials and Methods

### 2.1. Animal Sampling and Genomic DNA Isolation

Blood samples were collected from a total of 470 individuals across 47 populations located in various places in Thailand and Vietnam (Table S1). Specifically, this cohort comprised 368 indigenous and local chickens (169 from Vietnam and 199 from Thailand) representing 35 distinct populations, as well as 102 RJF (13 from Vietnam and 89 from Thailand) representing 12 populations. Sampling was performed only after obtaining prior consent from farm owners, and all birds were immediately released back into their original environment. Whole blood was drawn from the brachial vein using Vacuette^®^ 21-gauge needles and transferred into tubes containing 5 mM EDTA (Greiner Bio-One, Kremsmünster, Austria). The tubes were maintained at 4 °C until required for laboratory processing. Genomic DNA was extracted by following the standard salting-out protocol described by Supikamolseni et al. [13]. To ensure that high-quality templates were obtained for analysis, DNA concentration and integrity were assessed, and the absence of significant degradation was verified using a NanoDrop 2000 spectrophotometer (Thermo Fisher Scientific, Waltham, MA, USA) and 1% agarose gel electrophoresis. All procedures complied with the Animal Experimentation Regulations of Kasetsart University and ARRIVE guidelines (arriveguidelines.org), based on which the experimental protocols were reviewed and approved (Approval code: ACKU67-SCI-021).

### 2.2. Gene Identification and Target Region Selection for Polymorphic Analysis

The *BG1* gene was initially identified by screening the in-house whole-genome sequencing (WGS) data against homologous sequences of various chicken breeds that were retrieved from public databases (Table S2). Preliminary comparative analysis showed that the genomic architecture comprised 16 exons and 15 introns. Therefore, intron 15 and exon 16 were selected as the primary target region because they contain a high density of informative polymorphic sites that are highly suitable for both allelic diversity analysis and phylogenetic inference.

### 2.3. Targeted BG1 Amplification and Salus Pro Short-Read Sequencing 

A genomic fragment comprising parts of intron 15 and exon 16 of the *BG1* gene exhibits significant polymorphism within Galliformes. This portion was amplified using the primer pair pcBG1F (5′-TGGAGCGGCACAGGGTGAGT-3′) and pcBG1R (5′-GGGCTGCAACCACCCCAGTT-3′) [14]. To facilitate multiplexing and sample identification, unique 8-bp barcode sequences were appended to the 5′ end of each forward primer (Macrogen Inc., Seoul, Republic of Korea). Each 15-µL PCR reaction contained approximately 25 ng of genomic DNA prepared using 1× Apsalagen buffer (1.5 mM MgCl_2_), 0.2 mM dNTPs, 0.5 μM of each primer, and 0.5 U of Taq DNA polymerase (Apsalagen Co., Ltd., Bangkok, Thailand). The thermal cycling profile included an initial denaturation at 94 °C for 5 min, followed by 35 cycles of 94 °C for 30 s, 62 °C for 30 s, and 72 °C for 30 s, and a final extension at 72 °C for 5 min. All PCR products were visualized on 2% agarose gels to confirm successful amplification. Each sample was processed in triplicate to enhance data reliability and minimize the detection of false alleles, which occur during the amplification of multigene families. Finally, the barcoded amplicons were pooled into six multiplexed libraries and subjected to paired-end sequencing on a Salus Pro medium-throughput NGS platform (Shenzhen Salus BioMed Co., Ltd., Shenzhen, China) using a 2 × 250 bp paired-end (PE250) configuration, at the Kasetsart–Salus Biomed–Gibthai Collaborative Excellence Center (Bangkok, Thailand) to obtain high-depth coverage required for robust allelic calling.

### 2.4. Sequence Processing, Quality Control, and Genotype Assignment

The raw paired-end reads showed an average length of 379 bp. These were initially subjected to quality assessment using FASTQC version 0.12.0 [15]. After quality control, the paired reads were merged and demultiplexed into individual sequence sets based on their unique barcode groups. The amplicon sequences were processed using AmpliSAS version 1.0 [16], an automated pipeline specifically designed for multigene family genotyping that includes robust, built-in algorithms for chimera removal and sequencing artifact filtering. To ensure the exclusion of sequencing artifacts and maintain high data reliability, we implemented a minimum read-depth threshold of 100 reads per amplicon. Furthermore, the maximum number of alleles per individual was capped at six, which accounts for the potential presence of duplicated *BG1* loci in the chicken genome [17]. To distinguish true alleles from background noise, we applied the Degree of Change (DOC) parameter [18], which identifies legitimate sequences based on characteristic read-depth distribution patterns. All other AmpliSAS pipeline parameters were maintained at their default settings. For sequence characterization, each identified allele was evaluated via BLASTn Version 2.17.0 searches against the NCBI database (http://blast.ncbi.nlm.nih.gov/Blast.cgi, accessed on 02 December 2025) to determine sequence similarity with existing records. Then, the nucleotide sequences were aligned and translated using Geneious Prime version 2024.0.5 (https://www.geneious.com/), which confirmed the absence of premature stop codons within the exon 16 coding region. All novel allele sequences identified in this study have been deposited in the GenBank database (https://www.ncbi.nlm.nih.gov/) under accession numbers PX984255–PX984352 (accessed on 28 February 2026).

### 2.5. Evolutionary Relationship Modeling and Allelic Network Analysis

To elucidate the evolutionary relationships among *BG1* alleles, a phylogenetic tree was constructed using Bayesian inference in MrBayes version 3.2.6 [19]. The optimal nucleotide substitution model was selected using ModelFinder [20] within the IQ-TREE framework, which identified the best-fit model based on the lowest Bayesian information criterion (BIC). Markov Chain Monte Carlo (MCMC) analysis was executed with four chains for one million generations, from which trees were sampled every 100 generations after reaching stationarity. A majority-rule consensus tree with mean branch lengths was generated after discarding 10% burn-in (100,000 generations). To provide a global context, partial *BG1* sequences from various chicken breeds were retrieved from the NCBI database for performing a comparative analysis of the intron 15 and exon 16 regions. The final tree was visualized using the Interactive Tree of Life (iTOL) version 5 (https://itol.embl.de/) [21]. Genetic differentiation among the studied populations was examined using principal coordinate analysis (PCoA) based on allele frequency data. Pairwise genetic distances were computed using the poppr package in R version 4.5.2 [22], which provided a robust framework for assessing population structure. Genotypic relationships were inferred using the median-joining network implemented in PopART version 1.7 [23]. This network was constructed from aligned nucleotide sequences. The nodes represent genotypes, and node sizes correspond to their observed frequencies. The connections and hash marks between nodes represent mutational steps, and they enable a clear visualization of genotype sharing and population-specific variants.

### 2.6. Genetic Diversity Estimation and Inference of Molecular Selective Pressures 

Genetic diversity in the studied populations was assessed by estimating the number of alleles (*N*_a_) and nucleotide diversity (*π*) using DnaSP version 6.12 [24]. To evaluate the genetic structure of Vietnamese and Thai indigenous chickens and RJF, we performed an analysis of molecular variance (AMOVA) using the poppr package in R version 4.5.2 [22,25]. The average rates of synonymous (*dS*) and nonsynonymous (*dN*) substitutions per site were calculated via the Nei–Gojobori method with Jukes–Cantor correction. This enabled a *Z*-test to be performed to evaluate the *dN/dS* ratio (*ω*). Within this framework, *ω* > 1 suggested positive selection, whereas *ω* < 1 indicated purifying selection. To assess positive selection, we analyzed lineages with elevated *ω* estimates as foreground branches using the Branch-Site Unrestricted Statistical Test for Episodic Diversification (BUSTED) on the Datamonkey server. Significance (*p* < 0.05) was determined via an LRT comparing unconstrained (*ω* > 1) and null (*ω* ≤ 1) evolutionary models [26]. The selective pressures were further investigated by using DnaSP to perform neutrality tests such as Tajima’s *D*, Fu’s *F*, and Fu and Li’s F* and D* to identify deviations from neutral evolution based on the allelic frequency spectra. To account for multiple comparisons across the diverse studied populations, the raw *p*-values from all neutrality tests were adjusted using the Benjamini–Hochberg False Discovery Rate (FDR) method using R version 4.5.2 [22]. Statistical significance was strictly defined using a *q*-value threshold of *q* < 0.05. Specific selection signals across *BG1* were identified by analyzing the codon alignments (98 sequences and 28 codons) using the Datamonkey web server (https://www.datamonkey.org/). We used a combined methodological approach that was designed to robustly capture both intermittent and constant selective pressures. Episodic positive selection was determined using the mixed effects model of evolution (MEME), which used a likelihood ratio test (LRT) at a significance threshold of *p* < 0.01 [27]. Pervasive selection was detected using two complementary methods: fixed effects likelihood (FEL), which estimated substitution rates at each site across the phylogeny (*p* < 0.01), and fast unconstrained Bayesian approximation (FUBAR), which provided a computationally efficient Bayesian inference for weak selection with a posterior probability ≥ 0.9 [28]. This comprehensive framework ensured the robust detection of both intermittent and constant selective pressures acting on *BG1* codon sites. 

### 2.7. AlphaFold 3-Based Tertiary Structure Prediction and Functional Domain Alignment

The amino acid sequences derived from the partial exon 16 region of the *BG1* gene were used to predict tertiary (3D) protein structures using the AlphaFold 3 prediction server version AF3 (https://alphafoldserver.com/), which is a state-of-the-art framework for high-accuracy molecular modeling [29]. The obtained structural models were visualized by BIOVIA Discovery Studio version 24.1 (Dassault Systèmes, San Diego, CA, USA) and subjected to quality assessment using PROCHECK version 3.5.4 [30]. As a part of this evaluation, Ramachandran plot analysis was performed to verify the stereochemical quality and conformational stability of the predicted residues. High-confidence models were aligned and compared with a reference lectin-like natural killer cell surface protein that was retrieved from the UniProt database (https://www.uniprot.org/) to identify conserved structural domains and functional similarities.

## 3. Results

### 3.1. Characterization of BG1 Genomic Architecture and Target Selection

The screening of the in-house WGS data against public databases helped identify the *BG1* gene as a highly variable candidate locus (Table S2 and Figure S1). Genomic mapping showed that the *BG1* gene architecture comprises 16 exons and 15 introns. Comparative sequence analysis of 44 sequences from 28 breeds and 1 RJF individual indicated that nucleotide variation was not uniformly distributed. Intron 15 and exon 16 exhibited a disproportionately high level of polymorphism compared to those of other regions. Specifically, this intronic and exonic segment yielded 58 variable sites within a 453-bp region, which is a high density of informative markers. The observed transition/transversion ratio and the presence of 17 unique SNPs within this region have confirmed its suitability for high-resolution allelic differentiation. Consequently, this target was prioritized for downstream phylogenetic inference because of its ability to resolve the fine-scale genetic relationships between indigenous breeds and RJF populations.

### 3.2. Characterization of BG1 Allelic Repertoires and Population-Specific Signatures

High-throughput short-read sequencing generated a total of 15,104,372 raw paired-end reads. Following stringent quality control, demultiplexing, and chimera filtering, 1208 high-quality reads were retained. The average sequencing depth for the successfully called alleles was 557X, which robustly exceeded our established minimum threshold of 100 reads. Eventually, 379-bp partial sequences of the *BG1* gene were obtained, including the junction between partial intron 15 and exon 16. The predicted secondary structure of the encoded exonic region indicated a complex architecture that contains α-helix-turn-β-strand and coil motifs (Figure S2). In total, 98 unique *BG1* alleles comprising 172 polymorphic sites were identified and aligned with the reference sequence (Accession No. OM953775). Among the Vietnamese populations, 60 distinct alleles (BG1*01–BG1*60) were identified, and the majority of these were identified in indigenous and local breeds. Notably, a specific subset of alleles that included BG1*VN-TH1 and BG1*VN-TH2 (Table S3) was shared with RJF. This indicates a significant degree of ancestral polymorphism retention. However, the alleles BG1*VN-TH52 and BG1*VN-TH53 were exclusive to the *G. gallus* spadiceus population in Kien Giang (Tables S3 and S4), which highlights potential lineage-specific genetic signatures in these wild populations. Among the Thai chicken populations, a diverse repertoire of alleles was identified, and most of these were identified exclusively in indigenous and local breeds (Tables S3 and S5). These included a wide range of variants such as BG1*VN-TH4, BG1*VN-TH11, and the BG1*TH73-98 series, which exhibited highly restricted breed-specific distribution patterns (Table S5). For example, alleles BG1*VN-TH18 and BG1*TH98 were detected solely in the Lao Pa Koi population, whereas the others were unique to the Betong (BG1*VN-TH35, BG1*TH86, and BG1*TH88) and Phuphan black (BG1*VN-TH49) breeds. Similarly, localized variants were identified in the Mae Hong Son (BG1*TH74–76 and BG1*TH83), Decoy (BG1*TH91), and Dong Tao in Udon Thani (BG1*TH93) populations. These findings highlight the high degree of genetic isolation among these local lineages. Moreover, four distinct alleles (BG1*VN-TH37, BG1*VN-TH56, BG1*VN-TH59, and BG1*TH63) were unique to the RJF. These wild-type alleles exhibited clear geographic localization, as evidenced by the restriction of BG1*VN-TH37 to Roi Et, BG1*VN-TH56 to Sa Kaew, and BG1*VN-TH59 to Songkhla and Chiang Mai Zoo. Other populations such as those in Chanthaburi and Si Sa Ket were characterized by the presence of BG1*TH63, which suggests that *BG1* allelic diversity is strongly influenced by regional environmental or demographic factors.

**Figure 1:**
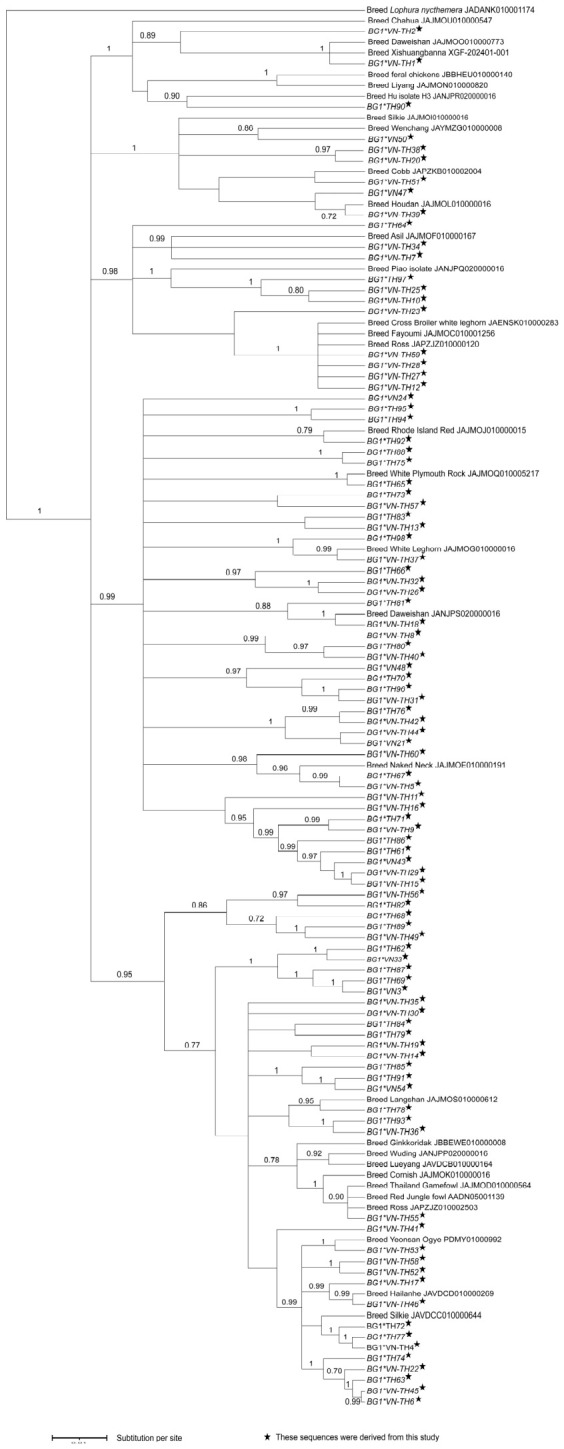
Bayesian phylogenetic tree of partial intron 15 and exon 16 of the *BG1* gene alleles from Thai and Vietnamese indigenous and local chicken breeds and red junglefowl. The values above the branches represent posterior probability. The scale indicates substitutions per site.

**Figure 2:**
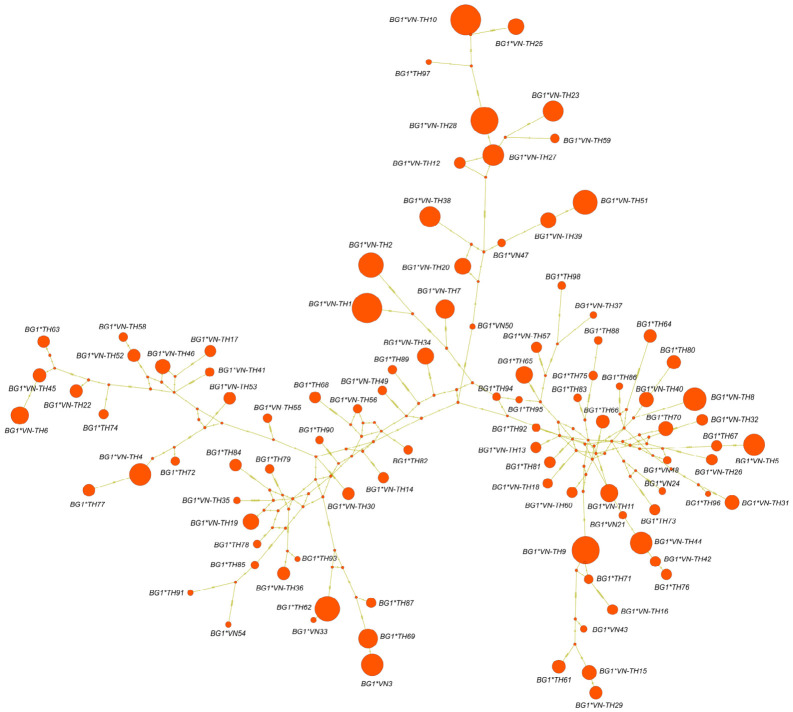
Allelic network of the *BG1* gene identified in Thai and Vietnamese indigenous and local chicken breeds and red junglefowl. Each circle represents an allele, and the circle size is proportional to its frequency in the population. Lines connecting the alleles indicate mutational steps, and short bars on the branches represent inferred mutational events.

In comparison with the reference sequence, the 98 identified alleles comprised 28 exonic variants, including 11 synonymous and 17 nonsynonymous mutations, along with 144 intronic variants (Table 1 and Table S6). Allelic richness (*AR*) varied across populations, with the number of alleles ranging from 2–25, whereas the mean *π* remained consistent between regions (Vietnam: 0.056; Thailand: 0.057; Table 2). Specifically, indigenous and local breeds harbored up to 19 alleles in Vietnam and 23 in Thailand. In comparison, RJF populations exhibited a higher number of alleles ranging from 5–25 alleles and yielded mean *π* values that reflected high genetic plasticity within wild lineages (Vietnam: 0.051; Thailand: 0.057). Bayesian phylogenetic inference indicated a polyphyletic topology that was characterized by a distinct lack of clustering based on breed or geographic origin (Figure 1). This absence of distinct population structure was further supported by the AMOVA results, which showed that the majority of genetic variation was distributed between individuals (76.0% in Vietnam and 88.9% in Thailand) rather than between populations (24.0% in Vietnam and 11.1% in Thailand) (Table S7). Moreover, the PCoA indicated that the first two principal coordinates explained only 8.1% and 5.7% of the total variance, which further corroborated the high level of genetic admixture across the region (Figure S3). The allelic network showed the intricate relationships among the 98 *BG1* alleles and highlighted a central cluster of high-frequency alleles that were shared between indigenous breeds and RJF in both countries (Figure 2). This shared core includes variants such as BG1*VN-TH1 and BG1*VN-TH51, and it contrasts with the numerous low-frequency alleles that occupy the peripheral branches (Figure 2). Notably, geographic isolation was evident in specific subsets, including nine alleles that were identified exclusively in Vietnam and 38 alleles (BG1*TH61 to BG1*TH98) that were unique to Thai populations (Table S3).

### 3.3. Evidence of Selective Pressures and Evolutionary Conservation of BG1 Gene

The codon-based *Z*-test showed that the *BG1* gene has been predominantly subject to purifying selection across most indigenous chicken breeds and RJF populations in Vietnam and Thailand (Table 3). Mean *ω* (*dN/dS*) values were estimated at 0.469 and 0.435 for Vietnamese and Thai indigenous chickens, respectively, which closely mirror the values observed in RJF populations (Vietnam: 0.407; Thailand: 0.371). These *ω* < 1 results suggest that purifying selection plays a dominant role in maintaining the genetic stability of this lineage. The Dong Tao population from Udon Thani, Thailand, yielded an *ω* estimate of 1.357. However, likelihood ratio testing confirmed that this deviation was not statistically significant (LRT = 0.000, *p* = 0.500), indicating that the locus remains predominantly under purifying or neutral constraints even within this isolated cohort. By contrast, while overall *ω* values remained below unity, episodic positive selection was detected in specific lineages. Notably, the Tre (An Giang1) population exhibited an overall *ω* of 0.476, but yielded a statistically significant Likelihood Ratio Test (LRT = 6.203, *p* = 0.022). This indicates that while the locus as a whole is functionally constrained, a proportion of sites within this specific lineage is undergoing significant episodic positive selection. Furthermore, the neutrality test results helped characterize the population-level variation despite Tajima’s *D* values showing no statistical significance in either the Vietnamese (−0.707 to 0.201) or Thai (−1.015 to 1.342) populations (Table 4). Similarly, Tajima’s *D*, Fu and Li’s D*, and Fu and Li’s F* statistics were generally non-significant, which suggests a lack of recent population expansion or severe bottlenecks for most groups. Following the application of the FDR correction to account for multiple testing, no individual population exhibited statistically significant deviations (*q* > 0.05). These results suggest an absence of recent, severe population bottlenecks or rapid selective sweeps that are strong enough to overcome the region-wide balancing selection acting upon this locus. At the codon level, MEME analysis identified 28 codons that evolved under neutral selection (Table S8). By contrast, multiple tests for pervasive negative selection highlighted strong evolutionary conservation in specific functional regions. According to the FEL analysis results, codons 9 and 13 have undergone significant purifying selection (*p* < 0.01), which indicates strong functional constraints at these sites (Table S9). This finding was corroborated by the results of FUBAR analysis, which identified three sites (codons 9, 13, and 18) that had evolved under negative selection with high posterior probabilities (*p* > 0.9) (Table S10).

### 3.4. Structural Characterization and Comparative Modeling of BG1 Protein Variants

**Figure 3:**
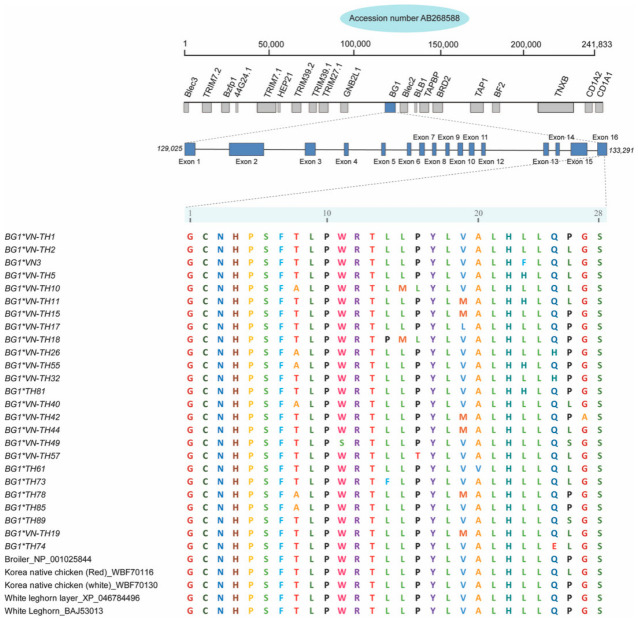
*BG1* protein sequence alignment of indigenous chicken populations in Thailand and Vietnam with sequences from various chicken breeds in the NCBI database.

The amino acid sequences corresponding to the *BG1* gene exhibited high similarity to the *Gallus gallus* reference sequence (Accession No. WBF70130) with sequence identities ranging from 98.8–100% and query coverage ranging from 94–100%. The analyzed region corresponded to nucleotide positions 133,192–133,277 within exon 16, which encodes a 28-amino acid segment (residues 6–33) situated within the signal and Ig-like domains. Comparative analysis confirmed that these *BG1* alleles maintained 98.8–100% sequence identity with the Broiler, Korean Native, and White Leghorn breeds (Figure 3). This finding indicated high conservation across diverse chicken lineages. The tertiary (3D) structures of the predicted peptide variants were characterized and clustered into five representative models (Figure S4), which were validated through Ramachandran plot analysis. Model 2 exhibited superior stereochemical quality with 100% of the residues in the most favored regions, followed by Model 4 at 90.9%. A separate subset comprising Models 1, 3, and 5 showed favored region values ranging from 76.2–85.7%, which suggested that structural variance was likely influenced by the specific amino acid composition of each allele. All models were structurally aligned with the reference “signal peptide and Ig-like protein” (Accession: B5BSR2; Figure S5), which confirmed the functional identity of the analyzed protein region.

## 4. Discussion

The characterization of the *BG1* locus across the Thai–Vietnamese corridor shows a dynamic evolutionary landscape defined by high allelic plasticity and significant ancestral retention. The results of the current study indicate the presence of a critical balance between extensive nucleotide diversity and strict conformational conservation, which enables pervasive purifying selection to maintain the structural integrity of the Ig-like domain. This interplay supports the “minimal essential” *MHC* hypothesis and simultaneously identifies localized adaptive outliers to provide a unique perspective on the molecular evolution of avian immunity.

### 4.1. Ancestral Polymorphism Retention and Allelic Plasticity Across Wild–Domestic Interface

The analysis of the 379-bp *BG1* partial sequence (intron 15 and exon 16) showed notably high levels of polymorphism across both national cohorts, as evidenced by the presence of 98 alleles and 172 polymorphic sites. Despite geographic separation, the maintenance of genetic diversity was notably consistent among the regions, which suggests that both populations have retained significant ancestral variation. Immunologically, maintaining this polymorphism equips indigenous flocks with a diverse repertoire of *BG1* alleles capable of binding a wide array of antigenic peptides. This molecular diversity serves as a population-level buffer, providing the specific immune receptors necessary to combat novel or rapidly mutating endemic pathogens. This parity is a hallmark of *MHC*-like regions, where balancing selection actively maintains diversity to maximize immunological potential. Furthermore, this hypervariability aligns with the standard architecture of the *MHC-B* region, which has been evolutionarily designed for broad pathogen recognition [31]. Additionally, the presence of specific *BG1* variants in resistant phenotypes is consistent with its hypothesized role in the immune response to MDV, though further functional validation is required. This implies that free-ranging indigenous breeds show extensive diversity as a result of the evolutionary necessity for survival against diverse environmental pathogens [12]. A defining feature of this diversity is the presence of alleles that are shared between distinct indigenous breeds and RJF. This provides robust evidence of transspecies polymorphism because this phenomenon occurs when ancestral allelic lineages predate the divergence of domestic chickens from their wild progenitors [32]. However, the persistence of these lineages is driven by balancing selection, which contrasts with directional selection by preventing the fixation of single advantageous alleles [33]. Moreover, the extensive allele sharing identified in the present study supports the hypothesis of continuous gene flow and introgression between wild *Gallus gallus* and domestic chickens. This validates that Southeast Asia was the primary center of chicken diversification.

Despite this evidence of shared ancestry, the localized evolutionary forces that are simultaneously at work must be acknowledged, as evidenced by the 38 unique alleles identified in the Thai population compared with the nine identified in the Vietnamese population. These unique variants act as molecular signatures, such as BG1*VN54 in Tre and BG1*TH98 in Lao Pa Koi for specific breeds, and indicate that local adaptation to regional pathogens is driving the divergence of new variants. Thus, these dual dynamics preserve ancestral lineages while generating novel variants. Additionally, it ensures that the avian immune system remains robust against both ancient and emerging threats. Analysis of the wild ancestor *G. gallus gallus* has shown that a distinct genetic signature has been retained via private alleles (BG1*TH63), which supports the hypothesis that the RJF subspecies preserve their unique genetic identities in spite of extensive hybridization with domestic populations. Significantly, the *BG1* gene exhibited a striking lack of overall population structure, which was confirmed by the results of Bayesian phylogenetic and PCoA. Unlike neutral loci, the weak population structure observed here is a meaningful evolutionary signature for immune genes. AMOVA reveals that most genetic variance exists between individuals within a population (76.0% in Vietnam and 88.9% in Thailand) rather than between populations (24.0% and 11.1%, respectively). This partitioning is a hallmark of balancing selection and trans-species polymorphism, which prevents allele fixation to maintain heterozygote advantage. The evolutionary demand for immunological diversity overrides the geographic and demographic forces that typically isolate these populations. This partitioning of variance provides strong evidence that the locus is under balancing selection, whereby heterozygosity is evolutionarily advantageous [34]. Furthermore, the high level of within-individual variation suggests that the heterozygote advantage is the primary evolutionary force that shapes the *BG1* landscape and facilitates the maintenance of a diverse allelic repertoire that is required for host survival in pathogen-rich environments. 

### 4.2. Evolutionary Constraints and Structural Integrity of BG1 Ig-like Domain

The *BG1* gene exhibited a predominant landscape of purifying selection across indigenous breeds and RJF in Vietnam and Thailand. Complementary selection analyses indicate that the core structural integrity of the *BG1* protein is under strict functional constraint. Specifically, the Ig-like domain appears to operate under strict molecular requirements such that mutations that disrupt its highly conserved fold are deleterious to host fitness [4]. Furthermore, statistical modeling identified specific residues that exhibited high resistance to evolutionary change, particularly in codons 9, 13, and 18. Mechanistically, these constrained hydrophobic residues are critical for preserving the core *β*-sandwich architecture; these residues likely correspond to buried hydrophobic cores or invariant cysteine bridges that are essential for the correct folding and stability of the receptor [31]. Additionally, the structural integrity of our homology models, which was rigorously assessed via Ramachandran plot analysis, confirmed that the majority of the generated models exhibited high stereochemical quality. Specifically, model 2 achieved optimal geometry with 100% of the residues situated in the most favored regions. Other models, such as model 4, exceeded the 90% threshold, which is typically required for high-quality structures according to PROCHECK standards [30]. By contrast, a subset of models (1, 3, and 5) exhibited favored region values that represented a variance that may be attributed to specific amino acid polymorphisms that were inherent to these alleles. Notably, all models showed significant structural homology to the signal peptide and Ig-like protein template (Accession: B5BSR2), which confirmed the fact that the globally conserved Ig-like β-sandwich fold has been maintained across all variants, irrespective of local allelic variation. This architectural constancy suggests a “framework model” of evolution, where the gene preserves a rigid structural scaffold to ensure stability while simultaneously permitting plastic variability at 28 episodic sites to maximize immune recognition breadth [35]. 

In addition to selection pressure, neutrality tests highlighted the remarkable stability of this locus across diverse demographic landscapes. Following FDR correction, no individual population within the Vietnamese or Thai cohorts exhibited statistically significant deviations in Tajima’s *D* or Fu and Li’s statistics. While raw *p*-values initially yielded significantly negative values in specific Thai groups, suggesting an excess of rare alleles, which typically serves as a genomic signature of population expansion following a bottleneck or a selective sweep [36], the FDR correction revealed these deviations to be artifacts of multiple testing. Biologically, this uniform state of mutation-drift equilibrium is a profound finding. This suggests that the pervasive balancing selection acting on the *BG1* locus is sufficiently robust to maintain continuous allelic diversity. Consequently, this stability effectively buffers the genetic architecture against the localized demographic bottlenecks, habitat fragmentation, and intensive breeding interventions that routinely skew neutrality tests in commercial poultry. Moreover, owing to the compact nature of the chicken *MHC-B* region, this signature may have resulted from a genetic hitchhiking, where the *BG1* locus is influenced by its close physical linkage to a neighboring gene that has undergone a rapid selective sweep [31]. This pattern suggests that Thai breeds may have been subjected to more intensive recent breeding interventions or habitat fragmentation than their Vietnamese counterparts. This contrasts with the high genetic diversity that is typically observed in neutral markers such as microsatellites in these same breeds [37]. This coexistence of rigid conservation at key residues and rapid evolution at other sites suggests that the *BG1* gene possesses the adaptive capacity to respond to localized environmental pressures without compromising its core molecular function.

### 4.3. Adaptive Outliers: Localized Selection and Structural Mutations at the BG1 Locus

The Dong Tao population from Udon Thani, Thailand, showed a notable divergence from the regional trend of purifying selection, as evidenced by the *ω* value of 1.357. Although this ratio mathematically implies positive selection, the signal is not statistically significant. Small sample sizes (*n* = 5) artificially inflate substitution rate variance, creating statistical artifacts that mimic directional selection. Consequently, rather than indicating a rapid adaptive sweep, the Dong Tao population reflects the broader regional pattern of purifying and neutral constraints. By contrast, robust statistical modeling successfully identified true signals of localized adaptation. The Tre (An Giang1) population yielded a highly significant BUSTED result despite a low overall *ω* average. This apparent paradox perfectly illustrates the framework model of immune receptor evolution. While pervasive purifying selection acts on the majority of the sequence to maintain the structural *β*-sandwich core, episodic positive selection is simultaneously driving rapid, adaptive mutations at specific localized sites. This suggests the Tre population from An Giang1 is actively adapting its immune repertoire in response to strong, localized pathogenic pressures within its specific geographic niche. Nevertheless, if representative, this outlier likely reflects a rapid adaptive sweep that may have been triggered by the localized pressures of a specific disease outbreak or the demographic consequences of a founder effect following the relatively recent introduction of the breed from Vietnam. Nevertheless, the accelerated evolution at the *BG1* locus highlights its capacity for rapid immune-related genetic remodeling within localized lineages and provides a potential genetic basis for breed-specific disease resistance [4]. Another key finding in this population was the 302G > A mutation, which causes a Proline-to-Leucine (P > L) substitution. Proline possesses a unique cyclic side chain that imposes the rigid backbone constraints required to stabilize secondary structures such as *β*-turns. However, leucine introduces significant conformational flexibility [38]. Therefore, this substitution may possibly destabilize the cytoplasmic tail, which would prevent the ordered presentation of the ITIM to phosphatase SHP-2. We hypothesize that this structural failure could potentially abrogate inhibitory signaling and theoretically confer disease resistance by blocking the ability of the pathogen to exploit immune checkpoints [39]. While our in silico predictions identify the 302G > A variant as a candidate for altered ITIM signaling, experimental validation, which must include both in vitro assays and in vivo challenge models, is required to confirm its functional impact on host immunity and disease resistance. Additionally, the loss of structural constraints often leads to the unfolding of surface loops and alteration of antigenic epitopes. These changes drive *MHC* diversification through balancing selection, which enhances host survival against a broad spectrum of pathogens. Furthermore, the detected positive selection may be additionally attributed to the intense selective breeding practices applied to Dong Tao chickens to maintain their highly specialized morphological traits, such as their characteristically enlarged feet [40]. A comparative analysis of mutations that affect protein function showed a distinct genetic divergence among the studied populations. The Vietnamese population represents the ancestral reservoir, which possesses the most complex regulatory framework that is characterized by a triad of functional mutations: 311A > T (Leu to His), 312G > A (Leu to Phe), and 302G > A (Pro to Leu). This ancestral complexity is maintained across a specific set of alleles, including BG*VN3, BG*VN-TH7, and BG*VN-TH11. However, the DT2 population (Lopburi) has adopted a diverse generalist strategy. This group has lost the ancestral 312 mutation but has retained the 311A > T and 302G > A variants, which have enabled the evolution of the largest set of allelic variants (e.g., BG1*VN-TH5, BG*VN-TH14, and BG*TH87). In fact, the DT1 population (Udon Thani) appears to be most genetically streamlined, which suggests a specialized adaptation. This population relies solely on the single activation mutation 302G > A (P > L) and expresses its own distinct private alleles such as BG1*VN-TH16 and BG*TH93. This simplification indicates a loss of the fine-tuning regulatory markers found in the ancestral and generalist groups, which potentially reflects a highly focused evolutionary response to specific environmental pressures. Possibly, anthropogenic pressure created a genetic bottleneck that reduced the effective population size and facilitated the fixation of novel *BG1* variants through genetic drift or linked selection. This scenario contrasts with the high levels of *AR* that are generally maintained in larger unselected indigenous populations, as observed in neutral marker assessments of other Thai breeds [37]. However, the Dong Tao population shows a complex interplay between cultural selection and molecular evolution, which suggests that human-mediated trait fixation may inadvertently drive significant shifts in the immune-related genetic landscape of the *MHC-B* region.

### 4.4. Strategic Implications for Conservation, Molecular Breeding, and Study Limitations

The high levels of within-individual variation and identification of breed-specific markers, such as those restricted to the Lao Pa Koi and Betong breeds, highlight the urgent need for regional conservation strategies to prevent the irreversible erosion of rare ancestral alleles. These findings are central to the mission of the Siam Chicken Bioresource Project (SCBP), which aims to characterize and preserve the unique genetic heritage of Southeast Asian poultry [4,37,40]. We believe that integrating these identified ancestral variants into marker-assisted selection programs would create a robust pathway for enhancing poultry immunity in commercial lines by reintroducing the resilience traits that were lost during intensive selection [4]. Additionally, this study reiterates and expands the inference of recent studies that emphasize the role of indigenous breeds as primary genetic reservoirs for future agricultural adaptation [41]. However, this study had some limitations that must be addressed in future studies. Crucially, the inherent difficulties of sampling certain restricted or elusive lineages resulted in small sample sizes (ranging from 3 to 10 individuals) for specific population subsets, such as the Dong Tao (Udon Thani) and certain red junglefowl groups. Small sample cohorts limit statistical power and introduce potential biases, including the non-detection of rare alleles, which could lead to an underestimation of true allelic richness. Small sample sizes can artificially inflate the variance of *dN/dS* ratios. Localized signals of positive selection, such as the one observed in the Dong Tao cohort, carry a high risk of being statistical artifacts rather than true evolutionary shifts, and must be treated strictly as preliminary indicators. Furthermore, small sample sizes can inflate the variance and generate large standard errors when calculating *dN/dS* ratios, meaning that localized signals of positive selection should be treated as preliminary indicators rather than definitive evolutionary proofs. Additionally, although we used high-depth sequencing to characterize the *BG1* gene, the analysis was restricted to the intron 15–exon 16 region, which may not have captured the total selective landscape of the entire 242-kb *MHC-B* complex. Notably, our analysis was restricted to a highly polymorphic 84-bp (28 amino acid) fragment within exon 16, which might not represent the entire *BG1* gene’s selective landscape. Estimates of evolutionary pressure, such as the *dN/dS* ratio, are highly volatile in short sequences, as a single mutation can cause drastic mathematical shifts. Our reported selection signals and *ω* values represent localized snapshots rather than whole-gene trends. In addition, this length restriction limits structural modeling. Although AlphaFold 3 confidently predicted the fragment’s localized *β*-sandwich fold, isolated peptide modeling cannot account for the global stabilizing interactions and conformational constraints of the full-length *BG1* protein. Therefore, the precise biological functions of these allelic variants need to be confirmed through in vitro functional assays. Future studies that expand the genomic coverage to the full *BG1* transcript and incorporate pathogen challenge analysis are essential to definitively link specific allelic signatures with disease resistance phenotypes. Thus, the SCBP aims to bridge these gaps to develop a comprehensive map of immune-related genes that supports the conservation of *Gallus gallus* diversity and helps improve global security of poultry production.

## 5. Conclusions

This study has successfully addressed the issue of allelic erosion in domestic poultry by showing that indigenous chicken breeds and red junglefowl populations across Thailand and Vietnam function as expansive reservoirs of ancestral immune-related genetic diversity. The findings presented here confirm the central hypothesis that the *BG1* locus maintains extreme allelic plasticity, as evidenced by the identification of 98 novel alleles that have been preserved through intense balancing selection despite the demographic pressures of domestication. The identification of exceptional polymorphisms and extensive allele sharing between wild and domestic cohorts provides robust evidence of trans-species polymorphisms and continuous historical gene flow across the Southeast Asian interface. These results definitively reiterate the relationship between our defined variables as follows: although independent variables such as geographic distance, dispersal barriers, and species-specific demographics have driven the distribution of the dependent variable via localized polymorphisms, the core structural integrity of the *BG1* protein remains rigidly conserved. This is evidenced by the fact that the distribution of 38 unique Thai and 9 unique Vietnamese alleles reflects regional isolation, despite which the Ig-like domain is maintained by pervasive purifying selection at codons 9, 13, and 18. Additionally, the detection of adaptive outliers, most notably the recent selective sweep identified in the Dong Tao population, highlights the capacity for rapid immune-related genetic remodeling triggered by founder effects or intense artificial selection. These findings validate the strategic mission of the SCBP to integrate ancestral wild diversity with modern agricultural needs. Overall, this study identifies essential genetic resources that provide a molecular foundation for marker-assisted selection programs aimed at reintroducing lost resilience traits into commercial poultry lines. Additionally, this study provides a global framework for conservation and sustainable breeding to ensure that the immune-related genetic legacy of *Gallus gallus* is utilized to safeguard the future of poultry health and global food security.

## Figures and Tables

**Table 1:** Variable sites of the *BG1* gene alleles found in this study.

Alleles	Accession Number	Variable Nucleotide Position (Accession Number: OM953775)																																	
		Intron 15						Exon 16																											
		Nucleotide Position *																																	
		39	84	173	157	159	175	298	299	301	302	303	304	306	307	310	311	312	320	324	325	332	333	336	337	338	339	340	343	347	349	352	355	357	358
Reference sequence	OM953775	A	G	A	C	T	A	T	C	C	G	G	C	G	G	G	A	G	G	C	G	G	G	G	G	A	G	G	C	C	G	A	G	T	G
BG1*VN-TH1	PX984255	.	.	C	.	.	.	.	.	.	.	.	.	.	.	.	.	.	.	.	.	.	.	.	.	.	.	.	.	.	.	G	.	.	.
BG1*VN-TH2	PX984256	G	C	C	.	.	.	.	.	.	A	.	.	.	.	.	.	.	.	.	.	.	.	.	.	.	.	.	.	.	.	.	.	.	.
BG1*VN3	PX984257	G	.	G	G	.	.	.	.	.	A	.	.	.	.	.	.	A	.	.	A	.	.	.	.	.	.	.	.	.	.	G	.	.	.
BG1*VN-TH4	PX984258	.	.	C	A	.	.	.	.	.	A	.	.	.	.	.	.	.	.	.	.	.	.	.	.	.	.	.	.	.	.	G	.	.	.
BG1*VN-TH5	PX984259	G	.	C	T	A	T	.	.	.	A	.	.	.	.	.	T	.	.	.	.	.	.	.	.	.	.	.	.	.	.	G	.	.	.
BG1*VN-TH6	PX984260	.	.	C	A	.	.	.	.	.	A	.	.	.	.	.	.	.	.	.	.	.	.	.	.	.	.	.	.	.	.	G	.	.	.
BG1*VN-TH7	PX984261	G	.	.	.	.	.	.	.	.	A	.	.	.	.	.	T	.	.	.	.	.	.	.	.	.	.	.	.	.	.	G	.	.	.
BG1*VN-TH8	PX984262	.	.	C	T	A	T	.	.	.	A	.	.	.	.	.	.	.	.	.	.	.	.	.	.	.	.	.	.	.	.	G	.	.	.
BG1*VN-TH9	PX984263	.	.	C	T	A	T	.	.	.	.	.	.	.	.	.	.	.	.	.	.	.	.	.	.	.	.	.	.	.	.	G	.	.	.
BG1*VN-TH10	PX984264	G	.	.	.	.	.	.	.	.	A	.	.	.	.	.	.	.	.	.	.	A	.	T	.	.	.	.	.	.	.	G	.	C	.
BG1*VN-TH11	PX984265	.	.	C	.	A	T	.	.	.	A	.	.	.	.	.	T	.	.	T	.	.	.	.	.	.	.	.	.	.	.	G	.	.	.
BG1*VN-TH12	PX984266	.	.	.	.	.	.	.	.	.	.	.	.	.	.	.	.	.	.	.	.	.	.	.	.	.	.	.	.	.	.	.	.	.	.
BG1*VN-TH13	PX984267	.	.	C	.	A	T	.	.	.	.	.	.	.	.	.	.	.	.	.	.	.	.	.	.	.	.	.	.	.	.	G	.	.	.
BG1*VN-TH14	PX984268	.	.	C	G	.	.	.	.	.	.	.	.	.	.	.	.	.	.	.	.	.	.	.	.	.	.	.	.	.	.	G	.	.	.
BG1*VN-TH15	PX984269	G	.	.	T	A		.	.	.	.	.	.	.	.	.	.	.	.	T	.	.	.	.	.	.	.		.	.	.	G	.	.	.
BG1*VN-TH16	PX984270	.	C	C	.	A	T	.	.	.	A	.	.	.	.	.	.	.	.	.	.	.	.	.	.	.	.	A	.	.	.	G	.	.	.
BG1*VN-TH17	PX984271	.	.	C	A	.	.	.	.	.	.	.	.	.	.	.	.	.	.	G	.	.	.	.	.	.	.	T	.	.	.	G	.	.	.
BG1*VN-TH18	PX984272	.	.	C	.	A	T	.	.	.	.	.	.	.	.	.	.	.	.	.	.	A	.	T	.	G	.	.	.	.	.	G	.	.	.
BG1*VN-TH19	PX984273	.	.	C	A	.	.	.	.	.	A	.	.	.	.	.	.	.	.	T	.	.	.	.	.	.	.	.	.	.	.	G	.	.	.
BG1*VN-TH20	PX984274	G	C	.	.	.	.	.	.	.	.	.	.	.	.	.	.	.	.	.	.	.	.	.	.	.	.	.	.	.	.	.	.	.	.
BG1*VN21	PX984275	.	C	C	.	A	T	.	.	.	A	.	.	.	.	.	.	.	.	T	.	.	.	.	.	.	.	.	.	.	.	G	.	.	.
BG1*VN-TH22	PX984276	.	.	C	A	.	.	.	.	.	A	.	.	.	.	.	.	.	.	.	.	.	.	.	.	.	.	.	.	.	.	G	.	.	.
BG1*VN-TH23	PX984277	.	T	.	.	.	.	.	.	.	A	.	.	.	.	.	.	.	.	.	.	.	.	.	.	.	.	.	.	.	.	.	.	.	.
BG1*VN24	PX984278	.	.	C	T	A	T	.	.	.	A	.	.	.	.	.	.	.	.	.	.	.	.	.	.	.	.	.	.	.	.	G	.	.	.
BG1*VN-TH25	PX984279	.	.	.	.	.	.	.	.	.	A	.	.	.	.	.	.	.	.	.	.	A	.	T	.	.	.	.	.	.	.	G	.	C	.
BG1*VN-TH26	PX984280	G	.	C	T	A	T	.	.	.	.	.	G	.	.	.	.	.	.	.	.	.	.	.	.	.	.	.	.	.	.	.	.	C	A
BG1*VN-TH27	PX984281	.	T	.	.	.	.	.	.	.	.	.	.	.	.	.	.	.	.	.	.	.	.	.	.	.	.	.	.	.	.	.	.	.	.
BG1*VN-TH28	PX984282	.	T	.	.	.	.	.	.	.	.	.	.	.	.	.	.	.	.	.	.	.	.	.	.	.	.	.	.	.	.	.	.	.	.
BG1*VN-TH29	PX984283	G	.	.	T	A	.	.	.	.	.	.	.	.	.	.	.	.	.	T	.	.	.	.	.	.	.	.	.	.	.	G	.	.	.
BG1*VN-TH30	PX984284	.	.	C	A	.	.	.	.	.	.	.	.	.	.	.	.	.	.	.	.	.	.	.	A	.	.	.	.	.	.	G	.	.	.
BG1*VN-TH31	PX984285	G	.	C	T	A	.	.	.	.	A	.	.	.	.	.	.	.	.	.	.	.	.	.	.	.	.	.	.	.	C	G	.	.	.
BG1*VN-TH32	PX984286	G	.	C	T	A	T	.	.	.	.	.	G	.	.	.	.	.	.	.	.	.	.	.	.	.	.	.	.	.	.	C	.	.	.
BG1*VN33	PX984287	.	.	.	G	.	.	.	.	.	A	.	.	.	.	.	.	.	.	T	.	.	.	.	.	.	.	.	.	.	.	G	.	.	.
BG1*VN-TH34	PX984288	.	.	.	.	.	.	.	.	.	.	.	.	.	.	.	.	.	.	.	T	.	.	.	.	.	.	.	.	.	.	.	.	.	.
BG1*VN-TH35	PX984289	.	.	C	A	.	.	.	.	.	A	.	.	.	A	A	.	.	.	.	.	.	.	.	.	.	.	.	.	.	.	.	.	.	.
BG1*VN-TH36	PX984290	G	.	C	A	.	.	.	.	.	A	.	.	.	.	.	.	.	.	.	.	.	.	.	.	.	.	.	.	.	.	G	.	.	.
BG1*VN-TH37	PX984291	.	.	C	.	A	T	.	.	.	.	.	.	.	.	.	.	.	.	.	.	.	.	.	.	.	.	.	.	.	.	G	.	.	.
BG1*VN-TH38	PX984292	G	C	.	.	.	.	.	.	.	.	.	.	.	.	.	.	.	.	.	.	.	.	.	.	.	.	.	.	.	.	.	.	.	.
BG1*VN-TH39	PX984293	.	C	.	.	.	.	.	.	.	A	.	.	.	.	.	.	.	.	.	.	.	.	.	.	.	.	.	.	.	.	.	.	.	.
BG1*VN-TH40	PX984294	.	.	C	T	A	T	.	.	.	A	.	.	.	.	.	.	.	.	.	.	.	.	.	.	.	.	.	.	.	.	.	.	C	.
BG1*VN-TH41	PX984295	.	.	C	A	.	.	.	.	.	.	.	.	.	.	.	.	.	.	.	.	.	.	.	.	.	.	.	.	.	.	G	.	.	.
BG1*VN-TH42	PX984296	.	C	C	.	A	T	.	G	.	.	.	.	.	.	.	.	.	.	T	.	.	.	.	.	.	.	.	.	.	.	G	.	.	.
BG1*VN43	PX984297	.	.	.	T	A	.	.	.	.	.	.	.	.	.	.	.	.	.	.	.	.	.	.	.	.	.	.	.	.	.	G	.	.	.
BG1*VN-TH44	PX984298	.	C	C	.	A	T	.	.	.	A	.	.	.	.	.	.	.	.	T	.	.	.	.	.	.	.	.	.	.	.	G	.	.	.
BG1*VN-TH45	PX984299	.	.	C	A	.	.	.	.	.	A	.	.	.	.	.	.	.	.	.	.	.	.	.	.	.	.	.	.	.	.	G	.	.	.
BG1*VN-TH46	PX984300	.	.	C	A	.	.	.	.	.	.	.	.	.	.	.	.	.	.	.	.	.	.	.	.	.	.	.	.	.	.	G	.	.	.
BG1*VN47	PX984301	.	C	.	.	.	.	.	.	.	.	.	.	.	.	.	.	.	.	.	.	.	.	.	.	.	.	.	.	.	.	.	.	.	.
BG1*VN48	PX984302	G	.	C	T	A	T	.	.	.	A	.	.	.	.	.	.	.	.	.	.	.	.	.	.	.	.	.	.	.	.	G	.	.	.
BG1*VN-TH49	PX984303	G	.	C	.	.	T	.	.	T	.	A	.	.	.	.	.	.	.	.	.	.	.	.	.	.	.	.	.	G	.	G	.	.	.
BG1*VN50	PX984304	.	.	.	.	.	.	.	.	.	.	.	.	.	.	.	.	.	.	.	.	.	.	.	.	.	.	.	.	.	.	G	.	.	.
BG1*VN-TH51	PX984305	.	.	.	.	.	.	.	.	.	A	.	.	.	.	.	.	.	.	.	.	.	.	.	.	.	.	.	.	.	.	.	.	.	.
BG1*VN-TH52	PX984306	.	C	C	A	.	.	.	.	.	.	.	.	.	.	.	.	.	.	.	.	.	.	.	.	.	.	.	.	.	.	G	.	.	.
BG1*VN-TH53	PX984307	.	T	C	A	.	.	.	.	.	.	.	.	.	.	.	.	.	.	.	.	.	.	.	.	.	.	.	.	.	.	G	.	.	.
BG1*VN54	PX984308	.	C	C	G	.	.	.	.	.	.	.	.	.	.	.	.	.	.	.	.	.	.	.	.	.	.	.	.	.	.	G	.	.	.
BG1*VN-TH55	PX984309	.	.	C	A	.	.	.	.	.	.	.	.	.	.	.	T	.	.	.	.	.	.	.	.	.	.	.	.	.	.	G	.	C	.
BG1*VN-TH56	PX984310	.	.	C	.	.	.	.	.	.	A	.	.	.	.	.	.	.	.	.	.	.	.	.	.	.	.	.	.	.	.	G	.	.	.
BG1*VN-TH57	PX984311	.	.	C	.	A	T	.	.	.	A	.	.	.	.	.	.	.	.	.	.	.	T	.	.	.	.	.	.	.	.	G	.	.	.
BG1*VN-TH58	PX984312	.	.	C	A	.	.	.	.	.	.	.	.	.	.	.	.	.	.	.	.	.	.	.	.	.	.	.	.	.	.	G	.	.	.
BG1*VN-TH59	PX984313	.	T	.	.	.	.	.	.	.	.	.	.	.	.	.	.	.	.	.	.	.	.	.	.	.	.	.	.	.	.	.	.	.	.
BG1*VN-TH60	PX984314	.	.	C	T	A	T	.	.	.	.	.	.	.	.	.	.	.	.	.	.	.	.	.	.	.	.	.	.	.	.	.	.	.	.
BG1*TH61	PX984315	G	.	.	T	A	.	.	.	.	A	.	.	.	.	.	.	.	A	.	.	.	.	.	.	.	.	.	.	.	.	G	.	.	.
BG1*TH62	PX984316	.	.	.	G	.	.	.	.	.	A	.	.	.	.	.	.	.	.	T	.	.	.	.	.	.	.	.	.	.	.	G	.	.	.
BG1*TH63	PX984317	.	.	C	A	.	.	.	.	.	A	.	.	.	.	.	.	.	.	.	.	.	.	.	.	.	.	.	.	.	.	G	.	.	.
BG1*TH64	PX984318	G	.	C	.	.	T	.	.	.	A	.	.	.	.	.	.	.	.	.	.	.	.	.	.	.	.	.	.	.	.	G	.	.	.
BG1*TH65	PX984319	.	.	C	.	A	T	.	.	.	.	.	.	.	.	.	.	.	.	.	.	.	.	.	.	.	.	.	.	.	.	G	.	.	.
BG1*TH66	PX984320	.	.	C	T	A	T	.	.	.	.	.	.	.	.	.	.	.	.	.	.	.	.	.	.	.	.	.	.	.	.	G	A	.	.
BG1*TH67	PX984321	G	.	C	T	A	T	.	.	.	A	.	.	.	.	.	T	.	.	.	.	.	.	.	.	.	.	.	.	.	.	G	.	.	.
BG1*TH68	PX984322	G	.	C	.	.	T	.	.	.	A	.	.	.	.	.	.	.	.	.	.	.	.	.	.	.	.	.	.	.	.	G	.	.	.
BG1*TH69	PX984323	G	.	G	G	.	.	.	.	.	A	.	.	.	.	.	.	A	.	.	A	.	.	.	.	.	.	.	.	.	.	G	.	.	.
BG1*TH70	PX984324	G	.	C	T	A	T	.	.	.	.	.	.	.	.	.	.	.	.	.	.	.	.	.	.	.	.	.	.	.	.	.	.	.	.
BG1*TH71	PX984325	.	.	C	.	A	T	.	.	.	.	.	.	.	.	.	.	.	.	.	.	.	.	.	.	.	.	.	.	.	.	G	.	.	.
BG1*TH72	PX984326	.	.	C	A	.	.	.	.	.	.	.	.	.	.	.	.	.	.	.	.	.	.	.	.	.	.	.	.	.	.	G	.	.	.
BG1*TH73	PX984327	.	.	C	T	A	T	.	.	.	A	.	.	.	.	.	.	.	.	.	.	.	.	.	.	.	A	.	.	.	.	G	.	.	.
BG1*TH74	PX984328	.	.	C	A	.	.	.	.	.	A	.	.	C	.	.	.	.	.	.	.	.	.	.	.	.	.	.	.	.	.	G	.	.	.
BG1*TH75	PX984329	.	C	C	T	A	T	.	.	.	.	.	.	.	.	.	.	.	.	T	.	.	.	.	.	.	.	.	.	.	.	G	.	.	.
BG1*TH76	PX984330	.	.	C	.	A	T	.	G	.	.	.	.	.	.	.	.	.	.	T	.	.	.	.	.	.	.	.	.	.	.	G	.	.	.
BG1*TH77	PX984331	.	C	C	A	.	.	G	.	.	A	.	.	.	.	.	.	.	.	.	.	.	.	.	.	.	.	.	.	.	.	G	.	.	.
BG1*TH78	PX984332	.	.	C	A	.	.	.	.	.	.	.	.	.	.	.	.	.	.	T	.	.	.	.	.	.	.	.	.	.	.	G	.	C	.
BG1*TH79	PX984333	.	T	C	A	.	.	.	.	T	.	.	.	.	.	.	.	.	.	.	.	.	.	.	.	.	.	.	.	.	.	G	.	.	.
BG1*TH80	PX984334	.	.	C	T	A	T	.	.	.	A	.	.	.	.	.	.	.	.	.	.	.	.	.	.	.	.	.	.	.	C	G	.	C	.
BG1*TH81	PX984335	.	.	C	.	A	T	.	.	.	.	.	.	.	.	.	T	.	.	.	.	.	.	.	.	.	.	.	.	.	.	.	.	.	.
BG1*TH82	PX984336	.	.	C	.	.	.	.	.	.	.	.	.	.	.	.	.	.	.	.	.	.	.	.	.	.	.	.	.	.	.	G	.	.	.
BG1*TH83	PX984337	.	.	C	T	A	T	.	.	.	.	.	.	.	.	.	.	.	.	.	A	.	.	.	.	.	.	.	.	.	.	G	.	.	.
BG1*TH84	PX984338	.	.	C	A	.	.	.	.	.	.	.	.	.	.	.	.	.	.	.	A	.	.	.	.	.	.	.	.	.	.	G	.	.	.
BG1*TH85	PX984339	.	.	C	G	.	.	.	.	.	.	.	.	.	.	.	.	.	.	.	.	.	.	.	.	.	.	.	.	.	.	G	.	C	.
BG1*TH86	PX984340	G	.	.	.	A	.	.	.	.	A	.	.	.	.	.	.	.	.	.	.	.	.	.	T	.	.	.	.	.	.	G	.	.	.
BG1*TH87	PX984341	.	.	G	G	.	.	.	.	.	A	.	.	.	.	.	.	A	.	.	A	.	.	.	.	.	.	.	.	.	.	G	.	.	.
BG1*TH88	PX984342	.	C	C	T	A	T	.	.	.	.	.	.	.	.	.	.	.	.	T	.	.	.	.	.	.	.	.	.	.	.	G	.	.	.
BG1*TH89	PX984343	.	.	C	.	.	T	.	.	T	.	A	.	.	.	.	.	.	.	.	.	.	.	.	.	.	.	.	T	.	.	G	.	.	.
BG1*TH90	PX984344	.	.	C	.	.	.	.	.		A	.	.	.	.	.	.	.	.	.	.	.	.	.	.	.	.	.	.	.	.	C	.	.	.
BG1*TH91	PX984345	.	.	C	G	.	.	G	.	.	.	.	.	.	.	.	.	.	.	.	.	.	.	.	.	.	.	.	.	.	.	G	.	C	.
BG1*TH92	PX984346	.	.	C	.	A	T	.	.	.	.	.	.	.	.	.	.	.	.	.	.	.	.	.	.	.	.	.	.	.	.	G	.	.	.
BG1*TH93	PX984347	G	.	C	A	.	.	.	.	.	.	.	.	.	.	.	.	.	.	.	.	.	.	.	.	.	.	.	.	.	.	G	.	.	.
BG1*TH94	PX984348	.	.	C	.	.	.	.	.	.	.	.	.	.	.	.	.	.	.	.	.	.	.	.	.	.	.	.	.	.	.	G	.	.	.
BG1*TH95	PX984349	.	.	C	.	.	T	.	.	.	.	.	.	.	.	.	.	.	.	.	.	.	.	.	.	.	.	.	.	.	.	G	.	.	.
BG1*TH96	PX984350	G	.	C	T	A	.	.	.	.	A	.	.	.	.	.	.	.	.	.	.	.	.	.	.	.	.	.	.	.	C	G	.	.	.
BG1*TH97	PX984351	.	T	.	.	.	.	.	.	.	A	.	.	.	.	.	.	.	.	.	.	.	.	T	.	.	.	.	.	.	.	G	.	C	.
BG1*TH98	PX984352	.	.	C	.	A	T	.	.	.	.	.	.	.	.	.	.	.	.	T	.	.	.	.	.	.	.	.	.	.	.	G	.	.	.

* Nucleotide position based on reference sequence (accession number OM953775).

**Table 2:** Diversity of nucleotide sequences of the *BG1* gene.

Breed/Red Junglefowl Subspecies	Population	*N * ^1^	*N*_a _^2^	*π * ^3^
**Vietnam**				
Ac	Tra Vinh	20	14	0.055
	Tien Giang	20	17	0.059
	Long An	20	19	0.057
Noi	Dong Thap	15	17	0.058
	Vinh Long	15	12	0.055
	Ben Tre	7	9	0.052
Tre	Can Tho	5	8	0.054
	Tra Vinh	13	17	0.044
	An Giang1	8	9	0.042
	An Giang2	5	4	0.033
Hmong	Hung Yen	15	12	0.055
Dong Tao	Hung Yen	11	9	0.045
Tau Vang	Ca Mau	15	13	0.050
**Indigenous and local chicken**		169	58	0.056
*G. gallus. Spadiceus*	Kien Giang	10	11	0.051
*G. gallus gallus*	An Giang	3	5	0.050
**Red junglefowl**		13	13	0.051
Total		182	60	0.056
**Thailand**				
Betong	Lopburi	15	16	0.060
Nin Kaset (Black)	Lopburi	10	20	0.056
Nin Kaset (White)	Lopburi	10	15	0.054
Phuphan Black	Sakon Nakhon (Black)	7	15	0.052
Chee Fah	Chiang Rai	10	18	0.054
Chee	Phitsanulok	10	12	0.047
Decoy	Phitsanulok, Sukhothai, Chiang Mai	6	12	0.057
Dong Tao	Udon Thani	5	10	0.053
	Lopburi	10	16	0.052
Mixing-fighting cock	Bangken	10	14	0.055
Lao Pa Koi	Lamphun	10	17	0.054
Khaew Paree	Phitsanulok	10	16	0.056
Lueng Hang Khao	Phitsanulok Panyanukun School	8	16	0.054
Mae Hong Son	Chiang Mai	10	19	0.054
	Mae Hong Son Farmer	10	23	0.050
	MRLBC	20	19	0.057
Pradu Hang Dam	Phitsanulok 2	6	16	0.058
	Chiang Mai	6	4	0.058
	Nakhon Prathom	5	3	0.057
Samae Dam	Uthai 1	7	2	0.002
	Uthai 2	4	13	0.056
Wein Chang	Udon Thani	10	9	0.055
**Indigenous and local chicken**		199	85	0.056
*G. gallus spadiceus*	Huai Sai	10	6	0.051
	Khao Kho	10	25	0.057
	Songkhla	6	15	0.050
*G. gallus gallus*	Songkhla	10	10	0.056
	Chiang Mai	9	19	0.055
	Chanthaburi	10	10	0.060
	Roi Et	10	7	0.062
	Sa Kaew	10	12	0.053
	SiSaket	10	10	0.051
	Huai Sai	4	5	0.051
**Red junglefowl**		89	44	0.057
Total		288	89	0.057

^1^ number of samples (*N*); ^2^ number of maximum alleles per populations (*N*_a_); ^3^ nucleotide diversity (*π*).

**Table 3:** Rates of synonymous (*dS*) and nonsynonymous (*dN*) substitutions in the nucleotide sequences of the *BG1* gene.

Breed/Red Junglefowl Subspecies	Population	*dN*	*dS*	*ω* (*dN*/*dS*)	*Z*-Test		LRT	*p*-Value
					*Z*-Score	*p*-Value		
**Vietnam**								
Ac	Tra Vinh	0.010	0.034	0.294	−0.958	0.340	0.000	0.500
	Tien Giang	0.017	0.031	0.548	−0.613	0.541	0.000	0.500
	Long An	0.010	0.034	0.294	−0.760	0.449	0.325	0.425
Noi	Dong Thap	0.019	0.028	0.679	−0.348	0.729	0.191	0.455
	Vinh Long	0.014	0.038	0.368	−0.861	0.391	0.352	0.419
	Ben Tre	0.010	0.021	0.476	−0.474	0.636	2.184	0.168
Tre	Can Tho	0.007	0.035	0.200	−0.953	0.343	0.168	0.459
	Tra Vinh	0.014	0.021	0.667	−0.292	0.771	0.090	0.478
	An Giang1	0.010	0.021	0.476	−0.441	0.660	6.203	0.022
	An Giang2	0.010	0.018	0.556	−0.367	0.714	−0.0001	0.500
Hmong	Hung Yen	0.019	0.033	0.576	−0.483	0.630	0.000	0.500
Dong Tao	Hung Yen	0.008	0.033	0.242	−0.896	0.372	0.000	0.500
Tau Vang	Ca Mau	0.015	0.036	0.417	−0.941	0.349	0.000	0.500
**Indigenous and local chicken**		0.015	0.032	0.469	−0.667	0.506	0.000	0.500
*G. gallus spadiceus*	Kien Giang	0.008	0.019	0.421	−0.485	0.628	2.186	0.168
*G. gallus gallus*	An Giang	0.008	0.019	0.421	−1.133	0.259	0.329	0.424
**Red junglefowl**		0.011	0.027	0.407	−0.747	0.457	0.000	0.500
Total		0.014	0.032	0.438	−0.693	0.489	0.000	0.500
**Thailand**								
Betong	Lopburi	0.017	0.036	0.472	−0.678	0.499	0.000	0.500
Nin Kaset (Black)	Lopburi	0.012	0.036	0.333	−0.908	0.365	2.332	0.156
Nin Kaset (White)	Lopburi	0.011	0.037	0.297	−0.999	0.320	0.065	0.484
**Phuphan Black**	Sakon Nakhon (Black)	0.018	0.051	0.353	−1.169	0.245	0.000	0.500
Chee Fah	Chiang Rai	0.021	0.05	0.420	−0.876	0.383	0.000	0.500
Chee	Phitsanulok	0.013	0.04	0.325	−0.971	0.334	0.000	0.500
Decoy	Phitsanulok, Sukhothai, Chiang Mai	0.020	0.041	0.488	−0.674	0.501	0.000	0.500
Dong Tao	Udon Thani	0.019	0.014	1.357	0.232	0.817	0.000	0.500
	Lopburi	0.016	0.027	0.593	−0.409	0.683	0.112	0.473
Mixing-fighting cock	Bangken	0.006	0.048	0.125	−1.290	0.200	0.000	0.500
Lao Pa Koi	Lamphun	0.015	0.043	0.349	−0.968	0.335	0.000	0.500
Khaew Paree	Phitsanulok	0.015	0.031	0.484	−0.548	0.585	0.000	0.500
Lueng Hang Khao	Phitsanulok Panyanukun School	0.006	0.042	0.143	−1.386	0.168	0.000	0.500
Mae Hong Son	Chiang Mai	0.018	0.037	0.486	−0.707	0.481	0.000	0.500
	Mae Hong Son Farmer	0.017	0.029	0.586	−0.497	0.620	0.000	0.500
	MRLBC	0.012	0.04	0.300	−0.934	0.352	0.000	0.500
Pradu Hang Dam	Phitsanulok 2	0.013	0.035	0.371	−0.896	0.372	0.388	0.412
	Chiang Mai	0.017	0.033	0.515	−0.687	0.493	0.235	0.445
	Nakhon Prathom	0.000	0.053	0.000	−1.283	0.202	0.229	0.446
Samae Dam	Uthai 1	0.000	0.000	0.000	0.000	1.000	-	-
	Uthai 2	0.030	0.044	0.682	−0.514	0.608	0.000	0.500
Wein Chang	Udon Thani	0.010	0.023	0.435	−0.649	0.518	0.000	0.500
**Indigenous and local chicken**		0.015	0.039	0.385	−0.840	0.403	0.000	0.500
*G. gallus spadiceus*	Huai Sai	0.016	0.039	0.410	−0.931	0.354	0.267	0.438
	Khao Kho	0.015	0.033	0.455	−0.612	0.541	0.000	0.500
	Songkhla	0.013	0.031	0.419	−0.660	0.510	0.000	0.500
*G. gallus gallus*	Songkhla	0.017	0.024	0.708	−0.258	0.797	0.000	0.500
	Chiang Mai	0.013	0.045	0.289	−0.976	0.331	0.000	0.500
	Chanthaburi	0.002	0.044	0.045	−1.475	0.143	1.167	0.279
	Roi Et	0.017	0.026	0.654	−0.409	0.683	0.000	0.500
	Sa Kaew	0.013	0.022	0.591	−0.380	0.705	0.000	0.500
	SiSaket	0.012	0.019	0.632	−0.301	0.764	0.000	0.500
	Huai Sai	0.017	0.049	0.347	−0.689	0.492	0.000	0.500
**Red junglefowl**		0.013	0.035	0.371	−0.796	0.427	0.000	0.500
Total		0.015	0.038	0.395	−0.817	0.416	0.000	0.500

LRT: Likelihood Ratio Test.

**Table 4:** Neutrality test for the *BG1* gene sequences.

Breed/Red Junglefowl Subspecies	Population	Tajima’s *D*	Fu and Li’s *F*	Fu and Li’s *D*
**Vietnam**				
Ac	Tra Vinh	0.201	0.407	0.417
	Tien Giang	−0.452	−0.497	−0.405
	Long An	−0.587	−0.164	0.107
Noi	Dong Thap	−0.707	−0.559	−0.374
	Vinh Long	−0.330	−0.559	−0.374
	Ben Tre	−0.018	0.425	0.510
Tre	Can Tho	−0.450	−0.164	−0.049
	Tra Vinh	−0.475	−0.432	−0.317
	An Giang1	−0.375	−0.359	−0.287
	An Giang2	−0.095	−0.163	0.162
Hmong	Hung Yen	0.020	−0.025	−0.039
Dong Tao	Hung Yen	−0.513	−0.818	−0.784
Tau Vang	Ca Mau	−0.120	0.414	0.572
**Indigenous and local** **chicken**		−0.717	−0.324	0.172
*G. gallus spadiceus*	Kien Giang	0.020	0.168	0.197
*G. gallus gallus*	An Giang	−0.260	−0.149	−0.107
**Red junglefowl**		−0.079	−0.121	−0.115
Total		−0.682	1.263	3.406
**Thailand**				
Betong	Lopburi	0.324	1.405	1.739
Nin Kaset (Black)	Lopburi	−0.238	1.416	2.041
Nin Kaset (White)	Lopburi	−0.235	0.687	1.017
**Phuphan Black**	Sakon Nakhon (Black)	−0.508	0.023	0.279
Chee Fah	Chiang Rai	−0.703	−0.498	−0.283
Chee	Phitsanulok	0.309	1.408	1.618
Decoy	Phitsanulok, Sukhothai, Chiang Mai	−0.450	−0.164	−0.019
Dong Tao	Udon Thani	−0.447	−0.070	0.116
	Lopburi	−0.264	0.307	0.494
Mixing-fighting cock	Bangken	−0.106	0.177	0.289
Lao Pa Koi	Lamphun	−0.317	0.477	0.808
Khaew Paree	Phitsanulok	−0.827	−0.423	−0.133
Lueng Hang Khao	Phitsanulok Panyanukun School	−1.009	−1.666	−1.608
Mae Hong Son	Chiang Mai	−0.163	0.985	1.384
	Mae Hong Son Farmer	−0.185	0.352	0.626
	MRLBC	−0.502	−0.103	0.130
Pradu Hang Dam	Phitsanulok 2	−1.015	−0.783	−0.527
	Chiang Mai	−0.968	−0.832	−0.607
	Nakhon Prathom	0.171	0.370	0.385
Samae Dam	Uthai 1	1.342	1.102	0.953
	Uthai 2	−0.413	−0.048	0.125
Wein Chang	Udon Thani	0.407	0.212	0.076
**Indigenous and local** **chicken**		−0.802	1.249	3.361
*G. gallus spadiceus*	Huai Sai	−0.007	0.311	0.368
	Khao Kho	−0.969	−1.282	−1.139
	Songkhla	−0.382	−0.269	−0.158
*G. gallus gallus*	Songkhla	−0.716	−0.898	−0.817
	Chiang Mai	−0.623	−0.727	−0.614
	Chanthaburi	0.517	1.377	1.476
	Roi Et	0.703	1.147	1.103
	Sa Kaew	0.332	0.983	1.073
	SiSaket	0.359	0.566	0.539
	Huai Sai	−0.206	−0.154	−0.120
**Red junglefowl**		−0.217	1.279	2.242
Total		−0.707	1.304	3.447

Statistical significance evaluated at q < 0.05 following Benjamini–Hochberg FDR correction. No significant deviations were observed.

## Data Availability

All genotyping data and detailed calculation results generated during this study are available in “The Siam Chicken Bioresource Project, SCBP” (https://www.sci.ku.ac.th/scbp/, accessed on 30 January 2025 and Dryad dataset: https://datadryad.org/stash/share/x2qlPmboMgCROXO8, accessed on 15 January 2026). The sequences have been deposited in the NCBI GenBank database (https://www.ncbi.nlm.nih.gov/) under accession numbers PX984255–PX984352.

## Supplementary Materials

The following supporting information can be downloaded at: https://www.mdpi.com/article/10.3390/ani16091398/s1, Figure S1. Bayesian phylogenetic tree for *BG1* gene identification and target region selection for polymorphic analysis. The values above the branches represent posterior probability; Figure S2. *BG1* protein secondary structure production of indigenous chicken populations in Thailand and Vietnam; Figure S3. Genetic differentiation among populations was evaluated using Principal Coordinate Analysis (PCoA) based on allele frequency data; Figure S4. Alignment of amino acid sequences of *BG1* gene alleles from partial exon 16 with their 3D structures of signal sequences and Ig-like domain retrieved from UniProt database; Figure S5. Alignment of amino acid sequences of partial exon 16 of the *BG1* gene alleles with the reference sequences from Uniprot (B5BSR2) and Korean native chicken (Black: WBF70102); Table S1. Detailed information on specimens from 47 populations of indigenous and local chicken breeds and red jungle fowl from Thailand and Vietnam; Table S2. Information on the individual chicken breeds used for gene identification and target region selection for polymorphic analysis; Table S3. Distribution and specificity of *BG1* alleles in indigenous and local chickens and red junglefowl populations from Thailand and Vietnam; Table S4. Distribution of *BG1* gene alleles in Vietnamese indigenous and local chicken breeds and red junglefowl; Table S5. Distribution of *BG1* gene alleles in indigenous and local chicken breeds from Thailand and in red junglefowl; Table S6 Mutation types and their locations in partial fragments of the *BG1* gene exon 4; Table S7. Results of analysis of variance (AMOVA) for indigenous and local chicken populations and red junglefowl from Thailand and Vietnam; Table S8. Detailed site-by-site results from the mixed effects model of evolution (MEME) analysis based on alleles of the *BG1* gene; Table S9. Detailed site-by-site results from fixed effects likelihood (FEL) analysis based on alleles of the *BG1* gene; Table S10. Detailed site-by-site results from fast unconstrained Bayesian approximation (FUBAR) analysis based on alleles of the *BG1* gene.

## Abbreviations

The following abbreviations are used in this manuscript:

Abbreviation	Definition
3D	Tertiary
AMOVA	Analysis of molecular variance
*AR*	Allelic richness
BIC	Bayesian information criterion
*dN*	The average rates of nonsynonymous
DNA	Deoxyribonucleic acid
DOC	Degree of Change
*dS*	The average rates of synonymous
EDTA	Ethylenediaminetetraacetic acid
FEL	Fixed effects likelihood
FUBAR	Fast unconstrained Bayesian approximation
ITIM	Immunoreceptor Tyrosine-based Inhibition Motif
iTOL	Interactive Tree of Life
LRT	Likelihood ratio test
MCMC	Markov Chain Monte Carlo
MDV	Marek’s disease virus
MEME	Mixed effects model of evolution
MHC	Major histocompatibility complex
*N*_a_	Number of alleles
NGS	Next-generation sequencing
PCoA	Principal coordinate analysis
PCR	Polymerase chain reaction
RJF	Red Junglefowl
SCBP	Siam Chicken Bioresource Project
SNPs	Single Nucleotide Polymorphisms
WGS	Whole-genome sequencing

## References

[1] Lawler A. (2014). Why Did the Chicken Cross the World?.

[2] Peters J., Lebrasseur O., Best J., Miller H., Fothergill T., Dobney K., Thomas R.M., Maltby M., Sykes N., Hanotte O. (2015). Questioning new answers regarding Holocene chicken domestication in China. Proc. Natl. Acad. Sci. USA.

[3] Gueye E.F. (2009). The role of networks in information dissemination to family poultry farmers. World’s Poult. Sci. J..

[4] Budi T., Singchat W., Tanglertpaibul N., Thong T., Panthum T., Chaiyes A., Muangmai N., Sawatdichaikul O., Griffin D.K., Duengkae P. (2025). Allelic diversity of *Blec2* gene in indigenous and local chickens and red junglefowl in Thailand: Implications for disease resistance. Vet. Anim. Sci..

[5] Nguyen Van D., Moula N., Moyse E., Do Duc L., Vu Dinh T., Farnir F. (2020). Productive performance and egg and meat quality of two indigenous poultry breeds in Vietnam, Ho and Dong Tao, fed on commercial feed. Animals.

[6] Desvaux S., Le Thuy N., Quang D.V., Vu Chi C. (2008). Vietnam: Characterization and Safeguarding of Genetic Resources in Agriculture.

[7] Mekchay S., Supakankul P., Assawamakin A., Wilantho A., Chareanchim W., Tongsima S. (2014). Population structure of four Thai indigenous chicken breeds. BMC Genet..

[8] Hunt H.D., Goto R.M., Foster D.N., Bacon L.D., Miller M.M. (2006). At least one YMHCI molecule in the chicken is alloimmunogenic and dynamically expressed on spleen cells during development. Immunogenetics.

[9] Manjula P., Fulton J.E., Seo D., Lee J.H. (2020). Major histocompatibility complex B variability in Korean native chicken breeds. Poult. Sci..

[10] Szeto C., Lobos C.A., Nguyen A.T., Gras S. (2020). TCR recognition of peptide–MHC-I: Rule makers and breakers. Int. J. Mol. Sci..

[11] Miller M.M., Goto R., Young S., Liu J., Hardy J. (1990). Antigens similar to major histocompatibility complex B-G are expressed in the intestinal epithelium in the chicken. Immunogenetics.

[12] Goto R.M., Wang Y., Taylor R.L., Wakenell P.S., Hosomichi K., Shiina T., Blackmore C.S., Briles W.E., Miller M.M. (2009). BG1 has a major role in MHC-linked resistance to malignant lymphoma in the chicken. Proc. Natl. Acad. Sci. USA.

[13] Supikamolseni A., Ngaoburanawit N., Sumontha M., Chanhome L., Suntrarachun S., Peyachoknagul S., Srikulnath K. (2015). Molecular barcoding of venomous snakes and species-specific multiplex PCR assay to identify snake groups for which antivenom is available in Thailand. Genet. Mol. Res..

[14] Eimes J.A., Reed K.M., Mendoza K.M., Bollmer J.L., Whittingham L.A., Bateson Z.W., Dunn P.O. (2013). Greater prairie chickens have a compact MHC-B with a single class IA locus. Immunogenetics.

[15] Andrews S. (2010). FastQC: A Quality Control Tool for High-Throughput Sequence Data. Babraham Bioinformatics. https://www.bioinformatics.babraham.ac.uk/projects/fastqc/.

[16] Sebastian A., Herdegen M., Migalska M., Radwan J. (2016). Amplisas: A web server for multilocus genotyping using next-generation amplicon sequencing data. Mol. Ecol. Resour..

[17] He K., Liang C.H., Zhu Y., Dunn P., Zhao A., Minias P. (2022). Reconstructing macroevolutionary patterns in avian MHC architecture with genomic data. Front. Genet..

[18] Lighten J., Van Oosterhout C., Bentzen P. (2014). Critical review of NGS analyses for de novo genotyping multigene families. Mol. Ecol..

[19] Ronquist F., Huelsenbeck J.P. (2003). MrBayes 3: Bayesian phylogenetic inference under mixed models. Bioinformatics.

[20] Kalyaanamoorthy S., Minh B.Q., Wong T.K.F., von Haeseler A., Jermiin L.S. (2017). ModelFinder: Fast model selection for accurate phylogenetic estimates. Nat. Methods.

[21] Letunic I., Bork P. (2021). Interactive Tree of Life (iTOL) v5: An online tool for phylogenetic tree display and annotation. Nucleic Acids. Res..

[22] R Core Team (2025). R: A Language and Environment for Statistical Computing.

[23] Bandelt H.J., Forster P., Röhl A. (1999). Median-joining networks for inferring intraspecific phylogenies. Mol. Biol. Evol..

[24] Rozas J., Ferrer-Mata A., Sánchez-DelBarrio J.C., Guirao-Rico S., Librado P., Ramos-Onsins S.E., Sánchez-Gracia A. (2017). DnaSP 6: DNA sequence polymorphism analysis of large data sets. Mol. Biol. Evol..

[25] Kamvar Z.N., Tabima J.F., Grünwald N.J. (2014). Poppr: An R package for genetic analysis of populations with clonal, partially clonal, and/or sexual reproduction. PeerJ.

[26] Murrell B., Weaver S., Smith M.D., Wertheim J.O., Murrell S., Aylward A., Eren K., Pollner T., Martin D.P., Smith D.M. (2015). Gene-wide identification of episodic selection. Mol. Biol. Evol..

[27] Murrell B., Wertheim J.O., Moola S., Weighill T., Scheffler K., Kosakovsky Pond S.L. (2012). Detecting individual sites subject to episodic diversifying selection. PLoS Genet..

[28] Murrell B., Moola S., Mabona A., Weighill T., Sheward D., Kosakovsky Pond S.L., Scheffler K. (2013). FUBAR: A fast, unconstrained Bayesian approximation for inferring selection. Mol. Biol. Evol..

[29] Abramson J., Adler J., Dunger J., Evans R., Green T., Pritzel A., Ronneberger O., Willmore L., Ballard A.J., Bambrick J. (2024). Accurate structure prediction of biomolecular interactions with AlphaFold 3. Nature.

[30] Laskowski R.A., MacArthur M.W., Moss D.S., Thornton J.M. (1993). PROCHECK: A program to check the stereochemical quality of protein structures. J. Appl. Crystallogr..

[31] Kaufman J. (2018). Generalists and specialists: A new view of how MHC class I molecules fight infectious pathogens. Trends Immunol..

[32] Fortier A.L., Pritchard J.K. (2025). Ancient trans-species polymorphism at the major histocompatibility complex in primates. eLife.

[33] Talarico L., Marta S., Rossi A.R., Crescenzo S., Petrosino G., Martinoli M., Tancioni L. (2021). Balancing selection, genetic drift, and human-mediated introgression interplay to shape MHC (functional) diversity in Mediterranean brown trout. Ecol. Evol..

[34] Meyer D., Thomson G. (2001). How selection shapes variation of the human major histocompatibility complex: A review. Ann. Hum. Genet..

[35] Li Z., Hassan M., Ahmad H.I., Ashraf M.A., Asif A.R., Qadeer I., Shahzad A.H., Abbas S., Sajid M., Mateen A. (2025). Structural and evolutionary constraints shape adaptive landscapes of immune-related genes across mammalian phylogeny. PLoS ONE.

[36] Schneider K., White T.J., Mitchell S., Adams C.E., Reeve R., Elmer K.R. (2021). The pitfalls and virtues of population genetic summary statistics: Detecting selective sweeps in recent divergences. J. Evol. Biol..

[37] Ekerette E., Tanglertpaibul N., Budi T., Auekingpetch W., Nguyen C.P.T., Singchat W., Wongloet W., Kumnan N., Chalermwong P., Luu A.H. (2025). Phuphan chicken breeds: Classification as varieties or distinct breeds with three derivative groups using microsatellite genotyping. Anim. Biosci..

[38] Al Mughram M.H., Catalano C., Herrington N.B., Safo M.K., Kellogg G.E. (2023). 3D interaction homology: The hydrophobic residues alanine, isoleucine, leucine, proline and valine play different structural roles in soluble and membrane proteins. Front. Mol. Biosci..

[39] Xu X., Masubuchi T., Cai Q., Zhao Y., Hui E. (2021). Molecular features underlying differential SHP1/SHP2 binding of immune checkpoint receptors. eLife.

[40] Luu A.H., Budi T., Singchat W., Nguyen C.P.T., Panthum T., Tanglertpaibul N., Thong T., Vangnai K., Chaiyes A., Yokthongwattana C. (2025). Comparison of unique Dong Tao chickens from Vietnam and Thailand: Genetic background and differences for resource management. Genes Genom..

[41] Mogano R.R., Mpofu T.J., Mtileni B., Hadebe K. (2025). South African indigenous chickens’ genetic diversity, and the adoption of ecological niche modelling and landscape genomics as strategic conservation techniques. Poult. Sci..

