# Supplementary material for: Ancestral BG1 Alleles and Structural Conservation Ensure Immune-Related Genetic Resilience in Southeast Asian Chicken Lineages
Source: Animals (Basel). 2026 May 3;16(9):1398. doi: 10.3390/ani16091398 (PMC13162802; doi:10.3390/ani16091398)
Supplement: Supplementary file 1 [file animals-16-01398-s001.zip › animals-4265464-supplementary.pdf]

Supplementary Figures

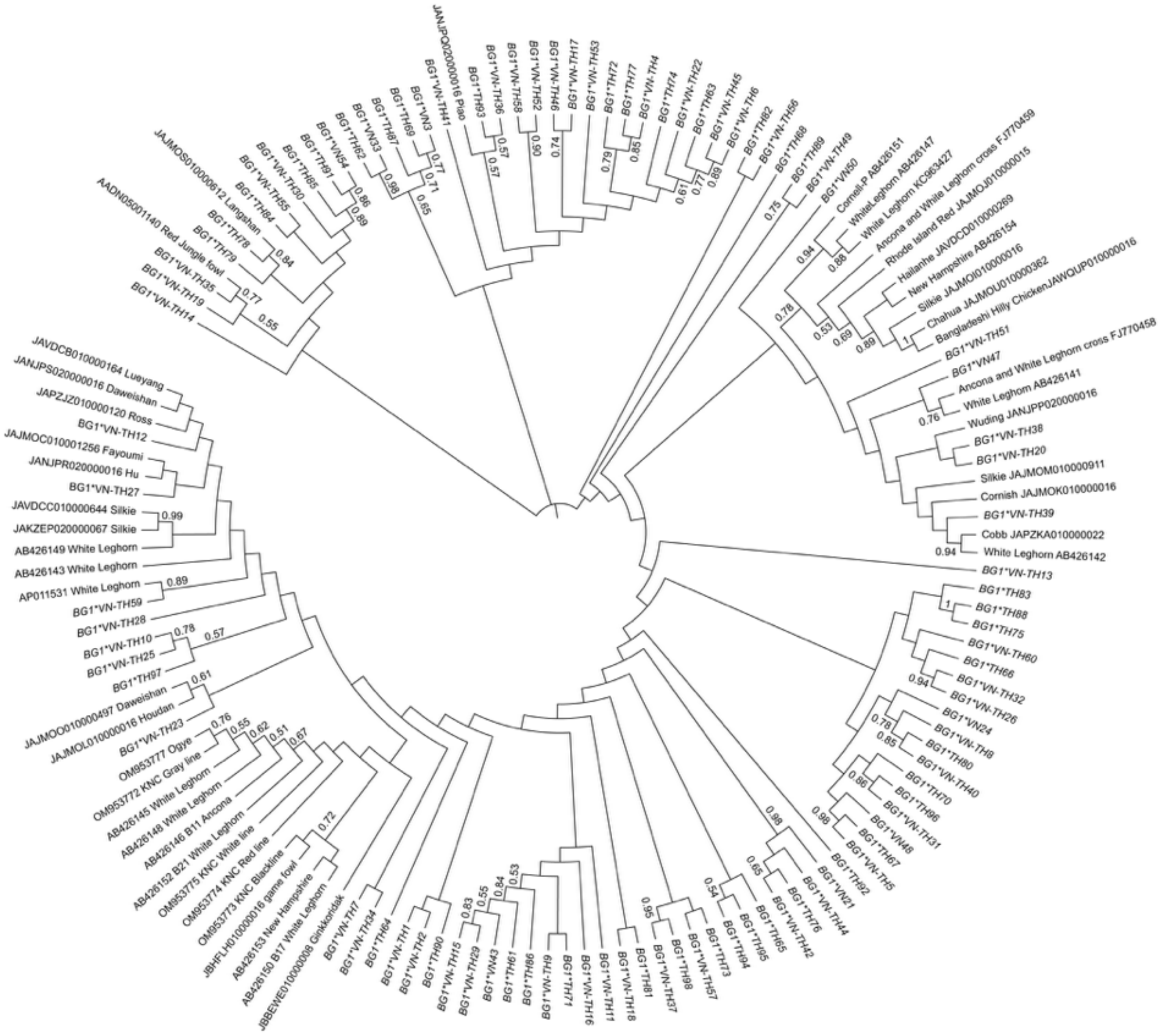

Figure S1. Bayesian phylogenetic tree for *BG1* gene identification and target region selection for polymorphic analysis. The values above the branches represent posterior probability.

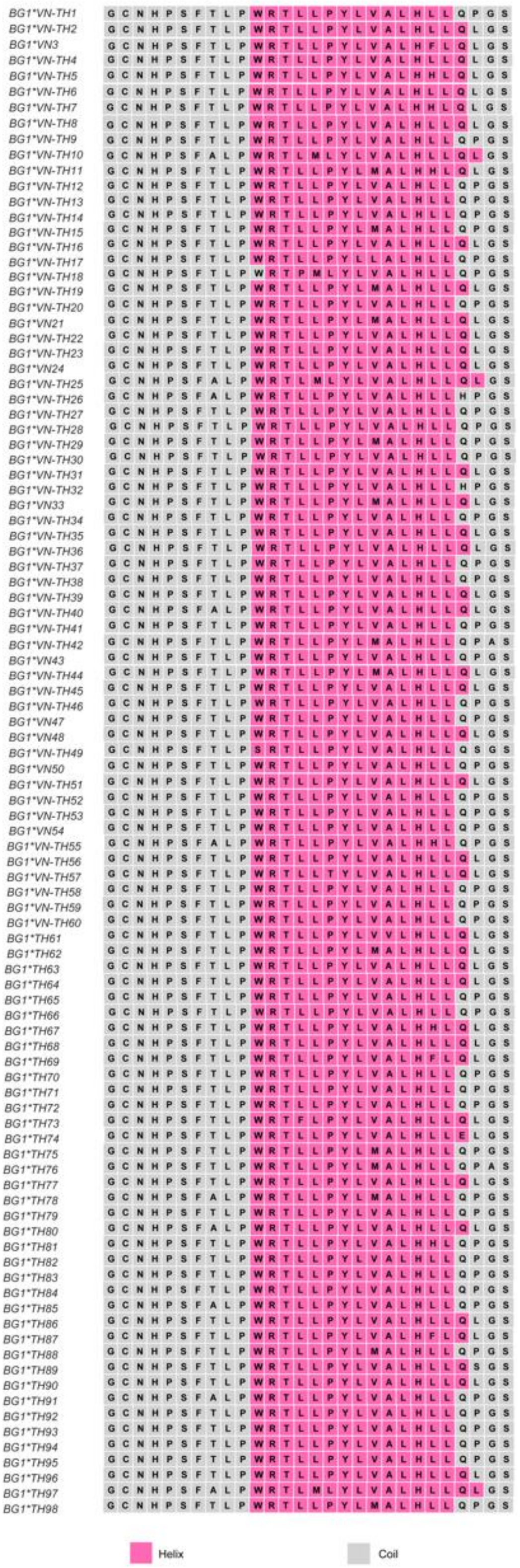

Figure S2. *BG1* protein secondary structure production of indigenous chicken populations in Thailand and Vietnam

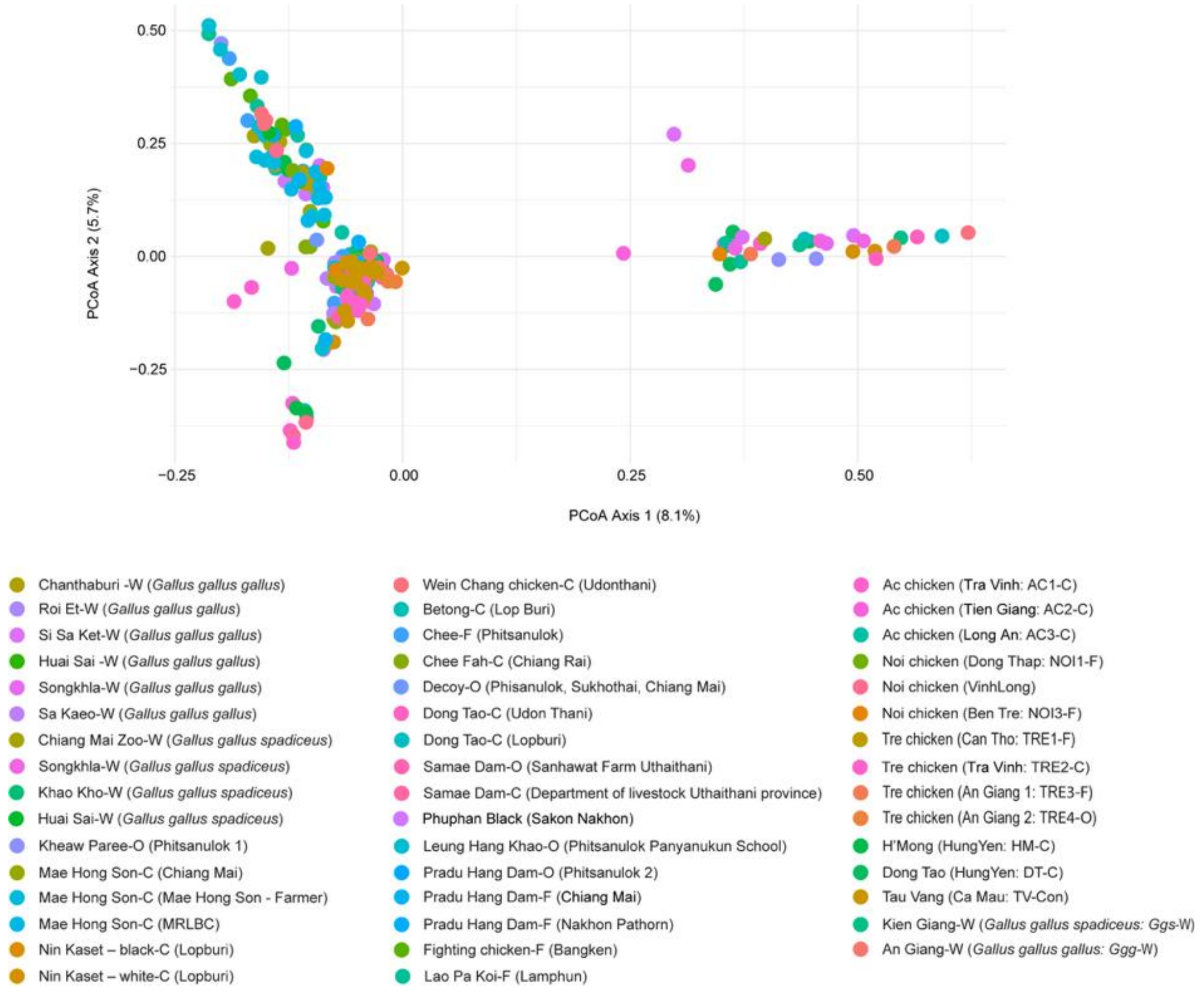

Figure S3. Genetic differentiation among populations was evaluated using Principal Coordinate Analysis (PCoA) based on allele frequency data

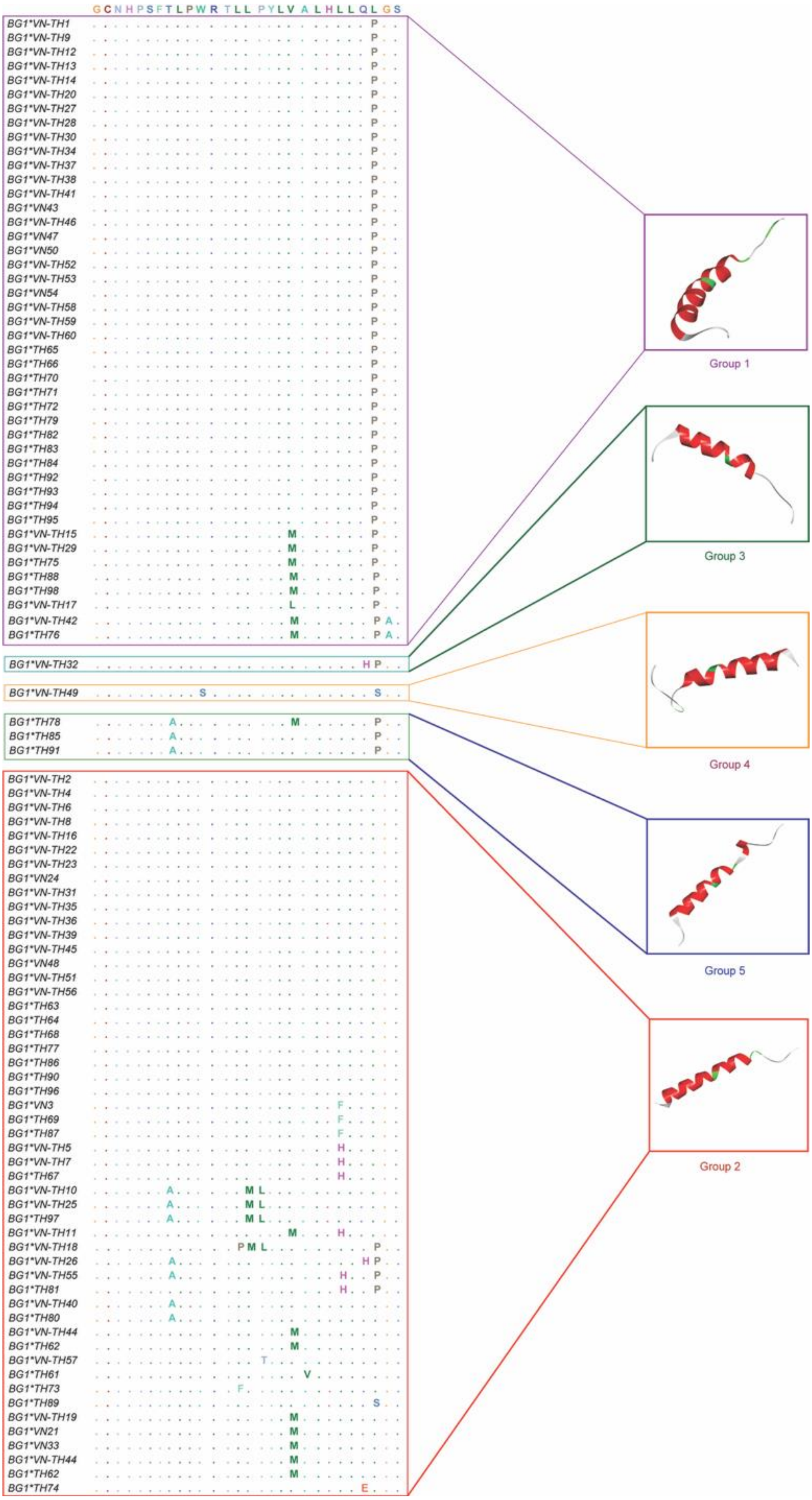

Figure S4. Alignment of amino acid sequences of *BG1* gene alleles from partial exon 16 with their 3D structures of signal sequences and *Ig*-like domain retrieved from UniProt database

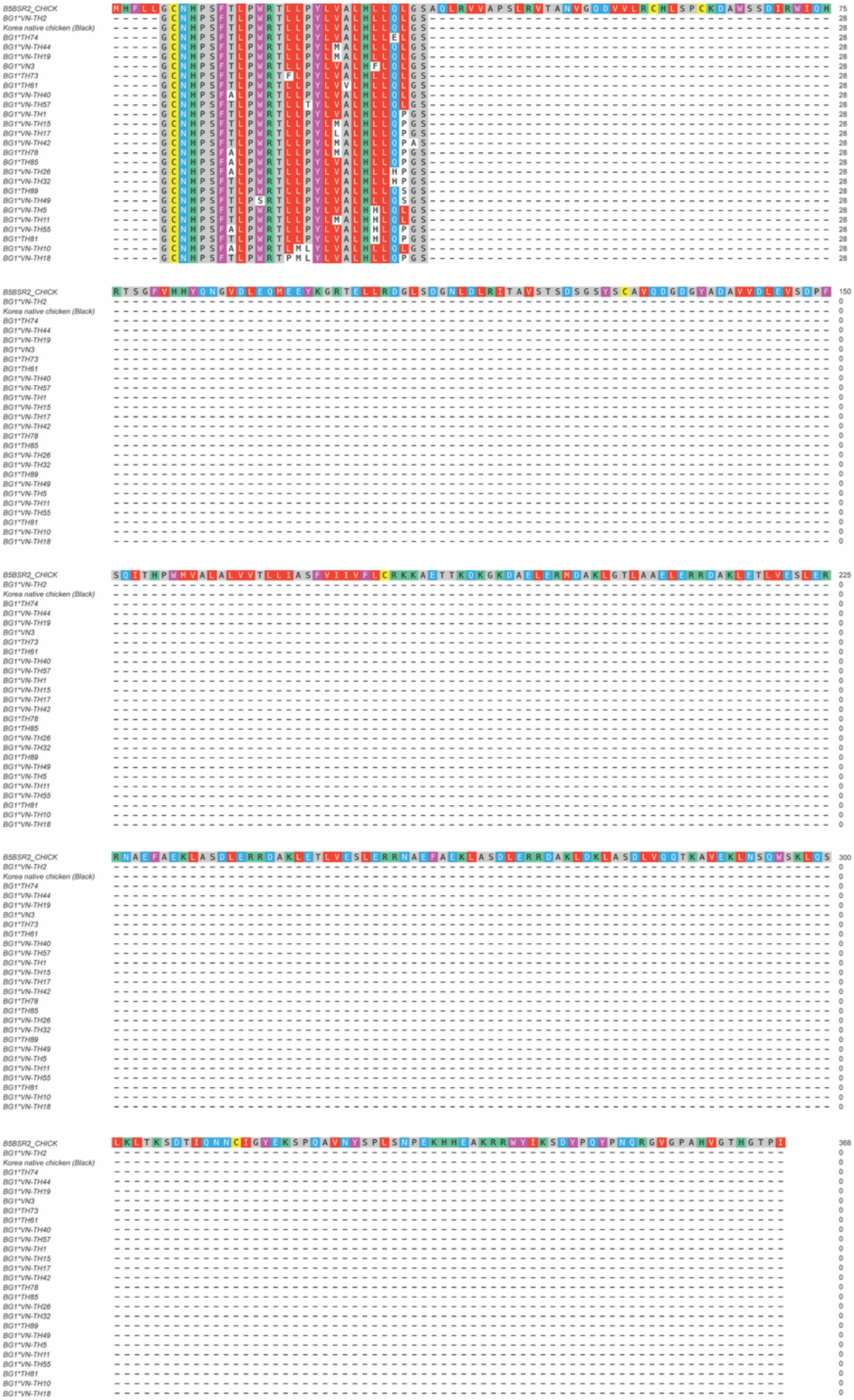

Figure S5. Alignment of amino acid sequences of partial exon 16 of the *BG1* gene alleles with the reference sequences from Uniprot (B5BSR2) and Korean native chicken (Black: WBF70102)

Supplementary Tables

Table S1. Detailed information on the specimens from 47 populations of indigenous and local chicken breeds, and red jungle fowl from Thailand and Vietnam

Breeds	Population	Purpose	Abbreviation	Sample (*N*)
**Vietnam**				
Ac	Tra Vinh	Consumption	AC1-C	20
	Tien Giang	Consumption	AC2-C	20
	Long An	Consumption	AC3-C	20
Noi	Dong Thap	Fighting	NOI1-F	15
	Vinh Long	Fighting	NOI2-F	15
	Ben Tre	Fighting	NOI3-F	7
Tre	Can Tho	Fighting	TRE1-F	5
	Tra Vinh	Consumption	TRE2-C	13
	An Giang1	Fighting	TRE3-F	8
	An Giang2	Ornamental	TRE4-O	5
Hmong	Hung Yen	Consumption	HM-C	15
Dong Tao	Hung Yen	Consumption	DT-C	11
Tau Vang	Ca Mau	Conservation	TV-Con	15
**Indigenous and local**				169
G. gallus spadiceus	Kien Giang	Wild	*Ggs*-W	10
G. gallus gallus	An Giang	Wild	*Ggg*-W	3
Red junglefowl				13
Total				182
**Thailand**				
Betong	Lopburi	Consumption	Betong-C	15
Nin Kaset (Black)	Lopburi	Consumption	BLBF-C	10
Nin Kaset (White)	Lopburi	Consumption	BLBW-C	10
Phuphan Black	Sakon Nakhon (Black)	Consumption	BLP-C	7
Chee Fah	Chiang Rai	Consumption	CF-C	10
Chee	Phitsanulok	Consumption	CH-C	10
Decoy	Phitsanulok, Sukhothai, Chiang Mai	Consumption	Dcy-C	6
Dong Tao	Udon Thani	Consumption	DT1-C	5
	Lopburi	Consumption	DT2-C	10
Mixing-fighting cock	Bangken	Fighting	FC-F	10
Lao Pa Koi	Lamphun	Fighting	Koi-F	10
Khaew Paree	Phitsanulok	Fighting	KP-F	10
Lueng Hang Khao	Phitsanulok Panyanukun School	Fighting	LHK3-F	8
Mae Hong Son	Chiang Mai	Consumption	MHS1-C	10
	Mae Hong Son Farmer	Consumption	MHS2-C	10
	MRLBC	Consumption	MHS3-C	20
Pradu Hang Dam	Phitsanulok 2	Fighting	PDH2-F	6

Breeds	Population	Purpose	Abbreviation	Sample (*N*)
	Chiang Mai	Fighting	PDH3-F	6
	Nakhon Prathom	Fighting	PDH4-F	5
Samae Dam	Uthai 1	Consumption	SD1-C	7
	Uthai 2	Consumption	SD2-C	4
Wein Chang	Udon Thani	Consumption	WZ-C	10
**Indigenous and local**				199
	Huai Sai	Wild	*Gsai*-W	10
G. gallus spadiceus	Khao Kho	Wild	*KK*-W	10
	Songkhla	Wild	*SSkl*-W	6
	Songkhla	Wild	*Gskl*-W	10
	Chiang Mai	Wild	*CMZ*-W	9
	Chanthaburi	Wild	*Gct*-W	10
G. gallus gallus	Roi Et	Wild	*Gre*-W	10
	Sa Kaew	Wild	*Gskw*-W	10
	SiSaket	Wild	*Gsskt*-W	10
	Huai Sai	Wild	Ssai-W	4
Red junglefowl				89
Total				288

Table S2. Information on the individual chicken breeds used for gene identification and target region selection for polymorphic analysis

Database	Breed or red junglefowl	Accession number	Locality
**National Center for Biotechnology Information (NCBI)**	Ancona and White Leghorn cross	FJ770459	USA
	Ancona and White Leghorn cross	FJ770458	USA
	Ancona	AB426146	Japan
	Bangladeshi Hilly Chicken	JAWQUP010000016	Bangladeshi
	Chahua	JAJMOU010000362	China
	Cobb	JAPZKA010000022	Spain
	Cornell	AB426151	Japan
	Cornish	JAJMOK010000016	China
	Daweishan	JAJMOO010000497	China
	Daweishan	JANJPS020000016	USA
	Fayoumi	JAJMOC010001256	China
	Xishuangbanna Game fowl	JBHFLH010000016	China
	Ginkkoridak	JBBEWE010000008	Korea
	Hailanhe	JAVDCD010000269	China
	Houdan	JAJMOL010000016	China
	Hu	JANJPR020000016	USA
	KNC_White line	OM953775	Korea
	KNC_Black line	OM953773	Korea
	KNC_Gray line	OM953772	Korea
	KNC_Red line	OM953774	Korea
	Langshan	JAJMOS010000612	China
	Lueyang	JAVDCB010000164	China
	New Hampshire	AB426154	Japan
	New Hampshire	AB426153	Japan
	Ogye	OM953777	Korea
	Piao	JANJPQ020000016	USA
	Red Junglefowl	AADN05001140	USA
	Rhode Island Red	JAJMOJ010000015	China
	Ross	JAPZJZ010000120	Spain
	Silkie	JAJMOI010000016	China
	Silkie	JAJMOM010000911	China
	Silkie	JAVDCC010000644	China
	Silkie	JAKZEP020000067	China

Database	Breed or red junglefowl	Accession number	Locality
	White Leghorn	KC963427	United Kingdom
	White Leghorn	AB426147	Japan
	White Leghorn	AB426141	Japan
	White Leghorn	AB426143	Japan
	White Leghorn	AB426152	Japan
	White Leghorn	AB426148	Japan
	White Leghorn	AB426145	Japan
	White Leghorn	AB426142	Japan
	White Leghorn	AB426150	Japan
	White Leghorn	AB426149	Japan
	White Leghorn	AP011531	Japan
	Wuding	JANJPP020000016	USA

Table S3. Distribution and specificity of *BG1* Alleles in indigenous and local chickens and red junglefowl populations from Thailand and Vietnam

Alleles	Vietnamese chicken				Thai chicken			
	Indigenous	RJF	Indigenous and RJF	Unique	Indigenous	RJF	Indigenous and RJF	Unique
*BG1*VN-TH1*	-	-	✓	-	-	-	✓	-
*BG1*VN-TH2*	-	-	✓	-	-	-	✓	-
*BG1*VN3*	✓	-	-	✓	-	-	-	-
*BG1*VN-TH4*	✓	-	-	-	✓	-	-	-
*BG1*VN-TH5*	✓	-	-	-	-	-	✓	-
*BG1*VN-TH6*	-	-	✓	-	-	-	✓	-
*BG1*VN-TH7*	✓	-	-	-	-	-	✓	-
*BG1*VN-TH8*	✓	-	-	-	-	-	✓	-
*BG1*VN-TH9*	✓	-	-	-	-	-	✓	-
*BG1*VN-TH10*	✓	-	-	-	-	-	✓	-
*BG1*VN-TH11*	✓	-	-	-	✓	-	-	-
*BG1*VN-TH12*	✓	-	-	-	-	-	✓	-
*BG1*VN-TH13*	✓	-	-	-	-	-	✓	-
*BG1*VN-TH14*	-	-	✓	-	-	-	✓	-
*BG1*VN-TH15*	✓	-	-	-	✓	-	-	-
*BG1*VN-TH16*	✓	-	-	-	✓	-	-	-
*BG1*VN-TH17*	✓	-	-	-	✓	-	-	-
*BG1*VN-TH18*	-	-	✓	-	✓	-	-	-
*BG1*VN-TH19*	✓	-	-	-	-	-	✓	-
*BG1*VN-TH20*	✓	-	-	-	-	-	✓	-
*BG1*VN21*	✓	-	-	✓	-	-	-	-
*BG1*VN-TH22*	-	-	✓	-	✓	-	-	-
*BG1*VN-TH23*	-	-	✓	-	✓	-	-	-
*BG1*VN24*	✓	-	-	✓			-	-
*BG1*VN-TH25*	✓	-	-	-	✓	-	-	-
*BG1*VN-TH26*	✓	-	-	-			✓	-
*BG1*VN-TH27*	-	-	✓	-	✓	-	-	-
*BG1*VN-TH28*	-	-	✓	-	-	-	✓	-
*BG1*VN-TH29*	✓	-	-	-	-	-	✓	-
*BG1*VN-TH30*	✓	-	-	-	✓	-	-	-
*BG1*VN-TH31*	✓	-	-	-	✓	-	-	-
*BG1*VN-TH32*	✓	-	-	-	-	-	✓	-
*BG1*VN33*	✓	-	-	✓	-	-	-	-
*BG1*VN-TH34*	✓	-	-	-	-	-	✓	-
*BG1*VN-TH35*	✓	-	-	-	✓	-	-	-
*BG1*VN-TH36*	✓	-	-	-			✓	-
*BG1*VN-TH37*	✓	-	-	-	-	✓	-	-
*BG1*VN-TH38*	✓	-	-	-	-	-	✓	-
*BG1*VN-TH39*	✓	-	-	-	-	-	✓	-
*BG1*VN-TH40*	✓	-	-	-	✓	-	-	-
*BG1*VN-TH41*	-	-	✓	-	✓	-	-	-
*BG1*VN-TH42*	✓	-	-	-	✓	-	-	-
*BG1*VN43*	✓	-	-	✓	-	-	-	-
*BG1*VN-TH44*	✓	-	-	-	-	-	✓	-
*BG1*VN-TH45*	✓	-	-	-	-	-	✓	-
*BG1*VN-TH46*	✓	-	-	-	-	-	✓	-
*BG1*VN47*	✓	-	-	✓	-	-	-	-
*BG1*VN48*	✓	-	-	✓	-	-	-	-

Alleles	Vietnamese chicken				Thai chicken			
	Indigenous	RJF	Indigenous and RJF	Unique	Indigenous	RJF	Indigenous and RJF	Unique
*BG1*VN-TH49*	✓	-	-	-	✓	-	-	-
*BG1*VN50*	✓	-	-	✓	-	-	-	-
*BG1*VN-TH51*	-	-	✓	-	-	-	✓	-
*BG1*VN-TH52*	-	✓	-	-	-	-	✓	-
*BG1*VN-TH53*	-	✓	-	-	-	-	✓	-
*BG1*VN54*	✓	-	-	✓	-	-	-	-
*BG1*VN-TH55*	✓	-	-	-	-	-	✓	-
*BG1*VN-TH56*	✓	-	-	-	-	✓	-	-
*BG1*VN-TH57*	✓	-	-	-	✓	-	-	-
*BG1*VN-TH58*	✓	-	-	-	-	-	✓	-
*BG1*VN-TH59*	✓	-	-	-	-	✓	-	-
*BG1*VN-TH60*	✓	-	-	-	✓	-	-	-
*BG1*TH61*	-	-	-	-	-	-	✓	✓
*BG1*TH62*	-	-	-	-	-	-	✓	✓
*BG1*TH63*	-	-	-	-	-	✓	-	✓
*BG1*TH64*	-	-	-	-	-	-	✓	✓
*BG1*TH65*	-	-	-	-	-	-	✓	✓
*BG1*TH66*	-	-	-	-	-	-	✓	✓
*BG1*TH67*	-	-	-	-	-	-	✓	✓
*BG1*TH68*	-	-	-	-	-	-	✓	✓
*BG1*TH69*	-	-	-	-	-	-	✓	✓
*BG1*TH70*	-	-	-	-	-	-	✓	✓
*BG1*TH71*	-	-	-	-	-	-	✓	✓
*BG1*TH72*	-	-	-	-	-	-	✓	✓
*BG1*TH73*	-	-	-	-	✓	-	-	✓
*BG1*TH74*	-	-	-	-	✓	-	-	✓
*BG1*TH75*	-	-	-	-	✓	-	-	✓
*BG1*TH76*	-	-	-	-	✓	-	-	✓
*BG1*TH77*	-	-	-	-	✓	-	-	✓
*BG1*TH78*	-	-	-	-	✓	-	-	✓
*BG1*TH79*	-	-	-	-	✓	-	-	✓
*BG1*TH80*	-	-	-	-	✓	-	-	✓
*BG1*TH81*	-	-	-	-	✓	-	-	✓
*BG1*TH82*	-	-	-	-	✓	-	-	✓
*BG1*TH83*	-	-	-	-	✓	-	-	✓
*BG1*TH84*	-	-	-	-	✓	-	-	✓
*BG1*TH85*	-	-	-	-	✓	-	-	✓
*BG1*TH86*	-	-	-	-	✓	-	-	✓
*BG1*TH87*	-	-	-	-	✓	-	-	✓
*BG1*TH88*	-	-	-	-	✓	-	-	✓
*BG1*TH89*	-	-	-	-	✓	-	-	✓
*BG1*TH90*	-	-	-	-	✓	-	-	✓
*BG1*TH91*	-	-	-	-	✓	-	-	✓
*BG1*TH92*	-	-	-	-	✓	-	-	✓
*BG1*TH93*	-	-	-	-	✓	-	-	✓
*BG1*TH94*	-	-	-	-	✓	-	-	✓
*BG1*TH95*	-	-	-	-	✓	-	-	✓
*BG1*TH96*	-	-	-	-	✓	-	-	✓
*BG1*TH97*	-	-	-	-	✓	-	-	✓
*BG1*TH98*	-	-	-	-	✓	-	-	✓

Table S4. Distribution of *BG1* gene alleles in Vietnamese indigenous and local chicken breeds and red junglefowl

Alleles	Ac Chicken			Noi chicken			Tre chicken							Red junglefowl		Indigenous and local breeds	Red junglefowl
	AC1	AC2	AC3	NOI1	NOI2	NOI3	TRE1	TRE2	TRE3	TRE4	HM	DT	TV	*Ggs*	*Ggg*		
*BG1*VN-TH1*	✓	✓	✓	✓	-	✓	-	-	✓	✓	-	✓	-	✓	✓	✓	✓
*BG1*VN-TH2*	✓	✓	✓	✓	-	✓	-	-	✓	-		✓	-	✓	✓	✓	✓
*BG1*VN3*	✓	✓	✓	-	✓	-	-	✓	-	-	✓	✓	-	-	-	✓	-
*BG1*VN-TH4*	✓	✓	✓	-		✓	✓	✓	-	-	✓	-	✓	-	-	✓	-
*BG1*VN-TH5*	✓	-	-	-	✓	-	-	-	-	-	-	-	✓	-	-	✓	-
*BG1*VN-TH6*	✓	✓	✓	-	-	-	-	-	-	-	-	-	-	✓	-	✓	✓
*BG1*VN-TH7*	✓	✓	-	-	-	-	-	-	-	-	-	✓	-	-	-	✓	-
*BG1*VN-TH8*	✓	-	-	-	✓	-	-	✓	✓	-	✓	✓	✓	-	-	✓	-
*BG1*VN-TH9*	✓	-	✓	-	-	✓	-	✓	-	-		✓	-	-	-	✓	-
*BG1*VN-TH10*	✓	-	-	✓	✓	-	-	-	-	-	✓	-	-	-	-	✓	-
*BG1*VN-TH11*	✓	✓	✓	-	-	-	-	-	-	-		✓	-	-	-	✓	-
*BG1*VN-TH12*	✓	✓	✓	-	-	-	-	-	-	-	-	-	-	-	-	✓	-
*BG1*VN-TH13*	✓	-	✓	-	-	-	-	-	-	-	-	-	-	-	-	✓	-
*BG1*VN-TH14*	✓	-	-	-	-	-	-	-	-	-	-	-	✓	✓	-	✓	✓
*BG1*VN-TH15*	-	✓	-	-	-	-	-	-	-	-	✓	-	-	-	-	✓	-
*BG1*VN-TH16*	-	✓	-	-	-	-	-	-	-	-	-	-	-	-	-	✓	-
*BG1*VN-TH17*	-	✓	-	-	-	-	-	-	-	-	-	-	-	-	-	✓	-
*BG1*VN-TH18*	-	✓	✓	-	-	-	-	-	-	-	-	-	-	-	✓	✓	✓
*BG1*VN-TH19*	-	✓	✓	-	-	-	✓	✓	✓	-	-	-	-	-	-	✓	-
*BG1*VN-TH20*	-	✓		✓	-	-	-	✓	✓	-	-	-	-	-	-	✓	-
*BG1*VN21*	-	✓	✓	-	-	-	-	-	-	-	-	-	-	-	-	✓	-
*BG1*VN-TH22*	-	✓	-	-	-	-	✓	-	-	-	-	✓		✓		✓	✓
*BG1*VN-TH23*	-	✓	-	✓	✓	-	-	✓	-	-	✓	-	✓		✓	✓	✓
*BG1*VN24*	-	-	✓	✓	-	-	-	-	-	-	-	-	-	-	-	✓	-
*BG1*VN-TH25*	-	-	✓	-	✓	-	-	-	-	-	✓	-	-	-	-	✓	-
*BG1*VN-TH26*	-	-	✓	-	-	-	-	-	-	-	-	-	-	-	-	✓	-
*BG1*VN-TH27*	-	-	✓	-	✓	✓		✓	✓	✓	✓	-	-	✓	✓	✓	✓
*BG1*VN-TH28*	-	-	✓	✓	✓	✓	✓	✓	-	-	-	-	-	✓	-	✓	✓
*BG1*VN-TH29*	-	-	✓	-	-	-	-	-	-	-	✓	-	-	-	-	✓	-
*BG1*VN-TH30*	-	-	✓	-	-	-	-	-	-	-	-	-	-	-	-	✓	-
*BG1*VN-TH31*	-	-	-	-	-	-	-	-	-	-	✓	-	-	-	-	✓	-
*BG1*VN-TH32*	-	-	-	-	-	-	-	-	-	-	✓	-	-	-	-	✓	-

Alleles	Ac Chicken			Noi chicken			Tre chicken							Red junglefowl		Indigenous and local breeds	Red junglefowl
	AC1	AC2	AC3	NOI1	NOI2	NOI3	TRE1	TRE2	TRE3	TRE4	HM	DT	TV	*Ggs*	*Ggg*		
*BG1*VN33*	-	-	-	✓	-	-	-	-	-	-	✓	-	-	-	-	✓	-
*BG1*VN-TH34*	-	-	-	-	-	-	-	-	-	-	-	✓	-	-	-	✓	-
*BG1*VN-TH35*	-	-	-	✓	-	-	-	-	-	-	-	-	-	-	-	✓	-
*BG1*VN-TH36*	-	-	-	✓	✓	-	-	-	-	-	-	-	-	-	-	✓	-
*BG1*VN-TH37*	-	-	-	✓	-	✓	-	-	-	-	-	-	-	-	-	✓	-
*BG1*VN-TH38*	-	-	-	✓	-	✓	-	-	-	-	-	-	✓	-	-	✓	-
*BG1*VN-TH39*	-	-	-	✓	✓	✓	-	✓	-	-	-	-	-	-	-	✓	-
*BG1*VN-TH40*	-	-	-	✓	-	-	✓	✓	-	-	-	-	-	-	-	✓	-
*BG1*VN-TH41*	-	-	-	✓	-	-	-	-	-	-	-	-	-	✓	-	✓	✓
*BG1*VN-TH42*	-	-	-	✓	-	-	-	-	-	-	-	-	-	-	-	✓	-
*BG1*VN43*	-	-	-	✓	✓	-	-	-	-	-	-	-	-	-	-	✓	-
*BG1*VN-TH44*	-	-	-	-	✓	-	-	-	-	-	-	-	✓	-	-	✓	-
*BG1*VN-TH45*	-	-	-	-	-	-	-	-	-	-	-	-	✓	-	-	✓	-
*BG1*VN-TH46*	-	-	-	-	-	-	-	-	-	-	-	-	✓	-	-	✓	-
*BG1*VN47*	-	-	-	-	-	-	✓	✓	-	-	-	-	✓	-	-	✓	-
*BG1*VN48*	-	-	-	-	-	-		✓	-	-	-	-	✓	-	-	✓	-
*BG1*VN-TH49*	-	-	-	-	-	-	-	-	-	-	-	-	✓	-	-	✓	-
*BG1*VN50*	-	-	-	-	-	-	-	-	-	-	-	-	✓	-	-	✓	-
*BG1*VN-TH51*	-	-	-	-	-	-	-	✓	-	-	-	-	-	✓	-	✓	✓
*BG1*VN-TH52*	-	-	-	-	-	-	-	-	-	-	-	-	-	✓	-	-	✓
*BG1*VN-TH53*	-	-	-	-	-	-	-	-	-	-	-	-	-	✓	-	-	✓
*BG1*VN54*	-	-	-	-	-	-	✓	-	-	-	-	-	-	-	-	✓	-
*BG1*VN-TH55*	-	-	-	-	-	-	✓	-	-	-	-	-	-	-	-	✓	-
*BG1*VN-TH56*	-	-	-	-	-	-	-	✓	✓	✓	-	-	-	-	-	✓	-
*BG1*VN-TH57*	-	-	-	-	-	-	-	✓	-	-	-	-	-	-	-	✓	-
*BG1*VN-TH58*	-	-	-	-	-	-	-	✓	✓	-	-	-	-	-	-	✓	-
*BG1*VN-TH59*	-	-	-	-	-	-	-	-	✓	-	-	-	-	-	-	✓	-
*BG1*VN-TH60*	-	-	-	-	-	-	-	-	-	✓	-	-	-	-	-	✓	-
*BG1*TH61 - BG1*TH98*	-	-	-	-	-	-	-	-	-	-	-	-	-	-	-	-	-

✓, allele observed; -, allele not observed

AC1: Ac chicken (Tra Vinh); AC2: Ac chicken (Tien Giang); AC3: Ac chicken (Long An); NOI1: Noi chicken (Dong Thap); NOI2: Noi chicken (Vinh Long); NOI3: Noi chicken (Ben Tre); TRE1: Tre chicken (Can Tho); TRE2: Tre chicken (Tra Vinh); TRE3: Tre chicken (An Giang 1); TRE4: Tre chicken (An Giang 2); HM: Hmong chicken (Hung Yen); DT: Dong Tao (Hung Yen); TV: Tau Vang (Ca Mau); *Ggs*: *G. gallus Spadiceus* (Kien Giang); *Ggg*: *G. gallus gallus* (An Giang).

Table S5. Distribution of *BG1* gene alleles in Thailand indigenous and local chicken breeds and red junglefowl

Alleles		Nin Kaset						Dong Tao							Mae Hong Son			Pradu Hang Dam			Samae Dam		*G. gallus spadiceus*					*G. gallus gallus*					Indigenous	RJF
	BT	NKB	NKW	BLP	CF	CH	Dcy	DT1	DT2	FC	Koi	KP	LHK3	MHS1	MHS2	MHS3	PDH2	PDH3	PDH4	SD1	SD2	WZ	Gsai	KK	SSkl	Gskl	CMZ	Gct	Gre	Gskw	Gsskt	Ssai		
*BG1*VN-TH1*	-	-	✓	-	-	-	-	✓	✓	-	✓	✓	-	-	-	-	-	-	-	-	-	✓	-	✓	✓	✓	✓	-	-	-	✓	-	✓	✓
*BG1*VN-TH2*	-	-	✓	-	-	-	-	✓	✓	-	✓	✓	-	-	-	-	-	-	-	-	-	✓	-	✓	✓	✓	✓	-	-	-	✓	-	✓	✓
*BG1*VN3*	-	-	-	-	-	-	-	-	-	-	-	-	-	-	-	-	-	-	-	-	-	-	-	-	-	-	-	-	-	-	-	-	-	-
*BG1*VN-TH4*	-	-	-	-	-	-	-	-	-	-	-	✓	-	✓	✓	✓	-	-	-	-	-	-	-	-	-	-	-	-	-	-	-	-	✓	-
*BG1*VN-TH5*	-	✓	✓	✓	✓	✓	-	-	✓	-	-	-	✓	✓	-	-	✓	-	-	-	✓	-	-	✓	✓	-	✓	-	-	-	-	-	✓	✓
*BG1*VN-TH6*	-	-	-	-	-	-	-	-	-	✓	-	-	-	-	-	-		-	-	-		-	-	✓	-	-		-	-	-	-	-	✓	✓
*BG1*VN-TH7*	-	✓	✓	✓	✓	-	-	-	-	-	-	-	✓	✓	-	✓	✓	-	-	-	✓	-	-	✓	-	-	✓	-	-	-	-	-	✓	✓
*BG1*VN-TH8*	-	✓	✓	✓	✓	-	-	-	-	-	-	-	✓	-	-	-	✓	-	-	-	✓	-	-	✓	✓	-	✓	-	-	✓	-	-	✓	✓
*BG1*VN-TH9*	-	-	-	-	✓	✓	-	-	-	✓	✓	✓	✓	✓	✓	✓	-	-	✓	-	-	-	✓	-	-	-		✓	-	✓	✓		✓	✓
*BG1*VN-TH10*	✓	✓		✓		✓	✓	-	-	✓	✓	✓	✓	✓	✓	✓	✓	✓	-	-	-	✓	✓		✓	-	✓	✓	-	-	-	✓	✓	✓
*BG1*VN-TH11*	-	-	-	-	-	✓	-	-	-	✓	✓	-	✓	-	-	✓	-	-	-	-	-	-	-	-	-	-	-	-	-	-	-	-	✓	-
*BG1*VN-TH12*	-	-	-	-	-	-	-	-	-	-	-	-	-	-	-	-	✓	-	-	-	-	-	-	-	✓	-	-	-	-	-	-	-	✓	✓
*BG1*VN-TH13*	-	✓	✓	-	-	-	-	-	-	-	-	-	-	-	-	-	-	-	-	-	✓	-	-	-	✓	-	-	-	-	-	-	-	✓	✓
*BG1*VN-TH14*	-			-	-	-	-	-	✓	-	-	✓	-	-	-	-	-	-	-	-	-	-	-	✓	-	-	-	-	-	-	-	-	✓	✓
*BG1*VN-TH15*	-	✓	✓	-	✓	-	-	-	-	-	-	-	-	-	✓	✓	-	-	-	-	-	-	-	-	-	-	-	-	-	-	-	-	✓	-
*BG1*VN-TH16*	-	-	-	✓	-	-	-	✓	-	-	-	-	-	-	-	✓	-	-	-	-	-	-	-	-	-	-	-	-	-	-	-	-	✓	-
*BG1*VN-TH17*	-	-	-	-	-	-	-	-	-	-	✓	-	-	-	✓	✓	-	-	-	-	-	-	-	-	-	-	-	-	-	-	-	-	✓	-
*BG1*VN-TH18*	-	-	-	-	-	-	-	-	-	-	✓	-	-	-	-	-	-	-	-	-	-	-	-	-	-	-	-	-	-	-	-	-	✓	-
*BG1*VN-TH19*	-	-	-	-	-	✓	-	-	✓	-	✓	-	-	-	-	-	-	-	-	-	-	-	-	✓	✓	✓	-	-	-	✓	-	-	✓	✓
*BG1*VN-TH20*	-	✓	-	-	✓	-	-	-	-	✓	-	-	-	-	-	✓	-	-	-	-	-	-	-	-	✓	✓	-	✓	-	✓	✓	-	✓	✓
*BG1*VN21*	-	-	-	-	-	-	-	-	-	-	-	-	-	-	-	-	-	-	-	-	-	-	-	-	-	-	-	-	-	-	-	-	-	-
*BG1*VN-TH22*	-	-	-	-	-	-	-	-	-	-	✓	-	-	-	-	-	-	-	-	-	-	✓	-	-	-	-	-	-	-	-	-	-	✓	-
*BG1*VN-TH23*	-	✓	✓	-	-	✓	-	-	-	✓	-	✓	-	-	-	-	-	-	-	-	-	-	-	-	-	-	-	-	-	-	-	-	✓	-
*BG1*VN24*	-	-	-	-	-	-	-	-	-	-	-	-	-	-	-	-	-	-	-	-	-	-	-	-	-	-	-	-	-	-	-	-	-	-
*BG1*VN-TH25*	-	✓	✓	✓	✓	-	-	-	-	-	-	-	-	-	-	-	✓	-	-	-	✓	-	-	-	-	-	-	-	-	-	-	-	✓	-
*BG1*VN-TH26*	-	-	-	-	✓	-	-	-	-	✓	-	-	-	-	-	-	-	-	-	-	-	-	-	✓	-	-	-	-	✓	-	-	✓	✓	✓
*BG1*VN-TH27*	-	-	✓	✓	✓	-	✓	-	✓	-	-	-	-	-	✓	✓	-	-	-	-	-	-	-	-	-	-		-		-		-	✓	-
*BG1*VN-TH28*	-	✓	-	✓	-	-	✓	✓	✓	-	✓	✓	-	✓	✓	-	-	-	-	-	✓	-	-	✓	✓	-	✓	-	-	✓	-	-	✓	✓

Alleles		Nin Kaset						Dong Tao							Mae Hong Son			Pradu Hang Dam		Samae Dam			G. gallus spadiceus					G. gallus gallus					Indigenous	RJF
	BT	NKB	NKW	BLP	CF	CH	Dcy	DT1	DT2	FC	Koi	KP	LHK3	MHS1	MHS2	MHS3	PDH2	PDH3	PDH4	SD1	SD2	WZ	Gsai	KK	SSkl	Gskl	CMZ	Gct	Gre	Gskw	Gsskt	Ssai		
*BG1*VN-TH29*	-	✓	-	✓	-	-	-	-	-	-	-	-	-	-	-	-	-	-	-	-	-	-	-	-	-	-	-	✓	-	-	-	-	✓	✓
*BG1*VN-TH30*	-	-	-	-	-	-	-	-	-	✓	-	✓	✓	-	-	-	-	-	✓	-	-	-	-	-	-	-	-	-	-	-	-	-	✓	-
*BG1*VN-TH31*	✓	-	-	-	-	-	-	-	-	-	-	-	-	✓	✓	-	-	-	-	-	-	✓	-	-	-	-	-	-	-	-	-	-	✓	-
*BG1*VN-TH32*	-	-	-	-	✓	-	-	✓	-	✓	-	-	-	-	-	-	-	-	-	-	-	-	✓	✓	-	-	-	-	-	-	-	-	✓	✓
*BG1*VN33*	-	-	-	-	-	-	-	-	-	-	-	-	-	-	-	-	-	-	-	-	-	-	-	-	-	-	-	-	-	-	-	-	-	-
*BG1*VN-TH34*	-	-	✓	-	-	-	-	✓	✓	-	✓	✓	-	-	-	-	-	-	-	-	-	-	-	✓	✓	✓	✓	-	-	-	✓	-	✓	✓
*BG1*VN-TH35*	✓	-	-	-	-	-	-	-	-	-	-	-	-	-	-	-	-	-	-	-	-	-	-	-	-	-	-	-	-	-	-	-	✓	
*BG1*VN-TH36*	-	✓	-	-	-	-	-	-	-	✓	-	-	-	-	-	-	-	-	-	-	-	-	✓	-	-	✓	✓	-	-	-	-	-	✓	✓
*BG1*VN-TH37*	-	-	-	-	-	-	-	-	-	-	-	-	-	-	-	-	-	-	-	-	-	-	-	-	-	-	-	-	✓	-	-	-	-	✓
*BG1*VN-TH38*	✓	-	-	-	-	✓	-	-	-	-	-	-	-	✓	✓	-	-	-	-	-	-	-	-	✓	-	-	-	-	✓	✓	-	-	✓	✓
*BG1*VN-TH39*	✓	-	-	-	-	-	-	-	✓	-	-	-	-	-	-	-	-	-	-	-	-	-	✓	-	✓	-	✓	-	-	-	-	✓	✓	✓
*BG1*VN-TH40*	-	✓	-	✓	-	-	-	-	-	-	-	-	-	-	-	-	-	-	-	-	-	-	-	-	-	-	-	-	-	-	-	-	✓	-
*BG1*VN-TH41*	-	-	-	-	-	-	✓	-	-	-	-	-	-	-	✓	✓	-	-	-	-	-	-	-	-	-	-	-	-	-	-	-	-	✓	-
*BG1*VN-TH42*	-	-	-	-	-	-	-	-	✓	✓	✓	✓	✓	-	-	-	-	-	-	-	✓	-	-	-	-	-	-	-	-	-	-	-	✓	-
*BG1*VN43*	-	-	-	-	-	-	-	-	-	-	-	-	-	-	-	-	-	-	-	-	-	-	-	-	-	-	-	-	-	-	-	-	-	-
*BG1*VN-TH44*	-	-	-	-	-	-	✓	-	✓	-	-	-	-	✓	✓	✓	-	-	-	-	-	-	-	✓	-	-	-	-	-	✓	-	-	✓	✓
*BG1*VN-TH45*	✓	-	-	-	-	-	-	-	-	-	-	-	-	-	-	-	-	-	-	-	-	-	-	-	-	-	-	-	✓	✓	✓	-	✓	✓
*BG1*VN-TH46*	✓	-	-	-	-	-	-	-	-	-	-	-	-	-	-	-	-	-	-	-	-	-	-	-	-	-	-	-	✓	✓	-	-	✓	✓
*BG1*VN47*	-	-	-	-	-	-	-	-	-	-	-	-	-	-	-	-	-	-	-	-	-	-	-	-	-	-	-	-	-	-	-	-	-	-
*BG1*VN48*	-	-	-	-	-	-	-	-	-	-	-	-	-	-	-	-	-	-	-	-	-	-	-	-	-	-	-	-	-	-	-	-	-	-
*BG1*VN-TH49*	-	-	-	✓	-	-	-	-	-	-	-	-	-	-	-	-	-	-	-	-	-	-	-	-	-	-	-	-	-	-	-	-	✓	-
*BG1*VN50*	-	-	-	-	-	-	-	-	-	-	-	-	-	-	-	-	-	-	-	-	-	-	-	-	-	-	-	-	-	-	-	-	-	-
*BG1*VN-TH51*	-	✓	✓	-	-	✓	✓	-	✓		✓	✓	✓	✓	✓	-	✓	✓	-	-	-	✓	-	✓	-	✓	✓	-	-	-	✓	-	✓	✓
*BG1*VN-TH52*	-	-	-	-	-	-	-	-	-	✓	-	-	-	-	-	-	✓	✓	-	-	-	✓	-	-	-	-	-	✓	-	-	✓	-	✓	✓
*BG1*VN-TH53*	-	-	-	-	-	-	✓	-	-	-	-	-	✓	-	-	-	-	-	-	-	-	-	✓	✓	-	-	✓	✓	-	-	-	-	✓	✓
*BG1*VN54*	-	-	-	-	-	-	-	-	-	-	-	-	-	-	-	-	-	-	-	-	-	-	-	-	-	-	-	-	-	-	-	-	-	-
*BG1*VN-TH55*	✓	-	-	-	-	-	-	-	-	✓	-	-	-	-	-	-	-	-	-	-	-	-	-	✓	-	-	-	-	-	-	-	-	✓	✓
*BG1*VN-TH56*	-	-	-	-	-	-	-	-	-	-	-	-	-	-	-	-	-	-	-	-	-	-	-	-	-	-	-	-	-	✓	-	-	-	✓
*BG1*VN-TH57*	-	✓	-	-	-	-	-	-	✓	-	-	-	-	-	-	-	-	-	-	-	-	-	-	-	-	-	-	-	-	-	-	-	✓	-

Alleles		Nin Kaset						Dong Tao							Mae Hong Son			Pradu Hang Dam			Samae Dam		*G. gallus spadiceus*						*G. gallus gallus*				
	BT	NKB	NKW	BLP	CF	CH	Dcy	DT1	DT2	FC	Koi	KP	LHK3	MHS1	MHS2	MHS3	PDH2	PDH3	PDH4	SD1	SD2	WZ	Gsai	KK	SSkl	Gskl	CMZ	Gct	Gre	Gskw	Gsskt	Ssai	Indigenous	RJF
*BG1*VN-TH58*	-	-	-	-	-	-	-	-	-	-	-	-	-	-	-	✓	-	-	-	-	-	-	-	✓	-	-	-	-	-	-	-	-	✓	✓
*BG1*VN-TH59*	-	-	-	-	-	-	-	-	-	-	-	-	-	-	-	-	-	-	-	-	-	-	-	-	✓	-	✓	-	-	-	-	-	-	✓
*BG1*VN-TH60*	✓	-	-	-	✓	✓	-	-	✓	-	-	-	-	-	-	✓	-	-	-	-	-	-	-	-	-	-	-	-	-	-	-	-	✓	-
*BG1*TH61*	✓	-	-	✓	✓	✓	-	-	-	-	-	-	-	-	-	-	-	-	-	-	-	-	-	-	-	-	-	✓	✓	-	-	-	✓	✓
*BG1*TH62*	✓	✓		✓		✓	-	-	-	✓	✓	✓	✓	-	✓	✓	✓	-	-	-	-	✓	-	-	✓	-	✓	✓	-	-	-	✓	✓	✓
*BG1*TH63*	-	-	-	-	-	-	-	-	-	-	-	-	-	-	-	-	-	-	-	-	-	-	-	-	-	-	-	✓	-	-	✓	-	-	✓
*BG1*TH64*	-	-	-	-	-	-	-	-	-	-	-	✓	✓	-	-	-	✓	-	-	-	-	-	-	✓	-	-	-	✓	-	-	-	-	✓	✓
*BG1*TH65*	✓	-	-	-	-	-	-	-	-	-	-	-	-	-	-	-	-	-	-	-	-	-	-	✓		✓	-	-	✓	✓	-	-	✓	✓
*BG1*TH66*	-	-	-	-	-	-	-	✓	-	-	✓	✓	-	-	-	-	-	-	-	-	-	-	-	✓	✓	✓	✓	-	-	-	✓	-	✓	✓
*BG1*TH67*	-	-	-	-	✓	-	-	-	-	-	-	-	-	-	-	-	-	-	-	-	-	-	-	✓	-	-	✓	-	-	-	-	✓	✓	✓
*BG1*TH68*	-	✓	✓	-	-	-	-	-	-	-	-	-	-	-	-	-	✓	-	-	-	-	-	-	✓	-	✓	✓	-	-	-	-	-	✓	✓
*BG1*TH69*	-	✓	✓	✓	✓	✓	✓	-	-	-	-	-	✓	✓	-	-	✓	-	-	-	✓	-	-	✓	-	-	✓	-	-	-	-	-	✓	✓
*BG1*TH70*	-	-	-	-	-	-	-	-	-	-	-	-	-	✓	✓	✓	-	-	-	-		-	-	✓	-	-	-	-	-	-	-	-	✓	✓
*BG1*TH71*	-	-	-	-	-	-	-	-	-	-	-	✓	-	✓	✓	✓	-	-	-	-	✓	-	-	-	-	-	✓	-	-	-	-	-	✓	✓
*BG1*TH72*	-	-	-	-	-	-	-	-	-	✓	-	-	✓	-	-	-	-	-	-	-	-	-	-	-	-	-	-	-	-	✓	-	-	✓	✓
*BG1*TH73*	-	-	-	-	-	-	-	-	-	-	-	✓	-	-	-	-	✓	-	-	-	-	-	-	-	-	-	-	-	-	-	-	-	✓	-
*BG1*TH74*	-	-	-	-	-	-	-	-	-	-	-	-	-	✓	✓	-	-	-	-	-	-	-	-	-	-	-	-	-	-	-	-	-	✓	-
*BG1*TH75*	-	-	-	-	-	-	-	-	-	-	-	-	-	✓	✓	-	-	-	-	-	-	-	-	-	-	-	-	-	-	-	-	-	✓	-
*BG1*TH76*	-	-	-	-	-	-	-	-	-	-	-	-	-	✓	✓	-	-	-	-	-	-	-	-	-	-	-	-	-	-	-	-	-	✓	-
*BG1*TH77*	✓	-	-	-	-	-	-	-	-	-	-	-	-	✓	✓	-	-	-	-	-	-	✓	-	-	-	-	-	-	-	-	-	-	✓	-
*BG1*TH78*	-	-	-	-	-	-	-	✓	-	-	-	-	-	✓	✓	-	-	-	-	-	-	-	-	-	-	-	-	-	-	-	-	-	✓	-
*BG1*TH79*	-	-	-	-	-	-	-	-	-	-	-	-	✓	✓	✓	-	-	-	-	-	-	-	-	-	-	-	-	-	-	-	-	-	✓	-
*BG1*TH80*	-	-	-	✓	-	-	✓	-	-	-	-	-	-	✓	✓	✓	-	-	-	-	-	-	-	-	-	-	-	-	-	-	-	-	✓	-
*BG1*TH81*	-	-	-	-	-	-	✓	-	-	-	✓	-	-	-	✓	✓	-	-	-	-	-	-	-	-	-	-	-	-	-	-	-	-	✓	-
*BG1*TH82*	-	-	-	-	-	-	-	✓	-	-	-	-	-	-	✓	-	-	-	-	-	-	-	-	-	-	-	-	-	-	-	-	-	✓	-
*BG1*TH83*	-	-	-	-	-	-	-	-	-	-	-	-	-	-	-	✓	-	-	-	-	-	-	-	-	-	-	-	-	-	-	-	-	✓	-
*BG1*TH84*	-	✓	✓	-	✓	-	-	-	-	-	-	-	-	-	-	-	✓	-	-	-	-	-	-	-	-	-	-	-	-	-	-	-	✓	-
*BG1*TH85*	-	✓		-		-	-	-	✓	-	-	-	-	-	-	-	-	-	-	-	-	-	-	-	-	-	-	-	-	-	-	-	✓	-
*BG1*TH86*	✓	-	-	-		-	-	-	-	-	-	-	-	-	-	-	-	-	-	-	-	-	-	-	-	-	-	-	-	-	-	-	✓	-

Alleles		Nin Kaset						Dong Tao						Mae Hong Son			Pradu Hang Dam			Samae Dam			*G. gallus spadiceus*			*G. gallus gallus*							Indigenous	RJF
	BT	NKB	NKW	BLP	CF	CH	Dcy	DT1	DT2	FC	Koi	KP	LHK3	MHS1	MHS2	MHS3	PDH2	PDH3	PDH4	SD1	SD2	WZ	Gsai	KK	SSkl	Gskl	CMZ	Gct	Gre	Gskw	Gsskt	Ssai		
*BG1*TH87*	✓	-	-	-	✓	✓	-	-	✓	-	-	-	-	-	-	-	-	-	-	-	-	-	-	-	-	-	-	-	-	-	-	-	✓	-
*BG1*TH88*	✓	-	-	-		-	-	-	-	-	-	-	-	-	-	-	-	-	-	-	-	-	-	-	-	-	-	-	-	-	-	-	✓	-
*BG1*TH89*	-	-	-	-	✓	-	-	-	-	-	-	-	-	-	-	-	-	-	-	-	✓	-	-	-	-	-	-	-	-	-	-	-	✓	-
*BG1*TH90*	-	-	-	-	✓	-	-	-	-	-	-	-	-	-	-	-	-	-	-	-	✓	-	-	-	-	-	-	-	-	-	-	-	✓	-
*BG1*TH91*	-	-	-	-		-	✓	-	-	-	-	-	-	-	-	-	-	-	-	-	-	-	-	-	-	-	-	-	-	-	-	-	✓	-
*BG1*TH92*	-	-	-	-	-	-	✓	-	-	-	-	-	✓	-	-	-	✓	✓	-	-	-	-	-	-	-	-	-	-	-	-	-	-	✓	-
*BG1*TH93*	-	-	-	-	-	-	-	✓	-	-	-	-	-	-	-	-	-	-	-	-	-	-	-	-	-	-	-	-	-	-	-	-	✓	-
*BG1*TH94*	-	-	-	-	-	-	-	-	-	-	-	-	-	-	-	-	-	-	-	✓	✓	-	-	-	-	-	-	-	-	-	-	-	✓	-
*BG1*TH95*	-	-	-	-	-	-	-	-	-	-	-	-	-	-	-	-	-	-	-	✓	✓	-	-	-	-	-	-	-	-	-	-	-	✓	-
*BG1*TH96*	-	-	-	-	-	-	-	-	-	-	-	-	-	-	-	-	✓	-	-	-	-	-	-	-	-	-	-	-	-	-	-	-	✓	-
*BG1*TH97*	-	-	-	-	-	-	-	-	-	-	-	-	-	-	-	-	-	-	✓	-	-	-	-	-	-	-	-	-	-	-	-	-	✓	-
*BG1*TH98*	-	-	-	-	-	-	-	-	-	-	✓	-	-	-	-	-	-	-	-	-	-	-	-	-	-	-	-	-	-	-	-	-	✓	-

✓, allele observed; -, allele not observed

BT: Betong chicken (Lopburi); BLBF: Nin Kaset Black (Lopburi); BLBW: Nin Kaset White (Lopburi); BLP: Phuphan Black (Sakon Nakhon); CF: Chee Fah (Chiang Rai); CH: Chee (Phitsanulok); Dcy: Decoy (Phitsanulok, Sukhothai, Chiang Mai); DT1: Dong Tao (Udon Thani); DT2: Dong Tao (Lopburi); FC: Mixing-fighting cock (Bangken); Koi: Lao Pa Koi (Lamphun); KP: Khaew Paree (Phitsanulok); LHK3: Lueng Hang Khao (Phitsanulok Panyanukun School); MHS1: Mae Hong Son (Chiang Mai); MHS2: Mae Hong Son (Mae Hong Son Farmer); MHS3: Mae Hong Son (MRLBC); PDH2: Pradu Hang Dam (Phitsanulok 2); PDH3: Pradu Hang Dam (Chiang Mai); PDH4: Pradu Hang Dam (Nakhon Prathom); SD1: Samae Dam (Uthai 1); SD2: Samae Dam (Uthai 2); WZ: Wein Chang (Udon Thani); Gsai: *G. gallus Spadiceus* (Huai Sai); KK: *G. gallus Spadiceus* (Khao Kho); SSkl: *G. gallus spadiceus* (Songkhla); Gskl: *G. gallus gallus* (Songkhla); CMZ: *G. gallus gallus* (Chiang Mai); Gct: *G. gallus gallus* (Chanthaburi); Gre: *G. gallus gallus* (Roi Et); Gskw: *G. gallus gallus* (Sa Kaew); Gsskt: *G. gallus gallus* (SiSaket); Ssai: *G. gallus gallus* (Huai Sai)

Table S6 Mutation types and their locations in the partial fragments of the *BG1* gene exon 16.

No	Allele	Position of mutation and nucleotide substitution	Mutation type	Amino acid change (nucleotide substitution)
1	*BG1*VN-TH25, BG1*TH79, BG1*TH84, BG1*TH96*	23G>T	Silent	-
	*BG1*VN-TH32, BG1*TH64*	23G>A	Silent	-
2	*BG1*TH83*	27G>T	Silent	-
3	*BG1*VN-TH23, BG1*VN-TH59, BG1*TH97*	29G>T	Silent	-
4	*BG1*VN-TH2, BG1*VN-TH57, BG1*TH61,*	31T>A	Silent	-
5	*BG1*TH89*	34A>G	Silent	-
6	*BG1*TH83*	36C>T	Silent	-
7	*BG1*VN3, BG1*VN-TH5, BG1*VN-TH15, BG1*VN21, BG1*VN-TH29, BG1*VN-TH42, BG1*VN-TH44, BG1*VN48, BG1*VN-TH49, BG1*TH64, BG1*TH67, BG1*TH69, BG1*TH76*	37C>T	Silent	-
8	*BG1*VN3, BG1*VN-TH5, BG1*VN-TH10, BG1*VN-TH15, BG1*VN-TH20, BG1*VN-TH26, BG1*VN-TH29, BG1*VN-TH31, BG1*VN-TH32, BG1*VN-TH36, BG1*VN-TH38, BG1*VN48, BG1*VN-TH49, BG1*TH64, BG1*TH67, BG1*TH69, BG1*TH70, BG1*TH86, BG1*TH93, BG1*TH96*	38C>G	Silent	-
	*BG1*VN-TH7,*	38C>T	Silent	-
	*BG1*TH68*	38C>A	Silent	-
9	*BG1*VN-TH2, BG1*VN3, BG1*VN-TH5, BG1*VN-TH7, BG1*VN-TH10, BG1*VN-TH15, BG1*VN-TH20, BG1*VN-TH26, BG1*VN-TH29, BG1*VN-TH31, BG1*VN-TH32, BG1*VN-TH36, BG1*VN-TH38, BG1*VN48, BG1*VN-TH49, BG1*TH61, BG1*TH64, BG1*TH67-70, BG1*TH86, BG1*TH93, BG1*TH96*	39A>G	Silent	-
10	*BG1*VN-TH25, BG1*VN-TH35,*	41G>A	Silent	-
11	*BG1*VN-TH6, BG1*VN-TH22, BG1*VN43, BG1*VN-TH45, BG1*VN-TH52, BG1*VN-TH58, BG1*TH74*	42T>G	Silent	-
12	*BG1*TH86*	43A>G	Silent	-
13	BG1*TH68	46A>G	Silent	-
14	*BG1*VN-TH16*	47G>A	Silent	-
15	*BG1*VN-TH35, BG1*VN-TH52, BG1*TH65,*	48C>G	Silent	-

No	Allele	Position of mutation and nucleotide substitution	Mutation type	Amino acid change (nucleotide substitution)
16	*BG1*VN-TH52, BG1*VN-TH58, BG1*TH65,*	49A>G	Silent	-
17	*BG1*VN-TH35, BG1*TH64,*	50G>T	Silent	-
	*BG1*TH65*	50G>C	Silent	-
	*BG1*TH82*	50G>A	Silent	-
18	*BG1*TH68*	51T>C	Silent	-
19	*BG1*TH92*	52G>C	Silent	-
20	*BG1*VN-TH16*	53G>C	Silent	-
21	*BG1*VN-TH35, BG1*TH65*	54G>A	Silent	-
22	*BG1*VN-TH10, BG1*VN-TH25, BG1*VN-TH31, BG1*VN-TH36, BG1*VN-TH45*	55G>A	Silent	-
23	*BG1*VN-TH1, BG1*TH79*	57G>A	Silent	-
	*BG1*VN-TH35, BG1*VN-TH57, BG1*TH65*	57G>C	Silent	-
24	*BG1*VN-TH30*	58G>A	Silent	-
25	*BG1*VN-TH25, BG1*VN-TH45*	59G>T	Silent	-
26	*BG1*TH66*	60A>G	Silent	-
27	*BG1*VN-TH1, BG1*VN-TH16, BG1*VN-TH35, BG1*TH65,*	61T>G	Silent	-
28	*BG1*VN-TH2*	64G>C	Silent	-
29	*BG1*VN-TH8*	65G>C	Silent	-
	*BG1*TH66*	65G>T	Silent	-
30	*BG1*VN-TH5, BG1*TH61, BG1*TH67, BG1*TH87,*	68G>A	Silent	-
31	*BG1*VN-TH6, BG1*VN-TH8, BG1*VN-TH15, BG1*VN-TH17, BG1*VN-TH22, BG1*VN-TH29, BG1*VN-TH39, BG1*VN-TH41, BG1*VN43, BG1*VN-TH45-47, BG1*VN-TH51, BG1*TH63, BG1*TH74, BG1*TH79, BG1*TH88,*	72T>C	Silent	-
	*BG1*TH92*	72T>G	Silent	-
32	*BG1*VN-TH6, BG1*VN-TH15, BG1*VN-TH22, BG1*VN-TH29, BG1*VN43, BG1*VN-TH45, BG1*TH63, BG1*TH74,*	73G>A	Silent	-

No	Allele	Position of mutation and nucleotide substitution	Mutation type	Amino acid change (nucleotide substitution)
33	*BG1*VN-TH37, BG1*TH72, BG1*TH98*	75G>T	Silent	-
34	*BG1*VN-TH17, BG1*VN-TH46*	76G>C	Silent	-
35	*BG1*VN-TH2, BG1*VN-TH16, BG1*VN-TH20, BG1*VN21, BG1*VN-TH38, BG1*VN-TH39, BG1*VN-TH42, BG1*VN-TH44, BG1*VN47, BG1*VN-TH52, BG1*VN54, BG1*VN-TH58, BG1*TH75, BG1*TH77, BG1*TH88*	84G>C	Silent	-
	*BG1*VN-TH23, BG1*VN-TH27, BG1*VN-TH28, BG1*VN-TH53, BG1*VN-TH59, BG1*TH79, BG1*TH97*	84G>T	Silent	-
36	*BG1*VN-TH12, BG1*VN-TH23, BG1*VN-TH27, BG1*VN-TH28, BG1*VN-TH53, BG1*VN-TH59, BG1*TH97*	88G>C	Silent	-
	*BG1*VN-TH16*	88G>A	Silent	-
37	*BG1*VN-TH6, BG1*VN-TH22, BG1*VN24, BG1*VN-TH45, BG1*TH63*	90G>A	Silent	-
38	*BG1*VN-TH12, BG1*VN-TH23, BG1*VN-TH27, BG1*VN-TH28, BG1*VN-TH53, BG1*VN-TH59, BG1*TH97*	91G>A	Silent	-
39	*BG1*VN21, BG1*VN-TH42, BG1*VN-TH44, BG1*TH76*	94G>A	Silent	-
40	*BG1*TH78*	102C>G	Silent	-
41	*BG1*VN-TH51*	106C>G	Silent	-
42	*BG1*VN-TH6, BG1*VN-TH35, BG1*TH68, BG1*TH75, BG1*TH81, BG1*TH88, BG1*TH92, BG1*TH98*	108C>A	Silent	-
43	*BG1*VN-TH1, BG1*TH75, BG1*TH83, BG1*TH84, BG1*TH88, BG1*TH98*	109C>T	Silent	-
44	*BG1*VN-TH41*	110G>C	Silent	-
	*BG1*VN-TH26, BG1*VN-TH30, BG1*TH68, BG1*TH79*	110G>A	Silent	-
45	*BG1*TH79*	112G>T	Silent	-
46	*BG1*VN-TH5, BG1*VN24, BG1*VN-TH37, BG1*TH66, BG1*TH67, BG1*TH73, BG1*TH78, BG1*TH82*	116T>C	Silent	-
47	*BG1*VN-TH5*	117G>A	Silent	-
48	*BG1*VN-TH6, BG1*VN-TH8, BG1*VN-TH22, BG1*VN-TH40, BG1*VN-TH45, BG1*TH63, BG1*TH74, BG1*TH80*	123G>A	Silent	-

No	Allele	Position of mutation and nucleotide substitution	Mutation type	Amino acid change (nucleotide substitution)
49	*BG1*TH83*	125A>G	Silent	-
50	*All alleles*	128G>A	Silent	-
51	*BG1*TH75, BG1*TH88*	130A>T	Silent	-
	*BG1*VN-TH18, BG1*VN-TH41*	130A>G	Silent	-
52	*BG1*VN-TH25, BG1*VN33, BG1*TH62,*	131G>A	Silent	-
53	*All alleles*	132G>A	Silent	-
54	*BG1*TH75, BG1*TH88*	133C>T	Silent	-
	*BG1*TH85, BG1*TH91*	133C>G	Silent	-
55	*BG1*VN-TH4, BG1*VN-TH6, BG1*VN-TH16, BG1*VN-TH17, BG1*VN-TH22, BG1*VN-TH31, BG1*VN-TH45, BG1*VN-TH46, BG1*VN-TH52, BG1*VN-TH53, BG1*VN-TH58, BG1*TH63, BG1*TH72, BG1*TH74, BG1*TH77, BG1*TH96*	135G>A	Silent	-
56	*BG1*VN21, BG1*VN-TH42, BG1*VN-TH44, BG1*TH76*	139T>A	Silent	-
57	*BG1*VN-TH36, BG1*VN-TH53, BG1*TH72, BG1*TH93*	141C>G	Silent	-
58	*BG1*VN-TH4, BG1*VN-TH6, BG1*VN-TH16-18, BG1*VN-TH22, BG1*VN-TH34, BG1*VN-TH36, BG1*VN-TH41, BG1*VN-TH45, BG1*VN-TH46, BG1*VN-TH49, BG1*VN-TH52, BG1*VN-TH53, BG1*VN-TH58, BG1*TH63, BG1*TH72, BG1*TH74, BG1*TH77, BG1*TH81, BG1*TH84, BG1*TH93*	142T>C	Silent	-
59	*BG1*VN-TH14, BG1*VN-TH25,*	143A>T	Silent	-
60	*BG1*VN-TH57, BG1*TH73, BG1*TH83*	144C>G	Silent	-
61	*BG1*VN-TH16*	145C>A	Silent	-
62	*BG1*TH68, BG1*TH89*	147A>C	Silent	-
	*BG1*VN-TH18, BG1*VN-TH41, BG1*TH65, BG1*TH81*	147A>G	Silent	-
	*BG1*TH92*	147A>T	Silent	-
63	*BG1*TH64*	148A>G	Silent	-

No	Allele	Position of mutation and nucleotide substitution	Mutation type	Amino acid change (nucleotide substitution)
64	*BG1*VN-TH4, BG1*VN-TH6, BG1*VN-TH17, BG1*VN-TH22, BG1*VN-TH36, BG1*VN-TH45, BG1*VN-TH46, BG1*VN-TH52, BG1*VN-TH53, BG1*VN-TH58, BG1*TH63, BG1*TH72, BG1*TH74, BG1*TH77, BG1*TH93*	149A>T	Silent	-
65	*BG1*VN3, BG1*VN-TH4, BG1*VN-TH6, BG1*VN-TH14, BG1*VN-TH17, BG1*VN-TH19, BG1*VN-TH22, BG1*VN-TH30, BG1*VN33-36, BG1*VN-TH41, BG1*VN-TH45, BG1*VN-TH46, BG1*VN-TH49, BG1*VN-TH52-56, BG1*VN-TH58, BG1*TH62, BG1*TH63, BG1*TH68, BG1*TH69, BG1*TH72, BG1*TH74, BG1*TH77-79, BG1*TH82, BG1*TH84, BG1*TH85, BG1*TH89, BG1*TH91, BG1*TH93*	152G>C	Silent	-
66	*BG1*VN-TH1, BG1*VN-TH2, BG1*VN-TH5, BG1*VN-TH7-12, BG1*VN-TH15, BG1*VN-TH16, BG1*VN-TH18, BG1*VN21, BG1*VN-TH23-29, BG1*VN-TH31, BG1*VN-TH32, BG1*VN-TH37, BG1*VN-TH40, BG1*VN-TH42-44, BG1*VN48, BG1*VN-TH57, BG1*VN-TH59-61, BG1*TH64-67, BG1*TH70, BG1*TH71, BG1*TH73, BG1*TH75, BG1*TH76, BG1*TH80, BG1*TH81, BG1*TH83, BG1*TH86, BG1*TH88, BG1*TH90, BG1*TH92, BG1*TH94-98*	153A>G	Silent	-
67	*BG1*VN-TH7, BG1*VN-TH10, BG1*VN-TH12, BG1*VN-TH23, BG1*VN-TH25, BG1*VN-TH27, BG1*VN-TH28, BG1*VN-TH36, BG1*VN-TH59, BG1*TH64, BG1*TH93, BG1*TH97*	154C>T	Silent	-
	*BG1*VN-TH34, BG1*VN-TH49, BG1*VN-TH56, BG1*TH68, BG1*TH82, BG1*TH89*	154C>A	Silent	-
68	*BG1*VN-TH1, BG1*VN-TH2, BG1*VN-TH7, BG1*VN-TH10-13, BG1*VN-TH18, BG1*VN-TH23, BG1*VN-TH25, BG1*VN-TH27, BG1*VN-TH28, BG1*VN-TH37, BG1*VN-TH59, BG1*TH64, BG1*TH75, BG1*TH81, BG1*TH88, BG1*TH90, BG1*TH97, BG1*TH98*	155A>G	Silent	-
69	*BG1*VN3, BG1*VN-TH4, BG1*VN-TH6, BG1*VN-TH17, BG1*VN-TH19, BG1*VN-TH22, BG1*VN-TH30, BG1*VN33, BG1*VN-TH35, BG1*VN-TH36, BG1*VN-TH41, BG1*VN-TH45, BG1*VN-TH46, BG1*VN-TH52-55, BG1*VN-TH58, BG1*TH62, BG1*TH63, BG1*TH69, BG1*TH72, BG1*TH74, BG1*TH77-79, BG1*TH84, BG1*TH85, BG1*TH87, BG1*TH91, BG1*TH93*	156T>C	Silent	-
	*BG1*TH90*	156T>A	Silent	-
70	*BG1*VN3, BG1*VN-TH14, BG1*VN33, BG1*VN54, BG1*TH62, BG1*TH69, BG1*TH85, BG1*TH87, BG1*TH91*	157C>G	Silent	-
	*BG1*VN-TH4, BG1*VN-TH6, BG1*VN-TH17, BG1*VN-TH19, BG1*VN-TH22, BG1*VN-TH30, BG1*VN-TH35, BG1*VN-TH36, BG1*VN-TH41, BG1*VN-TH45, BG1*VN-TH46, BG1*VN-TH52, BG1*VN-TH53, BG1*VN-TH55, BG1*VN-TH58, BG1*TH63, BG1*TH72, BG1*TH74, BG1*TH77-79, BG1*TH84, BG1*TH93*	157C>A	Silent	-

No	Allele	Position of mutation and nucleotide substitution	Mutation type	Amino acid change (nucleotide substitution)
	*BG1*VN-TH5, BG1*VN-TH8, BG1*VN-TH9, BG1*VN-TH15, BG1*VN24, BG1*VN-TH26, BG1*VN-TH29, BG1*VN-TH31, BG1*VN-TH32, BG1*VN-TH40, BG1*VN43, BG1*VN48, BG1*VN-TH60, BG1*TH61, BG1*TH66, BG1*TH67, BG1*TH70, BG1*TH73, BG1*TH75, BG1*TH80, BG1*TH83, BG1*TH88, BG1*TH96*	157C>T	Silent	-
71	*BG1*VN-TH5, BG1*VN-TH8, BG1*VN-TH9, BG1*VN-TH11, BG1*VN-TH13, BG1*VN-TH15, BG1*VN-TH16, BG1*VN-TH18, BG1*VN21, BG1*VN24, BG1*VN-TH26, BG1*VN-TH29, BG1*VN-TH31, BG1*VN-TH32, BG1*VN-TH37, BG1*VN-TH40, BG1*VN-TH42-44, BG1*VN48, BG1*VN-TH57, BG1*VN-TH60, BG1*TH61, BG1*TH65-67, BG1*TH70, BG1*TH71, BG1*TH73, BG1*TH75, BG1*TH76, BG1*TH80, BG1*TH81, BG1*TH83, BG1*TH86, BG1*TH88, BG1*TH92, BG1*TH96, BG1*TH98*	159T>A	Silent	-
72	*BG1*VN-TH32*	163T>G	Silent	-
73	*BG1*VN-TH18*	164G>T	Silent	-
74	*BG1*VN-TH1, BG1*VN-TH2, BG1*VN3, BG1*VN-TH4, BG1*VN-TH6, BG1*VN-TH14, BG1*VN-TH17, BG1*VN-TH19, BG1*VN-TH22, BG1*VN-TH25, BG1*VN-TH30, BG1*VN33, BG1*VN-TH35, BG1*VN-TH36, BG1*VN-TH41, BG1*VN-TH45, BG1*VN-TH46, BG1*VN-TH52-56, BG1*VN-TH58, BG1*TH62, BG1*TH63, BG1*TH68, BG1*TH69, BG1*TH72, BG1*TH74, BG1*TH77-79, BG1*TH82, BG1*TH84, BG1*TH85, BG1*TH87, BG1*TH90, BG1*TH91, BG1*TH93*	165G>A	Silent	-
75	*BG1*VN3, BG1*VN33, BG1*TH62, BG1*TH69, BG1*TH87*	168T>G	Silent	-
76	*BG1*VN-TH15, BG1*VN-TH16, BG1*VN-TH29, BG1*VN43, BG1*TH61, BG1*TH71*	169A>T	Silent	-
77	*BG1*VN-TH1, BG1*VN-TH2, BG1*VN-TH4, BG1*VN-TH6, BG1*VN-TH17, BG1*VN-TH19, BG1*VN-TH22, BG1*VN-TH30, BG1*VN-TH35, BG1*VN-TH36, BG1*VN-TH41, BG1*VN-TH45, BG1*VN-TH46, BG1*VN-TH52-56, BG1*VN-TH58, BG1*TH63, BG1*TH72, BG1*TH74, BG1*TH77-79, BG1*TH82, BG1*TH84, BG1*TH85, BG1*TH90, BG1*TH91, BG1*TH93*	170A>G	Silent	-
78	*BG1*VN-TH1, BG1*VN-TH2, BG1*VN-TH4-6, BG1*VN-TH8, BG1*VN-TH9, BG1*VN-TH11, BG1*VN-TH13, BG1*VN-TH14, BG1*VN-TH16-19, BG1*VN21, BG1*VN-TH22, BG1*VN24, BG1*VN-TH26, BG1*VN-TH30-32, BG1*VN-TH35-37, BG1*VN-TH40-42, BG1*VN-TH44-46, BG1*VN48, BG1*VN-TH49, BG1*VN-TH52-58, BG1*VN-TH60, BG1*TH63-68, BG1*TH70-85, BG1*TH88-96, BG1*TH98*	173A>C	Silent	-
	*BG1*VN3, BG1*TH69, BG1*TH87*	173A>G	Silent	-

No	Allele	Position of mutation and nucleotide substitution	Mutation type	Amino acid change (nucleotide substitution)
79	*BG1*VN-TH5, BG1*VN-TH8, BG1*VN-TH9, BG1*VN-TH11, BG1*VN-TH13, BG1*VN-TH16, BG1*VN-TH18, BG1*VN21, BG1*VN24, BG1*VN-TH26, BG1*VN-TH32, BG1*VN-TH37, BG1*VN-TH40, BG1*VN-TH42, BG1*VN-TH44, BG1*VN48, BG1*VN-TH49, BG1*VN-TH57, BG1*VN-TH60, BG1*TH64-68, BG1*TH70, BG1*TH71, BG1*TH73, BG1*TH75, BG1*TH76, BG1*TH80, BG1*TH81, BG1*TH83, BG1*TH88, BG1*TH89, BG1*TH92, BG1*TH95, BG1*TH98*	175A>T	Silent	-
80	*BG1*TH64*	176G>T	Silent	-
81	*BG1*VN-TH46, BG1*VN-TH52, BG1*VN-TH58,*	178G>A	Silent	-
82	*BG1*VN21, BG1*VN-TH42, BG1*VN-TH44, BG1*TH76*	182G>T	Silent	-
83	*BG1*VN-TH5, BG1*VN-TH7-16, BG1*VN-TH18, BG1*VN-TH19, BG1*VN21, BG1*VN-TH23, BG1*VN24, BG1*VN-TH25-32, BG1*VN33, BG1*VN-TH34-37, BG1*VN-TH40, BG1*VN-TH42, BG1*VN43, BG1*VN-TH44, BG1*VN48, BG1*VN-TH49, BG1*VN-TH51, BG1*VN-TH56, BG1*VN-TH57, BG1*VN-TH59, BG1*VN-TH60, BG1*TH61, BG1*TH62, BG1*TH64-68, BG1*TH70, BG1*TH71, BG1*TH73, BG1*TH75, BG1*TH76, BG1*TH78-84, BG1*TH88, BG1*TH89, BG1*TH92-98*	183A>T	Silent	-
84	*BG1*VN-TH49, BG1*TH89*	187A>C	Silent	-
85	*BG1*VN-TH1, BG1*VN-TH2, BG1*VN-TH4-6, BG1*VN-TH17, BG1*VN-TH22, BG1*VN-TH26, BG1*VN-TH32, BG1*VN-TH45, BG1*VN-TH46, BG1*VN-TH53, BG1*VN-TH55, BG1*TH66, BG1*TH72, BG1*TH74, BG1*TH77*	188C>G	Silent	-
	*BG1*VN-TH34*	188C>A	Silent	-
	*BG1*TH92*	188C>T	Silent	-
86	*BG1*VN-TH49, BG1*TH73, BG1*TH89*	192A>T	Silent	-
	*BG1*VN54, BG1*TH91*	192A>C	Silent	-
87	*BG1*VN-TH1, BG1*VN-TH2, BG1*VN3, BG1*VN-TH4-19, BG1*VN21, BG1*VN-TH22, BG1*VN-TH23, BG1*VN-TH25-29, BG1*VN-TH31, BG1*VN-TH32, BG1*VN33, BG1*VN-TH34, BG1*VN-TH35, BG1*VN-TH37, BG1*VN-TH40-42, BG1*VN43, BG1*VN-TH44-46, BG1*VN48, BG1*VN-TH49, BG1*VN-TH52, BG1*VN-TH53, BG1*VN-TH55-60, BG1*TH61-90, BG1*TH94-98*	193C>G	Silent	-

No	Allele	Position of mutation and nucleotide substitution	Mutation type	Amino acid change (nucleotide substitution)
88	*BG1*VN-TH9, BG1*VN-TH14, BG1*VN-TH15, BG1*VN-TH19, BG1*VN-TH29, BG1*VN-TH35, BG1*VN-TH42, BG1*VN43, BG1*VN-TH44, BG1*VN48, BG1*VN-TH53, BG1*VN-TH57, BG1*TH61, BG1*TH64, BG1*TH65, BG1*TH71, BG1*TH76, BG1*TH84, BG1*TH86, BG1*TH96*	194C>T	Silent	-
	*BG1*VN54, BG1*TH91*	194C>A	Silent	-
89	*BG1*VN-TH8, BG1*VN-TH9, BG1*VN-TH11, BG1*VN-TH13-16, BG1*VN-TH18, BG1*VN-TH19, BG1*VN21, BG1*VN-TH22, BG1*VN-TH26, BG1*VN-TH29, BG1*VN-TH32, BG1*VN-TH35-37, BG1*VN-TH40-42, BG1*VN43, BG1*VN-TH44, BG1*VN48, BG1*VN-TH49, BG1*VN54, BG1*VN-TH55, BG1*VN-TH57, BG1*VN-TH60, BG1*TH61, BG1*TH65-68, BG1*TH70, BG1*TH71, BG1*TH75, BG1*TH76, BG1*TH78-81, BG1*TH83-86, BG1*TH88, BG1*TH89, BG1*TH91, BG1*TH93-96, BG1*TH98*	195A>G	Silent	-
	*BG1*VN24, BG1*VN-TH30,*	195A>C	Silent	-
90	*BG1*VN-TH22, BG1*VN-TH41, BG1*TH73, BG1*TH85*	196G>A	Silent	-
	*BG1*VN-TH36, BG1*TH91, BG1*TH93*	196G>C	Silent	-
	*BG1*VN54*	196Delete G	Silent	-
91	*BG1*VN-TH37, BG1*TH68, BG1*TH81, BG1*TH98*	197G>T	Silent	-
	*BG1*VN54, BG1*TH91*	197G>A	Silent	-
92	*BG1*VN-TH6, BG1*VN-TH45*	198G>T	Silent	-
	*BG1*TH73*	198G>A	Silent	-
93	*BG1*VN-TH4, BG1*VN-TH5, BG1*VN33, BG1*VN-TH36, BG1*VN54, BG1*VN-TH57, BG1*TH73, BG1*TH77, BG1*TH78, BG1*TH91*	199C>G	Silent	-
	*BG1*VN-TH30,*	199C>T	Silent	-
94	*BG1*TH64*	200T>C	Silent	-
95	*BG1*VN-TH17, BG1*VN33, BG1*VN-TH37, BG1*TH62, BG1*TH98*	202G>T	Silent	-
96	*BG1*VN-TH30*	203G>A	Silent	-
97	*BG1*VN-TH15, BG1*VN33, BG1*VN43, BG1*TH61, BG1*TH62*	204T>G	Silent	-
98	*BG1*VN-TH22*	205G>A	Silent	-

No	Allele	Position of mutation and nucleotide substitution	Mutation type	Amino acid change (nucleotide substitution)
99	*BG1*VN-TH20, BG1*VN50*	207T>G	Silent	-
100	*BG1*VN-TH13, BG1*VN-TH15, BG1*VN24, BG1*VN-TH41, BG1*VN43, BG1*TH61, BG1*TH86*	210G>A	Silent	-
	*BG1*VN-TH30,*	210G>T	Silent	-
101	*BG1*VN-TH1, BG1*VN-TH2, BG1*VN-TH7, BG1*VN-TH9, BG1*VN-TH10-12, BG1*VN-TH15, BG1*VN-TH16, BG1*VN-TH19, BG1*VN-TH25, BG1*VN-TH27-29, BG1*VN-TH34-37, BG1*VN43, BG1*VN54, BG1*VN-TH57-59, BG1*TH61, BG1*TH64, BG1*TH65, BG1*TH71, BG1*TH78, BG1*TH79, BG1*TH81, BG1*TH84-86, BG1*TH91, BG1*TH93-95, BG1*TH97, BG1*TH98*	212A>T	Silent	-
102	*BG1*VN-TH1, BG1*VN-TH2, BG1*VN-TH7, BG1*VN-TH11, BG1*VN-TH15, BG1*VN-TH16, BG1*VN-TH29, BG1*VN-TH36, BG1*VN43, BG1*VN-TH58, BG1*TH61, BG1*TH86, BG1*TH93*	215G>T	Silent	-
	*BG1*VN-TH10, BG1*VN-TH12, BG1*VN-TH25, BG1*VN-TH27, BG1*VN-TH28, BG1*VN-TH34, BG1*VN-TH57, BG1*VN-TH59, BG1*TH64, BG1*TH79, BG1*TH84, BG1*TH97*	215G>C	Silent	-
	*BG1*TH63*	215G>A	Silent	-
103	*BG1*VN-TH37, BG1*VN-TH59, BG1*TH82, BG1*TH98*	216G>A	Silent	-
104	*BG1*VN-TH30*	218C>T	Silent	-
105	*BG1*VN-TH16*	219C>A	Silent	-
106	*BG1*VN54, BG1*TH85, BG1*TH91*	220T>G	Silent	-
107	*BG1*VN-TH5, BG1*VN-TH60, BG1*TH67*	221C>T	Silent	-
108	*BG1*VN24, BG1*TH75, BG1*TH88*	223C>T	Silent	-
109	*BG1*VN-TH12, BG1*VN-TH14, BG1*VN-TH20, BG1*VN-TH27, BG1*VN-TH28, BG1*VN-TH38, BG1*VN-TH39, BG1*VN47, BG1*VN50, BG1*VN-TH51, BG1*VN-TH59, BG1*TH73*	224T>C	Silent	-
110	*BG1*VN-TH38*	225G>A	Silent	-
111	*BG1*VN-TH10, BG1*VN24, BG1*VN-TH25, BG1*TH97*	228C>T	Silent	-
112	*BG1*TH74*	229C>A	Silent	-
113	*BG1*VN54, BG1*TH85, BG1*TH91*	231T>C	Silent	-
114	*BG1*VN-TH14, BG1*VN-TH18,*	232G>A	Silent	-
115	*BG1*VN-TH14, BG1*VN-TH19,*	233G>A	Silent	-

No	Allele	Position of mutation and nucleotide substitution	Mutation type	Amino acid change (nucleotide substitution)
116	*BG1*VN-TH4, BG1*VN-TH7, BG1*VN-TH8, BG1*VN-TH13, BG1*VN-TH14, BG1*VN-TH19, BG1*VN-TH22, BG1*VN24, BG1*VN-TH30, BG1*VN-TH31, BG1*VN33, BG1*VN-TH34, BG1*VN-TH35, BG1*VN-TH37, BG1*VN-TH40, BG1*VN48, BG1*VN-TH49, BG1*VN-TH53, BG1*VN54, BG1*VN-TH55, BG1*VN-TH57, BG1*TH62, BG1*TH65, BG1*TH68, BG1*TH70, BG1*TH72-75, BG1*TH77, BG1*TH79, BG1*TH80, BG1*TH82-85, BG1*TH87-92, BG1*TH94-96, BG1*TH98*	235T>C	Silent	-
	*BG1*VN-TH9, BG1*VN-TH15, BG1*VN-TH16, BG1*VN-TH29, BG1*VN43, BG1*TH61, BG1*TH71,*	235T>G	Silent	-
117	*BG1*VN-TH30, BG1*VN54, BG1*VN-TH55, BG1*VN-TH57, BG1*TH84, BG1*TH85, BG1*TH91*	236G>A	Silent	-
	*BG1*TH77,*	236G>C	Silent	-
118	*BG1*VN54, BG1*TH85, BG1*TH91*	240C>A	Silent	-
119	*BG1*VN-TH11, BG1*VN-TH14, BG1*VN-TH57*	241C>T	Silent	-
120	*BG1*VN-TH1, BG1*VN-TH7, BG1*VN-TH34, BG1*VN-TH37, BG1*VN-TH55, BG1*VN-TH57, BG1*TH84, BG1*TH89, BG1*TH98*	242C>G	Silent	-
	*BG1*VN-TH30, BG1*VN54*	242C>T	Silent	-
	*BG1*TH73, BG1*TH90*	242C>A	Silent	-
121	*BG1*VN-TH1, BG1*VN-TH7, BG1*VN-TH34, BG1*VN-TH37, BG1*VN-TH55, BG1*VN-TH57, BG1*TH73, BG1*TH84, BG1*TH89, BG1*TH90, BG1*TH98*	243C>T	Silent	-
122	*BG1*VN-TH1, BG1*VN-TH4, BG1*VN-TH7, BG1*VN-TH18, BG1*VN21, BG1*VN-TH31, BG1*VN-TH34, BG1*VN-TH37, BG1*VN-TH42, BG1*VN-TH44, BG1*VN-TH55, BG1*VN-TH57, BG1*TH64, BG1*TH65, BG1*TH70, BG1*TH72, BG1*TH73, BG1*TH76, BG1*TH77, BG1*TH79, BG1*TH84, BG1*TH89, BG1*TH90, BG1*TH94-96, BG1*TH98*	244A>G	Silent	-
123	*BG1*VN-TH1, BG1*VN-TH49*	246C>A	Silent	-
124	*BG1*VN-TH17, BG1*VN-TH35*	247C>T	Silent	-
125	*BG1*VN-TH2, BG1*VN-TH4-6, BG1*VN-TH9, BG1*VN-TH11, BG1*VN-TH15, BG1*VN-TH16, BG1*VN24, BG1*VN-TH29, BG1*VN43, BG1*VN-TH45, BG1*TH61, BG1*TH63, BG1*TH67, BG1*TH71, BG1*TH72, BG1*TH77*	250C>T	Silent	-
126	*BG1*VN-TH1, BG1*VN-TH2, BG1*VN3, BG1*VN-TH5, BG1*VN-TH7-20, BG1*VN21, BG1*VN-TH22, BG1*VN-TH23, BG1*VN-TH25-32, BG1*VN33, BG1*VN-TH34-42, BG1*VN43, BG1*VN-TH44, BG1*VN-TH46, BG1*VN47, BG1*VN48, BG1*VN-TH49, BG1*VN50, BG1*VN-*	251G>A	Silent	-

No	Allele	Position of mutation and nucleotide substitution	Mutation type	Amino acid change (nucleotide substitution)
	*TH51-53, BG1*VN54, BG1*VN-TH55-60, BG1*TH61, BG1*TH62, BG1*TH64-71, BG1*TH73-76, BG1*TH78-98*			
	*BG1*VN-TH4, BG1*VN-TH6, BG1*VN24, BG1*VN-TH45, BG1*TH63, BG1*TH72, BG1*TH77,*	251G>C	Silent	-
127	*BG1*VN-TH4, BG1*VN-TH6, BG1*VN24, BG1*VN-TH45, BG1*TH63, BG1*TH72, BG1*TH77*	252G>A	Silent	-
128	*BG1*VN-TH26, BG1*VN-TH32, BG1*TH66, BG1*TH79*	253C>T	Silent	-
129	*BG1*VN-TH2, BG1*VN-TH9, BG1*VN-TH15, BG1*VN-TH16, BG1*VN-TH29, BG1*VN43, BG1*VN-TH60, BG1*TH61, BG1*TH67, BG1*TH71,*	259C>G	Silent	-
130	*BG1*VN-TH32*	260T>A	Silent	-
131	*BG1*VN3, BG1*VN-TH31, BG1*VN33, BG1*VN-TH49, BG1*TH62, BG1*TH69, BG1*TH75, BG1*TH79, BG1*TH87, BG1*TH88,*	264C>T	Silent	-
132	*BG1*VN-TH15, BG1*VN-TH29, BG1*TH61,*	266A>G	Silent	-
133	*BG1*VN-TH13, BG1*TH68, BG1*TH83, BG1*TH86*	268G>T	Silent	-
134	*BG1*TH89*	269C>G	Silent	-
135	*BG1*VN-TH30, BG1*TH90*	271C>T	Silent	-
136	*BG1*TH89*	273G>A	Silent	-
137	*BG1*VN3, BG1*VN-TH10, BG1*VN-TH25, BG1*VN-TH28, BG1*VN33, BG1*TH62, BG1*TH75, BG1*TH87, BG1*TH88, BG1*TH97*	278C>T	Silent	-
138	*BG1*VN3, BG1*VN-TH10, BG1*VN-TH25, BG1*VN-TH28, BG1*VN33, BG1*TH62, BG1*TH69, BG1*TH75, BG1*TH87, BG1*TH88, BG1*TH97*	279C>G	Silent	-
139	*BG1*VN-TH31*	280C>T	Silent	-
140	*BG1*VN-TH1, BG1*VN-TH2, BG1*VN-TH5, BG1*VN-TH56, BG1*TH65, BG1*TH68, BG1*TH70*	281A>C	Silent	-
	*BG1*VN-TH55,*	281A>T	Silent	-
141	*BG1*VN-TH30*	282C>G	Silent	-
142	*BG1*VN54, BG1*TH85, BG1*TH91*	283A>C	Silent	-
143	*BG1*TH64, BG1*TH90*	284G>A	Silent	-
144	*BG1*VN-TH11*	287C>T	Silent	-

No	Allele	Position of mutation and nucleotide substitution	Mutation type	Amino acid change (nucleotide substitution)
145	*BG1*TH65, BG1*TH77, BG1*TH91*	298T>G	Silent	-
146	*BG1*VN-TH42, BG1*TH76*	299C>G	Missense	Gly to Ala (TCC to TGC)
147	*BG1*VN-TH49, BG1*TH79, BG1*TH89*	301C>T	Silent	-
148	*BG1*VN-TH2, BG1*VN3, BG1*VN-TH4-8, BG1*VN-TH10, BG1*VN-TH11, BG1*VN-TH16, BG1*VN-TH19, BG1*VN21, BG1*VN-TH22, BG1*VN-TH23, BG1*VN24, BG1*VN-TH25, BG1*VN-TH31, BG1*VN33, BG1*VN-TH35, BG1*VN-TH36, BG1*VN-TH39, BG1*VN-TH40, BG1*VN-TH44, BG1*VN-TH45, BG1*VN48, BG1*VN-TH51, BG1*VN-TH56, BG1*VN-TH57, BG1*TH61-64, BG1*TH67-69, BG1*TH73, BG1*TH74, BG1*TH77, BG1*TH80, BG1*TH86, BG1*TH87, BG1*TH90, BG1*TH96, BG1*TH97*	302G>A	Missense	Pro to Leu (CGG to CAG)
149	*BG1*VN-TH49, BG1*TH89*	303G>A	Missense	Pro to Ser (CGG to TGA)
150	*BG1*VN-TH26, BG1*VN-TH32*	304C>G	Missense	Gln to His (CTG to GTG)
151	*BG1*TH74*	306G>C	Missense	Gln to Glu (CTG to CTC)
152	*BG1*VN-TH35*	307G>A	Silent	-
153	*BG1*VN-TH35*	310G>A	Silent	-
154	*BG1*VN-TH5, BG1*VN-TH7, BG1*VN-TH11, BG1*VN-TH55, BG1*TH67, BG1*TH81*	311A>T	Missense	Leu to His (GAG to GTG)
155	*BG1*VN3, BG1*TH69, BG1*TH87*	312G>A	Missense	Leu to Phe (GAG to GAA)
156	*BG1*TH61*	320G>A	Missense	Ala to Val (AGC to AAC)
157	*BG1*VN-TH11, BG1*VN-TH15, BG1*VN-TH19, BG1*VN21, BG1*VN-TH29, BG1*VN33, BG1*VN-TH42, BG1*VN-TH44, BG1*TH62, BG1*TH75, BG1*TH76, BG1*TH78, BG1*TH88, BG1*TH98*	324C>T	Missense	Val to Met (CAC to CAT)
	*BG1*VN-TH17*	324C>G	Missense	Val to Leu (CAC to CAG)
158	*BG1*VN3, BG1*TH69, BG1*TH83, BG1*TH84, BG1*TH87*	325G>A	Silent	-

No	Allele	Position of mutation and nucleotide substitution	Mutation type	Amino acid change (nucleotide substitution)
	*BG1*VN-TH34*	325G>T	Silent	-
159	*BG1*VN-TH10, BG1*VN-TH18, BG1*VN-TH25*	332G>A	Missense	Pro to Leu (AGG to AAG)
160	*BG1*VN-TH57*	333G>T	Missense	Pro to Thr (AGG to AGT)
161	*BG1*VN-TH10, BG1*VN-TH18, BG1*VN-TH25, BG1*TH97*	336G>T	Missense	Leu to Met (CAG to CAT)
162	*BG1*VN-TH30*	337G>A	Silent	-
	*BG1*TH86*	337G>T	Silent	-
163	*BG1*VN-TH18*	338A>G	Missense	Leu to Pro (GAG to GGG)
164	*BG1*TH73*	339G>A	Missense	Leu to Phe (GAG to GAA)
165	*BG1*VN-TH16*	340G>A	Silent	-
	*BG1*VN-TH17*	340G>T	Silent	-
166	*BG1*TH89*	343C>T	Silent	-
167	*BG1*VN-TH49*	347C>G	Missense	Trp to Ser (CCA to CGA)
168	*BG1*VN-TH31, BG1*TH80, BG1*TH96*	349G>C	Silent	-
169	*BG1*VN-TH1, BG1*VN3, BG1*VN-TH4-19, BG1*VN21, BG1*VN-TH22, BG1*VN24, BG1*VN-TH25, BG1*VN-TH29-31, BG1*VN33, BG1*VN-TH36, BG1*VN-TH37, BG1*VN-TH41, BG1*VN-TH42, BG1*VN43, BG1*VN-TH44-46, BG1*VN48, BG1*VN-TH49, BG1*VN50, BG1*VN-TH52, BG1*VN-TH53, BG1*VN54, BG1*VN-TH55-58, BG1*TH61-69, BG1*TH71-80, BG1*TH82-89, BG1*TH91-98*	352A>G	Silent	-
	*BG1*VN-TH32, BG1*TH90*	352A>C	Silent	-
170	*BG1*TH66*	355G>A	Missense	Thr to Ala (GGT to AGC)

No	Allele	Position of mutation and nucleotide substitution	Mutation type	Amino acid change (nucleotide substitution)
171	*BG1*VN-TH10, BG1*VN-TH25, BG1*VN-TH26, BG1*VN-TH40, BG1*VN-TH55, BG1*TH78, BG1*TH80, BG1*TH85, BG1*TH91, BG1*TH97*	357T>C	Missense	Thr to Ala (GGT to AGC)
172	*BG1*VN-TH26*	358G>A	Silent	-

Note: The *BG1* gene is located on the negative strand; thus, the genomic mutation corresponds to the reverse complement of the intended coding change. The pieces of Thai and Vietnamese indigenous and local chicken breeds and red junglefowl were compared with the reference sequence (accession number: OM953775)

Table S7. The results of the Analysis of Variance (AMOVA) for indigenous and local chicken populations and red junglefowl in Thailand and Vietnam

**Source**		**df**	**SS**	**Var**	**% of variance**
Vietnam indigenous	Among Population	10	20245.4	2024.5	26.4
	Among Individual	265	56337.4	212.6	73.6
	Within Individual	NA	NA	NA	NA
	Total	275	76582.7	2237.1	100
Vietnam red junglefowl	Among Population	1	280.5	280.5	3.10
	Among Individual	20	8778.3	438.9	96.9
	Within Individual	1	0.50	0.50	0.01
	Total	22	9059.3	719.9	100
Overal mean	Among Population	12	20589.8	1715.8	24.0
	Among Individual	285	65115.7	228.5	76.0
	Within Individual	1	0.50	0.50	0.0
	Total	298	85706.0	1944.8	100
Thailand indigenous	Among Population	34	64539.0	1898.2	11.3
	Among Individual	667	505517.7	757.9	88.7
	Within Individual	NA	NA	NA	NA
	Total	701	570056.7	2656.1	100
Thailand red junglefowl	Among Population	7	9846.0	1406.6	9.15
	Among Individual	198	97796.8	493.9	90.9
	Within Individual	NA	NA	NA	NA
	Total	205	107642.9	1900.5	100
Overal mean	Among Population	41	75544.7	1842.6	11.1
	Among Individual	866	606225.6	700.0	88.9
	Within Individual	NA	NA	NA	NA
	Total	907	681770.4	2542.6	100

(df: degree of freedom *f*; SS: sum of squares; Var: variance components)

Table S8. Detailed site-by-site results from the MEME analysis based on the alleles of the *BG1* gene

Part	Codon	α	β1	p1	β+	p+	LRT	*p*-value	Branches under selection	q	Class
1	1	0.000	0.000	1.000	0.000	0.000	0.000	1.000	0	1	Invariable
1	2	0.000	0.000	1.000	0.000	0.000	0.000	1.000	0	1	Invariable
1	3	0.000	0.000	1.000	0.000	0.000	0.000	1.000	0	1	Invariable
1	4	0.000	0.000	1.000	0.000	0.000	0.000	1.000	0	1	Invariable
1	5	0.000	0.000	1.000	0.000	0.000	0.000	1.000	0	1	Invariable
1	6	0.000	0.000	1.000	0.000	0.000	0.000	1.000	0	1	Invariable
1	7	25.472	0.000	1.000	0.000	0.000	0.000	0.667	0	1	Neutral
1	8	12.984	1.069	0.000	21.259	1.000	0.252	0.508	0	1	Neutral
1	9	50.882	0.000	1.000	6.092	0.000	0.000	0.667	0	1	Neutral
1	10	12.959	0.000	1.000	0.747	0.000	0.000	0.667	0	1	Neutral
1	11	0.133	0.007	0.040	3.901	0.960	0.008	0.644	1	1	Neutral
1	12	7.464	0.000	1.000	1.034	0.000	0.000	0.667	0	1	Neutral
1	13	25.908	0.000	1.000	0.700	0.000	0.000	0.667	0	1	Neutral
1	14	25.914	8.911	0.513	10.829	0.487	0.000	0.667	0	1	Neutral
1	15	0.000	0.000	0.010	5.598	0.990	1.146	0.292	1	1	Neutral
1	16	0.008	0.001	0.000	8.374	1.000	2.063	0.176	1	1	Neutral
1	17	0.000	0.000	1.000	0.000	0.000	0.000	1.000	0	1	Invariable
1	18	25.116	0.000	1.000	2.954	0.000	0.000	0.667	0	1	Neutral
1	19	0.000	0.000	0.010	10.883	0.990	1.674	0.217	3	1	Neutral
1	20	0.000	0.000	0.010	3.340	0.990	0.862	0.344	1	1	Neutral
1	21	0.000	0.000	10.000	0.000	0.000	0.000	1.000	0	1	Invariable
1	22	0.000	0.000	1.000	0.000	0.000	0.000	1.000	0	1	Invariable
1	23	12.512	0.410	0.001	24.735	0.999	0.389	0.462	0	1	Neutral
1	24	12.712	0.000	1.000	1.596	0.000	0.000	0.667	0	1	Neutral
1	25	0.001	0.000	0.001	6.122	0.999	0.509	0.427	1	1	Neutral
1	26	7.390	0.264	0.001	21.224	0.999	1.037	0.311	0	1	Neutral
1	27	7.818	0.000	0.692	12.336	0.308	0.013	0.637	1	1	Neutral
1	28	0.000	0.000	1.000	0.000	0.000	0.000	1.000	0	1	Invariable

LRT: Likelihood Ratio Test

Table S9. Detailed site-by-site results from the FEL analysis based on the alleles of the *BG1* gene

Partition	codon	α	β	α=β	LRT	*p*-value	Total branch length	class
1	1	0.000	0.000	0.000	0.000	1.0000	0.000	Invariable
1	2	0.000	0.000	0.000	0.000	1.0000	0.000	Invariable
1	3	0.000	0.000	0.000	0.000	1.0000	0.000	Invariable
1	4	0.000	0.000	0.000	0.000	1.0000	0.000	Invariable
1	5	0.000	0.000	0.000	0.000	1.0000	0.000	Invariable
1	6	0.000	0.000	0.000	0.000	1.0000	0.000	Invariable
1	7	3.859	0.000	0.417	4.371	0.0365	0.369	Neutral
1	8	1.910	3.192	2.937	0.251	0.6163	2.595	Neutral
1	9	7.743	0.000	2.069	10.149	0.0014	1.827	Purifying
1	10	1.933	0.000	0.461	2.838	0.0920	0.407	Neutral
1	11	0.000	0.585	0.585	0.008	0.9284	0.517	Neutral
1	12	1.101	0.000	0.329	2.404	0.1210	0.291	Neutral
1	13	3.866	0.000	0.685	6.789	0.0092	0.605	Purifying
1	14	3.880	1.452	2.107	0.901	0.3426	1.862	Neutral
1	15	0.000	0.837	0.472	1.147	0.2842	0.417	Neutral
1	16	0.000	1.259	0.747	2.066	0.1506	0.660	Neutral
1	17	0.000	0.000	0.000	0.000	1.0000	0.000	Invariable
1	18	3.745	0.000	1.024	5.087	0.0241	0.904	Neutral
1	19	0.000	1.634	1.234	1.677	0.1954	1.090	Neutral
1	20	0.000	0.498	0.325	0.862	0.3531	0.287	Neutral
1	21	0.000	0.000	0.000	0.000	1.0000	0.000	Invariable
1	22	0.000	0.000	0.000	0.000	1.0000	0.000	Invariable
1	23	1.933	3.725	3.225	0.389	0.5329	2.849	Neutral
1	24	1.882	0.000	0.512	2.579	0.1083	0.452	Neutral
1	25	0.000	0.909	0.801	0.509	0.4754	0.707	Neutral
1	26	1.102	3.156	2.298	1.038	0.3083	2.030	Neutral
1	27	1.145	0.514	0.812	0.455	0.5002	0.718	Neutral
1	28	0.000	0.000	0.000	0.000	1.0000	0.000	Invariable

LRT: likelihood ratio test

Table S10. Detailed site-by-site results from the Fubar analysis based on the alleles of the *BG1* gene

Site	Partition	&α	&β	&β;-&α;	Prob [&α;>&β;]	Prob [&α;<&β;]	BayesFactor [&α;<&β;]
1	1	0.616	0.392	-0.224	0.568	0.365	0.657
2	1	0.877	0.372	-0.505	0.613	0.324	0.547
3	1	0.878	0.319	-0.559	0.654	0.282	0.450
4	1	0.877	0.359	-0.518	0.623	0.314	0.523
5	1	0.616	0.416	-0.200	0.551	0.382	0.708
6	1	0.509	0.358	-0.151	0.562	0.366	0.661
7	1	4.721	0.377	-4.345	0.876	0.098	0.124
8	1	1.766	5.578	3.812	0.045	0.851	6.509
9	1	13.088	0.434	-12.654	0.999	0.000	0.001
10	1	1.817	0.415	-1.402	0.799	0.160	0.218
11	1	4.658	0.727	-3.931	0.494	0.454	0.951
12	1	1.097	0.383	-0.715	0.78	0.176	0.244
13	1	5.275	0.361	-4.913	0.953	0.034	0.040
14	1	5.267	1.412	-3.855	0.736	0.176	0.245
15	1	0.511	0.88	0.369	0.271	0.673	2.354
16	1	0.488	1.203	0.715	0.167	0.785	4.183
17	1	0.509	0.348	-0.161	0.571	0.358	0.636
18	1	5.044	0.435	-4.61	0.934	0.048	0.058
19	1	0.599	1.772	1.173	0.117	0.836	5.832
20	1	0.486	0.689	0.203	0.305	0.635	1.991
21	1	0.496	0.479	-0.017	0.477	0.456	0.956
22	1	0.877	0.359	-0.518	0.623	0.314	0.523
23	1	1.797	6.068	4.271	0.045	0.856	6.779
24	1	1.762	0.437	-1.325	0.786	0.172	0.237
25	1	0.86	0.947	0.087	0.279	0.663	2.245
26	1	1.092	4.889	3.797	0.043	0.895	9.695
27	1	1.156	0.705	-0.451	0.647	0.28	0.444
28	1	0.416	0.410	-0.006	0.469	0.457	0.962

LRT: likelihood ratio test
